# Health and economic burden of respiratory syncytial virus (RSV) disease and the cost-effectiveness of potential interventions against RSV among children under 5 years in 72 Gavi-eligible countries

**DOI:** 10.1186/s12916-020-01537-6

**Published:** 2020-04-06

**Authors:** Xiao Li, Lander Willem, Marina Antillon, Joke Bilcke, Mark Jit, Philippe Beutels

**Affiliations:** 1 Centre for Health Economics Research & Modelling Infectious Diseases, Vaccine & Infectious Disease Institute, University of Antwerp, Campus Drie Eiken, Universiteitsplein 1, Antwerp, Belgium; 2 Swiss Tropical and Public Health Institute, Socinstrasse 57, Basel, Switzerland; 3 University of Basel, Petersplatz 1, Basel, Switzerland; 4 Department of Infectious Disease Epidemiology, London School of Hygiene and Tropical Medicine, Keppel Street, Bloomsbury, London, UK

**Keywords:** Respiratory syncytial virus, Cost-effectiveness analysis, Maternal vaccination, Monoclonal antibody, Disease burden, Expected Value of Partially Perfect Information, Probabilistic sensitivity analysis, Low-income countries and lower-middle-income countries

## Abstract

**Background:**

Respiratory syncytial virus (RSV) frequently causes acute lower respiratory infection in children under 5, representing a high burden in Gavi-eligible countries (mostly low-income and lower-middle-income). Since multiple RSV interventions, including vaccines and monoclonal antibody (mAb) candidates, are under development, we aim to evaluate the key drivers of the cost-effectiveness of maternal vaccination and infant mAb for 72 Gavi countries.

**Methods:**

A static Multi-Country Model Application for RSV Cost-Effectiveness poLicy (MCMARCEL) was developed to follow RSV-related events monthly from birth until 5 years of age. MCMARCEL was parameterised using country- and age-specific demographic, epidemiological, and cost data. The interventions’ level and duration of effectiveness were guided by the World Health Organization’s preferred product characteristics and other literature. Maternal vaccination and mAb were assumed to require single-dose administration at prices assumed to align with other Gavi-subsidised technologies. The effectiveness and the prices of the interventions were simultaneously varied in extensive scenario analyses. Disability-adjusted life years (DALYs) were the primary health outcomes for cost-effectiveness, integrated with probabilistic sensitivity analyses and Expected Value of Partially Perfect Information analysis.

**Results:**

The RSV-associated disease burden among children in these 72 countries is estimated at an average of 20.8 million cases, 1.8 million hospital admissions, 40 thousand deaths, 1.2 million discounted DALYs, and US$611 million discounted direct costs. Strategy ‘mAb’ is more effective due to its assumed longer duration of protection versus maternal vaccination, but it was also assumed to be more expensive. Given all parameterised uncertainty, the optimal strategy of choice tends to change for increasing willingness to pay (WTP) values per DALY averted from the current situation to maternal vaccination (at WTP > US$1000) to mAB (at WTP > US$3500). The age-specific proportions of cases that are hospitalised and/or die cause most of the uncertainty in the choice of optimal strategy. Results are broadly similar across countries.

**Conclusions:**

Both the maternal and mAb strategies need to be competitively priced to be judged as relatively cost-effective. Information on the level and duration of protection is crucial, but also more and better disease burden evidence—especially on RSV-attributable hospitalisation and death rates—is needed to support policy choices when novel RSV products become available.

## Introduction

Respiratory syncytial virus (RSV) is one of the major causes of acute lower respiratory infection (ALRI), resulting frequently in hospitalisation (and sometimes death) in children under the age of 5 years [1, 2]. A recent systematic review estimated that 33 million RSV-ALRI cases and 118 thousand deaths occur annually in children under 5, of which 22 million cases and 103 thousand deaths are in low-income countries (LICs) and lower-middle-income-countries (LMICs) [2].

The currently available prophylactic RSV intervention (palivizumab) consists of a monthly injection over the course of the RSV season. It is only recommended for high-risk groups and only used in a few countries, partly because it is expensive [3]. However, there are multiple maternal RSV vaccine and monoclonal antibody (mAb) candidates under clinical development [4]. The most advanced maternal vaccine candidate, NCT02624947 (Novavax), has completed the phase 3 trial, but did not meet its primary endpoint of medically significant RSV lower respiratory tract infection (LRTI) [5, 6]. A new single-dose mAb candidate, MEDI8897 (Sanofi Pasteur / MedImmune), has also completed the phase IIb trial and met its primary endpoint of medically attended LRTI due to RT-PCR-confirmed RSV [7, 8].

Passive immunisation through maternal immunisation or monoclonal antibodies could potentially prevent RSV disease after birth, by protecting vulnerable infants [9]. In most LMICs and LICs, the Expanded Programme on Immunization (EPI) is well established, including paediatric and neonatal (at birth) components, and the single-dose RSV mAb, administered at birth, could be integrated into existing prevention programmes. In recent years, initiatives to administer inactive influenza vaccines and pertussis vaccines to mothers during antenatal care (ANC) visits have been successfully implemented in many high-income countries. In LICs and LMICs, the dramatic increase in the antenatal care (ANC) coverage during the past decade renders feasible the implementation of the maternal RSV vaccination programme via the ANC platform [10].

There are limited resources and funding available in LICs and LMICs, which leads to inequitable access of vaccines in these countries. The investment decisions on vaccines need to be made both at the country and a multi-country level. Gavi, the Vaccine Alliance, is an organisation that invests in vaccines to protect children’s lives and health in LICs and LMICs. Every 5 years, Gavi develops a new vaccine investment strategy (VIS) to prioritise the vaccines made available to countries throughout their vaccine support programme. RSV interventions, including both maternal vaccine and mAb, were considered as one of the six prioritised vaccine programmes as part of Gavi VIS for the 2021–2025 funding period [11].

The majority of LICs and LMICs have sparse or no data to make decisions on RSV interventions, but studies on RSV epidemiology and outcomes may represent a costly endeavour in resource-strapped settings. Therefore, it is imperative that a cost-effectiveness analysis of RSV interventions carefully evaluates the uncertainty and main drivers of implementation of RSV prophylaxis. This enables identifying the value that additional data can provide to reduce uncertainty. While RSV prevention technologies are still in development, data can be collected on its disease burden and epidemiology to inform decision-making once the technologies are available. In order to be informative at both the multi-country (i.e. Gavi, WHO) and single country levels, this analysis aims to identify the key drivers of the cost-effectiveness of potential maternal vaccination and infant monoclonal antibody strategies against RSV in 72 of the current and former Gavi-eligible countries.^1^

## Methods

### Model structure

A multi-country RSV impact model, MCMARCEL (Multi-Country Model Application for RSV Cost-Effectiveness poLicy), was developed in the R environment (https://www.r-project.org/), based on the concept of a published single-country static cohort model [12]. It follows a cohort of children monthly from birth to 5 years of age. This model was customised towards computationally efficient, multi-country applications in LIC and LMIC settings.

The model compared no RSV intervention with (1) universal RSV vaccination in the second/third trimester of pregnancy (henceforth ‘maternal’ strategy) and (2) universal RSV monoclonal antibodies at birth (henceforth ‘mAb’ strategy). The analysis is conducted from a health care payer’s perspective including only direct health care costs and effects.

**Fig. 1:**
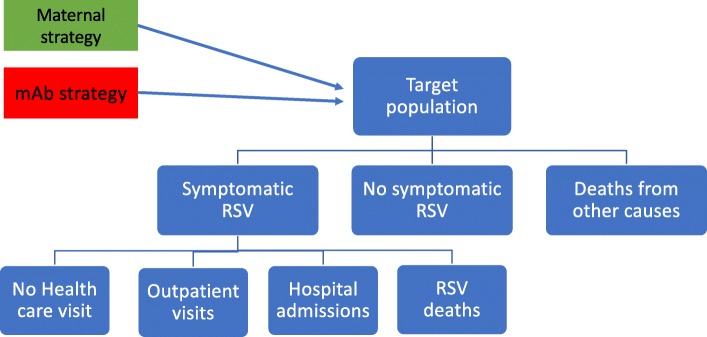
Model structure

The model structure is presented in Fig. 1. The transition probabilities depend on the country-specific and age-specific (at a monthly resolution) RSV incidence rate as well as the selected vaccine/mAb strategies.

### Demographic data

The United Nations population projections in 2020 were used to estimate live births for each country [13]. The target population for the maternal strategy was calculated based on country-specific birth rates (net of stillbirths) [14]. Country-specific mortality rates and life expectancy by age are interpolated from the United Nations database (which provides data at 5-year intervals) [13], where we fit two linear curves to interpolate the age-specific data (more details in Table 1).

**Table 1:** Data input summary

Parameter	Description	Uncertainty
Demographic data	Population projection in 2020 [13] Under-1 and under-5 mortality, life expectancy [13], and stillbirth rate [14]	Uncertainty was not taken into account
RSV disease burden	Interpolate the age-specific data (per month) and scale it per country • RSV incidence • Hospitalisation • Mortality among hospitalised patients (probability), then multiply adjustment factor 2.2 × 0.9 for overall death in both hospital and community settings	• Uncertainty around age-specific RSV incidence and hospital mortality; for details, refer to Additional file 1 • Uniform distribution is assumed for the overall death adjustment factor 2.2 (1.5–2.9) and influenza adjustment factor 0.9 (0.8–1)
Immunisation coverage	WHO BCG coverage 2016 as a proxy for both strategies	Uncertainty was not taken into account
Efficacy	70% (50–90%) [15]	Only for scenario analysis
Duration of protection	Maternal, 5 months (3–6 months) [15] mAb, 6 months (4–8 months) [12]	Only for scenario analysis
Health care seeking probabilities	WHO reported children with suspected pneumonia taken to an appropriate health provider (%) (range, 13% in Somalia to 92.3% in Ukraine) as proxies for outpatient visits [16]	Uncertainty was not taken into account
Hospital length of stay	Country-specific data, or 5.8 days (95% CI 5.3–6.4) if data were unavailable [17]	Gamma distribution (alpha = 432.52, beta = 0.014)
Treatment cost for outpatient and hospitalisation	Country-specific data, with pneumonia costs as a proxy for outpatients and hospitalisations, or adjusted from the WHO-CHOICE data [18]	Gamma distribution (for details, refer to Additional file 1)
Intervention cost in USD (including delivery cost)	Maternal, $3 for one dose mAb, $6 for one dose	Only for scenario analysis
Health outcome	Duration of illness, 11.2 days (10.1–12.3) [19] DALYs of moderate ALRI, 0.053 (0.032–0.074) [20] DALYs of severe ALRI, 0.21 (0.139–0.298) [20]	Gamma distribution Duration of illness (*α* = 398.3, *β* = 0.014) DALYs of moderate ALRI (*α* = 24.5, *β* = 0.002) DALYs of severe ALRI (*α* = 26.8, *β* = 0.008)
Discounting	3% for both costs and health outcomes	Not applicable

#### Burden of disease

The disease burden of RSV-associated ALRI in children under 5 in each country was retrieved from a recent systematic review [2]. Ten community-based studies reported age-specific incidence in LMICs, but no community-based incidence study from LICs was available. The studies were pooled, and age-specific incidence was simulated from a generalised additive mixed-effects (Poisson) regression model. Next, the percent of cases in each age group was calculated for all LMICs. Lastly, we calculated the product of the percent of cases in each age group times the country-specific overall incidence in all children under 5 years of age in order to estimate the total number of cases in each age group for each country (both LMIC and LIC).

Among the 72 Gavi-eligible countries, only two studies [21, 22] were identified where RSV-associated hospitalisation probabilities (i.e. the risk of hospitalisation for someone with RSV) were reported; however, one study in Bangladesh reported only one referral to a tertiary hospital, but did not specify whether it was RSV-associated [21]. We chose not to include this study (Homaira et al.) in our base-case analysis, because the study centres provided treatment and only referred patients to tertiary hospital if needed [21]. The hospitalisation probabilities were estimated using a generalised additive mixed-effects (logistic) regression model based on the only other study we identified, which was fit to data from Kenya (Nokes et al. [22]). Consequently, our estimated hospitalisation probability is based on less evidence (i.e. only one study) than most of the other input parameters (which are informed by at least two studies). To account for this additional uncertainty, we doubled the standard deviation from the uncertainty distribution around the estimated hospitalisation probability. In the scenario analysis, we also used pooled hospitalisation probabilities, which were based on both Nokes et al.’s and Homaira et al.’s studies [15, 21].

We found 10 studies in LIC and 19 studies in LMIC that reported age-specific hospital case-fatality ratio (hCFR) including at least three age groups. Based on sensitivity analysis (see Additional file 1), we based all hCFR rates on the LMIC data because they provided a more informative age-specific pattern in younger infants, consistent with the literature [1, 2]. We adopted the method from Shi et al. to calculate the overall RSV-associated mortality by assuming that the number of deaths in the community is on average almost the same as the within-hospital mortality [2]. As such, the overall RSV death rate = death in hospital × a community-multiplication factor of 2.2 × adjustment factor of 0.9 to account for overlapping influenza activity. More details on the methods can be found in Additional file 1.

#### Resource use and costs

In the absence of RSV-specific data, pneumonia health care seeking data were used to estimate the proportion of children with RSV-associated ALRI who did not seek health care [16], and estimates from neighbouring countries were used for countries for which such data were not available.

Due to the absence of specific cost data for RSV-associated ALRI in LICs and LMICs [23], we bridged cost data for an outpatient visit, cost per bed-day in the hospital, and length of stay in the hospital (LoS) for pneumonia from a recent systematic review [17], as 60% of the published studies on pneumonia costs were conducted in South East Asia and Africa (covering 8 of the 72 countries). Country-specific outpatient and inpatient data were used where possible. If multiple studies were available for one country, meta-analysis (using a random effect model) was performed to pool the evidence for the country. For countries without data on LoS, we used the mean of 5.8 days LoS reported for LICs and LMICs [17].

In order to estimate the RSV treatment cost for countries without available data, we first compared treatment costs predicted in the WHO CHOosing Interventions that are Cost-Effective (WHO-CHOICE) database of non-disease-specific country treatment costs (outpatient costs at primary hospital level and inpatient costs at secondary hospital level) with the cost data reported from the eight countries, as the WHO-CHOICE predictions are not disease specific. We found that the pneumonia treatment costs reported in the literature were 20-fold and 3-fold higher than the WHO-CHOICE predicted country-specific outpatients and inpatient costs, respectively [18]. Consequently, adjustment factors were calculated (one for outpatient and one for inpatient costs), with which the WHO-CHOICE costs were multiplied (see Additional file 1 for more details).

All cost data were inflated to 2016 US dollar (USD) values using local currency inflators and convertors from the World Bank, based on the ‘first inflate, then convert’ principle [24]. This implies that costs reported in USD were first converted back to their local currency value (in the reported year) before inflation and reconversion.

#### Primary health outcome

Disability-adjusted life years (DALYs) were used as the health utility metric. The base-case disability weights were derived from the Global Burden of Disease Study 2010 [20]: the values for moderate infectious disease (0.053) and severe acute episodes (0.21) were used for non-hospitalised (both none care seeking or only primary/outpatient care) and hospitalised RSV cases, respectively. The duration of illness was assumed to be 11.2 days (95% CI 10.1–12.3) based on a study conducted in Kenya [19]. The years of life lost (YLL) due to premature death were calculated from gender-specific life expectancy in years at the age of death.

#### Intervention characteristics

In the base case, we used the WHO preferred product characteristics and other literature to assume 70% efficacy (range 50–90% for scenario analysis) for both the maternal vaccine and mAb among the newborns [12, 15, 25, 26]. Recently, topline results of the first RSV maternal immunisation phase 3 trial (Prepare™) have been made public, and we applied the data in the scenario analyses. Since the duration of protection data is not available for the RSV interventions under study, we further assumed that maternal vaccination offers 5 months (3–6 months) and mAb 6 months (4–8 months) protection after birth, but varied these assumptions extensively in scenario analyses.

Country-specific Bacillus Calmette-Guérin (BCG) coverage reported in 2016 was used as a proxy for both strategies [16]. Both maternal and mAb strategies were modelled under the assumption of a single-dose intramuscular injection. The intervention costs (procurement and delivery costs) were assumed to be 3 USD for maternal and 6 USD for mAb based on prices of other Gavi-subsidised technologies [27], including both Gavi’s and each country’s contribution irrespective of the relative share of each component to the total (i.e. irrespective of the country’s position in the four Gavi transitioning phases). These intervention cost assumptions—like all other assumptions—are made independently by the authors using the sources referenced in the text. These assumptions do not reflect any current or future pricing strategies of the pharmaceutical companies taking part in the RESCEU project. A 5% wastage factor was also applied [28].

#### Outcomes and scenario analyses

The number of RSV-associated cases, hospitalisations, and deaths were presented without discounting, whereas costs and DALYs were discounted at 3% per year according to the WHO guideline. We identified the optimal strategy for each country and for a range of societal willingness to pay (WTP) values. The optimal strategy in terms of cost-effectiveness refers to the strategy that maximises the expected incremental net benefits (i.e. either maternal vaccination, mAb, or no intervention). Additionally, we estimated the degree of evidence in favour of the optimal strategy, i.e. how certain we are about the optimal strategy being ‘the best’ in terms of cost-effectiveness [29].

Uncertainty around the price, efficacy, and duration of the interventions, as well as the choice of data to inform hospitalisation probability (i.e. using pooled Nokes et al.’s and Homaira et al.’s studies instead of Nokes et al. alone), was accounted for in scenario analyses [6].

All other uncertain aspects are accounted for in a probabilistic way, i.e. 5000 random samples are drawn from pre-defined uncertainty distributions for each input parameter. The impact of probabilistic uncertainties on the results was evaluated by calculating for each uncertain input parameter the Expected Value of Partially Perfect Information (EVPPI). Our model code is available via Zenodo (see reference [30]).

## Results

### Disease burden

**Fig. 2:**
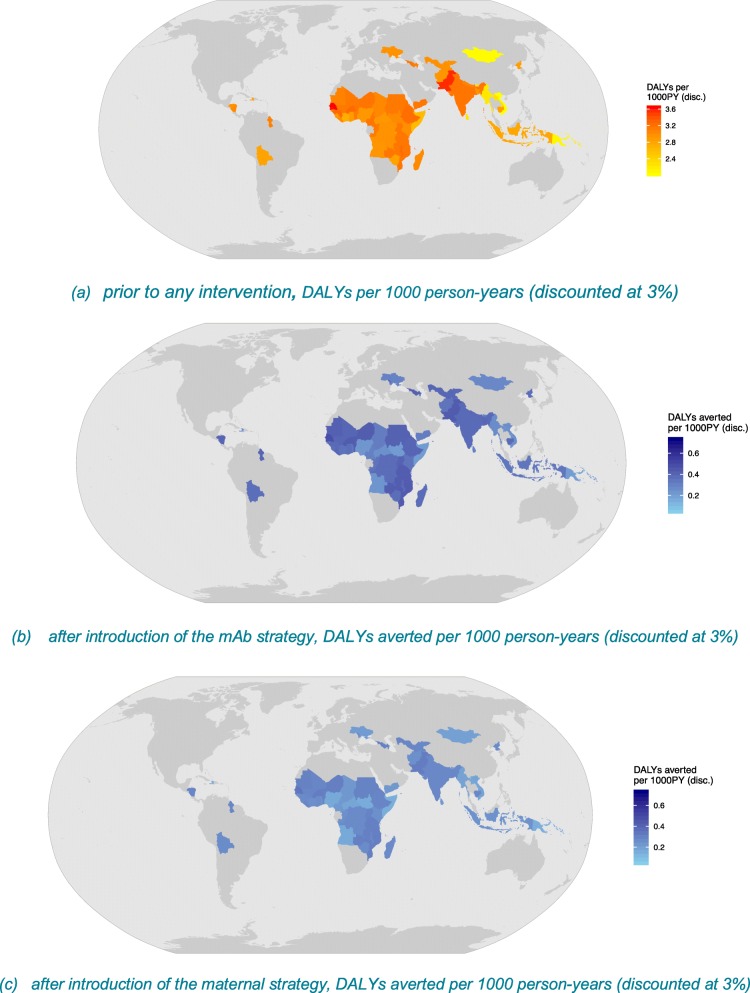
RSV-associated DALYs prior to RSV prevention and averted DALYs post-RSV prevention in 72 Gavi countries

The RSV-associated ALRI disease burden is substantial in these 72 countries. Figure 2 illustrates the burden using discounted DALYs per 1000 person-years (PY). Senegal and Pakistan have the highest burden, whereas Mongolia and Vietnam have the lowest DALYs per 1000 PY, because these countries experience the highest and lowest RSV incidence rates, respectively (Fig. 2a).

The RSV-associated disease burden is estimated at 20.8 million cases [95% prediction interval (PI) 20.8–20.9 million], leading to 1.8 million hospital admissions [95% PI 0.4–4.9 million] and 40 thousand deaths [95% PI 7–121 thousand] in the 72 countries combined. More than one third of the RSV-associated disease burden occurs in the first year of life. Using a 3% discount rate, the treatment costs total $611 million [95% PI 327–1110 million] and the DALYs 1.2 million [95% PI 0.2–3.4 million] (Table 2).

**Table 2:** Estimated mean costs and health outcomes averted by RSV interventions (in thousands)

RSV-associated (‘000) mean [95% credible interval]	No intervention		mAb (6 months protection)	Maternal vaccine (5 months protection)
	Under 1 year	1–4 years	Under 1 year	Under 1 year
RSV cases (non-hospital + hospital cases)	7649 [5366–10,138]	13,192 [10,691–15,486]	5928 [4363–7503]	6464 [4712–8231]
Hospital admissions	668 [142–1853]	1150 [258–3139]	517 [112–1421]	564 [122–1555]
Deaths	18 [3–55]	22 [4–68]	13 [2–40]	15 [3–44]
Discounted YLDs	16 [10–24]	26 [19–36]	12 [8–18]	13 [9–19]
Discounted YLLs	517 [89–1561]	593 [103–1832]	383 [68–1139]	421 [74–1257]
Discounted DALYs	532 [102–1581]	619 [124–1865]	395 [77–1155]	434 [85–1276]
Intervention costs (including delivery)	NA	NA	430,531 [430,531–430,531]	220,775 [220,775–220,775]
Discounted outpatient costs	157,114 [69,436–341,707]	256,189 [121,141–545,592]	122,214 [55,209–264,714]	133,078 [59,878–288,301]
Discounted hospital costs	75,254 [15,043–213,053]	122,362 [25,518–343,347]	58,975 [12,080–166,009]	64,033 [13,123–179,855]
Discounted total costs	232,368 [112,538–443,860]	378,551 [194,696–696,863]	611,720 [521,121–770,475]	417,885 [319,308–592,027]
Burden averted				
RSV cases averted	NA	NA	1721 [979–2659]	1186 [641–1924]
Hospital admissions averted	NA	NA	151 [29–443]	104 [19–309]
Deaths averted	NA	NA	5 [1–15]	3 [1–11]
Discounted DALYs averted	NA	NA	137 [23–423]	98 [16–308]
Net discounted costs	NA	NA	379,352 [322,875–408,824]	185,517 [144,452–206,446]

Estimated mean [95% credible interval] of undiscounted number (in thousands) of RSV cases, hospital admissions, and deaths and discounted costs and health outcomes (in thousands) pre- and post-RSV interventions summed over 72 Gavi countries in 2022

### Effectiveness and cost-effectiveness

Maternal vaccination would prevent 1.2 million cases [95% PI 0.6–1.9 million], 104 thousand hospital admissions [95% PI 19–309 thousand], and 3 thousand deaths [95% PI 1–11 thousand] in those countries. It can avert 98 thousand discounted DALYs [95% PI 16–308 thousand] and 186 million USD [95% PI 144–206 million]. The mAb strategy would prevent more cases and avert more discounted DALYs and treatment costs (Fig. 2b, c and Table 2). However, the mAb strategy would also result in higher discounted net costs compared to the maternal strategy due to the assumed higher intervention costs (6 USD vs. 3 USD). Country-specific results are presented in Additional file 1.

**Fig. 3:**
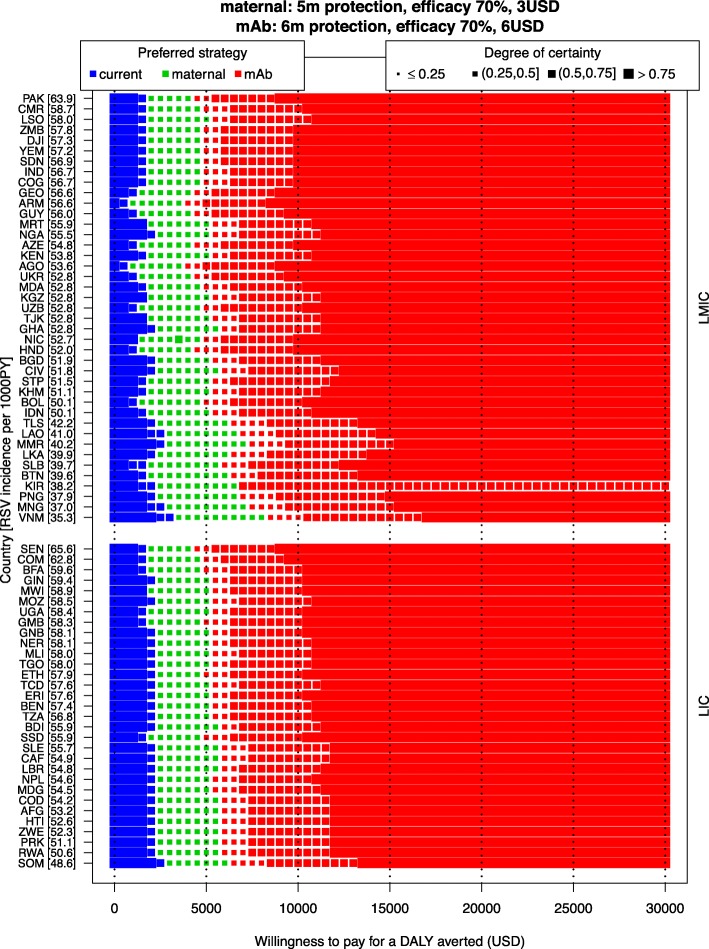
Cost-effectiveness analysis of three interventions to prevent RSV prophylaxis. The optimal strategy in terms of cost-effectiveness for each country and for a range of WTP values (0–30,000 USD per DALY averted) is indicated by the colours (the current strategy is no intervention). The size of the markers indicates how certain we are about that strategy being optimal. Countries are ranked by RSV incidence rate (ordered from high to low on the left *Y*-axis [incidence per 1000 person-year in brackets]) and stratified by income group (LIC or LMIC, on the right *Y*-axis)

Figure 3 presents the same information that is usually presented by a cost-effectiveness acceptability frontier (optimal strategy) and cost-effectiveness acceptability curves (degree of uncertainty), but these presentation formats do not allow to show several countries at once.

It demonstrates that as WTP per DALY averted increases, the maternal and mAb strategies become the optimal strategy for an increasing number of countries. The maternal strategy would become the most cost-effective strategy when the WTP value exceeds 1000 USD at the lower end (Armenia and Angola) or 3000 USD per DALY averted (Vietnam) at the upper end of the country spectrum. The maternal strategy is the most cost-effective strategy over a slightly larger range of WTP values in LMICs (1000–8000 USD) than in LICs (2000–6000 USD), but the between-country differences within each income group are greater than the differences between the two income groups. Similarly, the mAb strategy would be the optimal strategy if the WTP value exceeds 3500 USD at the lower end (Armenia and Angola) or 8000 USD per DALY averted (Vietnam) at the higher end of the country spectrum.

The degree of certainty around the optimal strategy is lower than 50% for the majority of countries, for WTP values between 2500 USD and 7000 USD per DALY averted. That is, for this range of WTP values, the probability that the optimal strategy results in the highest net benefit is less than 50%. Hence, it is worthwhile to explore what causes this substantial uncertainty (next section).

### Uncertainty in the results

#### Expected Value of Partially Perfect Information

**Fig. 4:**
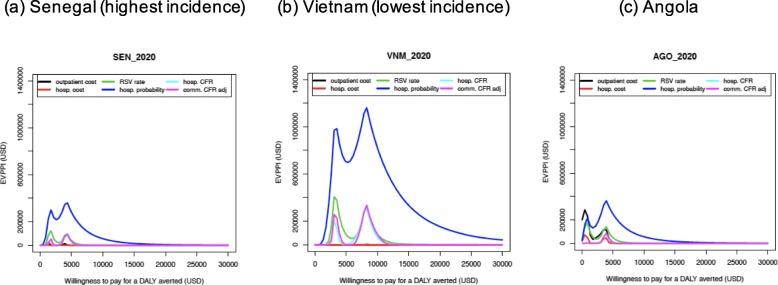
Expected Value of Partially Perfect Information, example of **a** Senegal, **b** Vietnam, and **c** Angola

For each country, the EVPPI was calculated for each uncertain input parameter and for a range of WTP values. For the input parameters with high EVPPI values, it would be (most) valuable to obtain more information in order to identify the optimal strategy with more certainty. Senegal, Vietnam, and Angola are shown in Fig. 4 as examples and can be taken to represent the main differences between countries. Generally, two peaks are observed in the EVPPI graphs for all countries, which represent the WTP value at which the optimal strategy changes from no intervention (current practice) to the maternal, and from the maternal to the mAb strategy. At this WTP value, the optimal strategy is most uncertain, and consequently, obtaining more information is most valuable (i.e. the EVPPI values are the highest). However, the precise WTP values at which the EVPPI values peak, and the size of the EVPPI values vary between countries.

For all countries, and for most of the WTP values considered, the uncertainty in the age-specific RSV hospitalisation probability causes the highest uncertainty in the choice of the optimal strategy. Additionally, the uncertainty around RSV incidence rate, hospital case-fatality ratio, and community case-fatality ratio is also among the top influential factors for the choice of the optimal strategy at all WTP values. A few countries are similar to Angola, where the relevance of other parameters and the ranking of importance depend on the WTP level. In Angola, the uncertainty in the unit costs of outpatient care is a stronger driver of uncertainty than hospitalisation probability at WTP values below 1000 USD per DALY averted, because Angola has a higher outpatient cost with a wider range of uncertainty compared to other countries. In most other countries, uncertainty around the average hospitalisation costs and outpatient consultation costs does not influence the optimal strategy to a great extent (see Additional file 1).

#### Scenario analysis: price, efficacy, and duration of protection

**Fig. 5:**
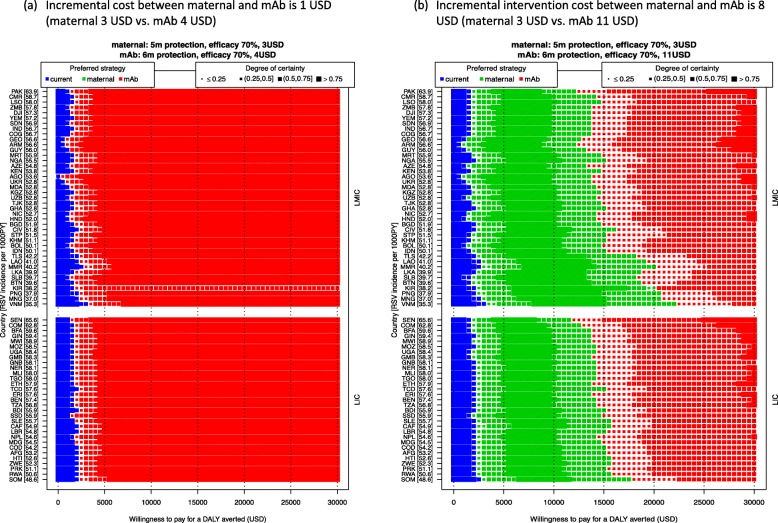
Scenario analysis: the per-dose price difference between interventions and its impact on the optimal strategy. Scenario assuming the incremental cost per dose between the maternal and mAb strategy **a** is 1 USD and **b** is 8 USD. The optimal strategy is indicated by the colours (the current strategy is no intervention), and our certainty is indicated by the sizes of the markers. Countries are ranked by RSV incidence rate (ordered from high to low on the left Y-axis [incidence per 1000 person-year in brackets]) and stratified by income group (LIC or LMIC, on the right Y-axis)

Price scenario analysis demonstrates that when the incremental intervention cost per dose between maternal and mAb strategy is 1 USD (3 vs. 4 USD), the mAb is the optimal strategy (Fig. 5a). In other words, an extra month of protection is worth more than 1 USD. When the incremental intervention cost increases to 8 USD (3 vs. 11 USD), the mAb only becomes optimal at WTP values that exceed 15,000 USD per DALY averted.

**Fig. 6:**
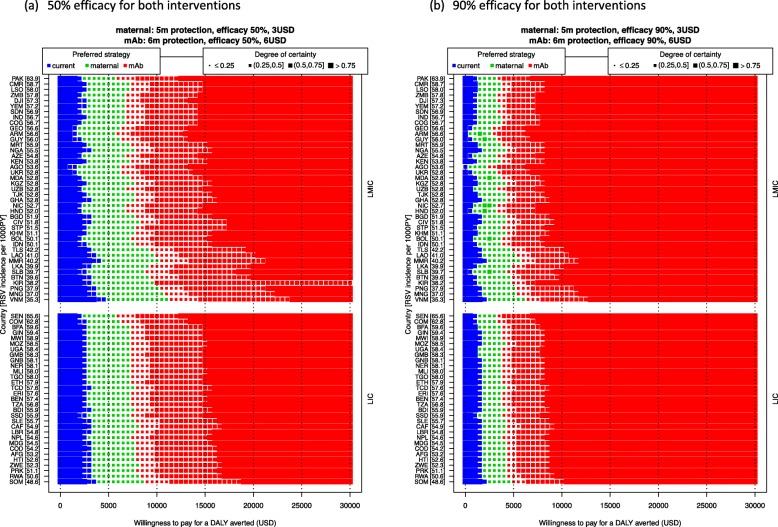
Scenario analysis: the efficacy of the interventions and its impact on the optimal strategy. The optimal strategy is indicated by the colours (the current strategy is no intervention), and our certainty is indicated by the sizes of the markers. Countries are ranked by RSV incidence rate (ordered from high to low on the left *Y*-axis [incidence per 1000 person-year in brackets]) and stratified by income group (LIC or LMIC, on the right *Y*-axis)

Scenario analysis on the intervention’s efficacy shows that if the efficacy of both interventions is 50%, the maternal and mAb strategies require a WTP value of 3000–5000 USD and 5500–11,500 USD, respectively, to become the optimal strategy. However, if the efficacy of both interventions is 90%, the switches from the no intervention to maternal strategy occur at a WTP of 500–2500 USD, and from the maternal to mAb strategy at 2500–6000 USD. That is, these changepoint WTP values decline when efficacy increases, and vice versa (Fig. 6).

**Fig. 7:**
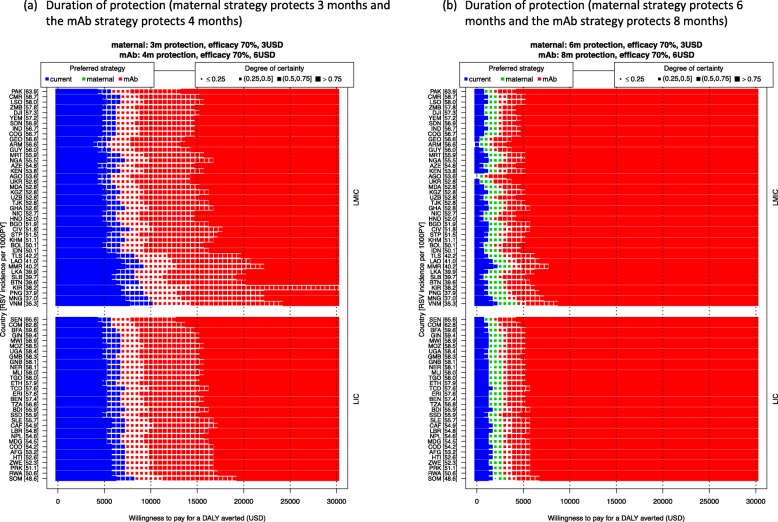
Scenario analysis: the protection duration of the interventions and its impact on the optimal strategy. The optimal strategy is indicated by the colours (the current strategy is no intervention), and uncertainty is indicated by the sizes of the markers. Countries are ranked by RSV incidence rate (ordered from high to low on the left *Y*-axis [incidence per 1000 person-year in brackets]) and stratified by income group (LIC or LMIC, on the right *Y*-axis)

The uncertainties on interventions’ duration of protection are evaluated in Fig. 7. When the maternal and mAb strategies only protect for 3 months and 4 months, respectively, neither of these become the optimal strategy for a WTP value above 5000 USD per DALY averted and the maternal strategy is unlikely to be the optimal strategy at any WTP value (Fig. 7a). When maternal and mAb strategies offer protection of 6 and 8 months, respectively, the maternal strategy has a probability of being the optimal strategy at WTP values of 1000 to 4000 USD, and the mAb strategy gradually becomes the optimal strategy when the WTP value is above 5000 USD.

The results of scenario analyses using the pooled hospital probability (Nokes et al. [22] and Homaira et al. [21]) estimates, using no discounting and trial-based efficacy, are presented in Additional file 1.

## Discussion

Health economic evaluations for multiple interventions and multiple countries are invaluable for decision-makers involved in global initiatives. Our model application, MCMARCEL, has been designed specifically to simultaneously and efficiently evaluate the costs and effects of many countries and strategies, fully accounting for parametric uncertainty, with the aim to identify influential parameters. This study evaluated the health and economic effects of potential maternal RSV vaccines and monoclonal antibodies for infants in 72 current and former Gavi-eligible countries. We estimated that the annual disease burden in these 72 countries is substantial, including 20.8 million RSV cases, 1.8 million hospital cases, and 40 thousand deaths, with average total direct costs exceeding 600 million USD. We found that the maternal strategy would be the optimal strategy given a price of 3 USD per dose at a WTP value greater than 3000 USD in most countries (range 1000–3500 USD) per DALY averted, whereas the mAb strategy would be optimal given a price of 6 USD per dose at a WTP value greater than 6000 USD (range 3500–8000 USD) per DALY averted. At these WTP values, the results are surrounded by large uncertainties mainly caused by uncertainty around RSV incidence, mortality, and especially RSV hospitalisation rates. At lower WTP values (below 2500 USD per DALY averted), we are more than 75% certain that no intervention is optimal for the majority of countries. Hence, despite the notable burden of RSV in early life, the short-lived protection of the potential tools indicates that any prophylaxis would have to be competitively priced in order to be considered cost-effective in Gavi-eligible settings.

To date, there is no licensed RSV vaccine (for mothers or children) nor single-dose mAb, and the interventions’ duration of protection is unknown. An additional month of protection was assumed for the mAb versus the maternal vaccination strategy, which is consistent with the assumptions in the Gavi RSV investment case [31]. If mAb would offer the same efficacy with a shorter duration of protection, but at a higher price than the maternal vaccine, then mAb would simply be dominated by the maternal vaccination strategy, irrespective of willingness to pay. For both the maternal vaccination and mAb strategies, simplified ‘all or nothing’ assumptions on duration of protection were made, where we assumed no waning of protection up to the end of the period of protection, at which point protection drops to zero. We also assume the same duration of illness for both non-vaccinated and vaccinated children. The reason for such assumptions is that the duration of protection is unknown. Hence, sensitivity analyses were performed (Additional file 1). In this analysis, the focus is on estimating the disease burden, given the currently available data, and in identifying the main data gaps to fill (besides information on the intervention tools) in order to reduce decision uncertainty about interventions (defined according to simple characteristics). Given a modelled period of protection, this simplification—without waning immunity—is likely to result in an overestimation of disease averted from these interventions, ceteris paribus.

The most advanced candidate is the maternal RSV vaccine (NCT02624947) developed by Novavax. Unfortunately, its phase 3 randomised controlled trial did not demonstrate efficacy against its primary endpoint (medically significant RSV LRTI), but it showed significant efficacy against one of its secondary endpoints (hospitalisation) [6]. In order to maximise the information provided by our simulations, we not only modelled an extensive range of scenarios based on efficacy intervention and price ranges (while accounting for all parameterised uncertainty), but we also modelled a scenario using currently available peer-reviewed data on the Novavax maternal vaccine (while accounting for all parameterised uncertainty, including wide uncertainty intervals for efficacy against the primary and secondary endpoints; see Additional file 1) [6]. However, additional phase 3 trial data of this vaccine were presented at a recent international conference [32]. These new per-protocol post hoc analyses were interpreted by Novavax as indicating their maternal vaccine is effective against the broad endpoint of all-cause pneumonia (i.e. not just RSV LRTI) among infants. Clearly, if the RSV interventions (maternal vaccine or mAb) would offer such additional protection, its value would greatly increase. This remains as yet a controversial result—especially since the same trial failed to demonstrate significant efficacy against its FDA-prescribed primary endpoint—that needs to be confirmed by thorough peer review. In view of potential confounders influencing the broad endpoint of ‘any medically significant LRTI’, it is important to establish at least whether (a) randomisation accounted for comparable within-household and community vaccination status against influenza, pneumococcus, and *Haemophilus influenzae* type b in both arms of the trial; (b) these observations hold in the different country sites of the trial; and (c) to which extent this is observed for outpatient versus inpatients. We therefore opted not to explore the ramifications of these potential benefits in additional scenario analyses in the current paper. Nevertheless, our extensive sensitivity analyses on efficacy, waning, and price improve our understanding of the cost-effectiveness of new RSV interventions. Therefore, this type of analysis can inform vaccine developers’ product portfolio development, as well as decision-makers and their advisors’ planning activities.

When WTP values are less than 1000 USD per DALY averted, there is little uncertainty, given the product characteristics we explored, that current practice is optimal, and the resources are better spent on other interventions in these countries. If WTP values above 1000 USD and 3500 USD are considered acceptable, the maternal and mAB strategies, respectively, are increasingly likely to become the optimal strategies. This last result is however surrounded by much uncertainty. Hence, it would be valuable to obtain more evidence on RSV incidence, mortality rate, and especially hospitalisation probability. Of course, a refined understanding of the features of any RSV prophylaxis would go a long way in reducing the uncertainty around the optimal intervention. However, community-based incidence studies, even those undertaken prior to the implementation of RSV prophylaxis, could also play a role in reducing the uncertainty around the question of cost-effectiveness. According to our analysis, the importance of community-based incidence studies, as opposed to hospital-based incidence studies, cannot be stressed enough; the probability of cases in the community that are hospitalised is one of the most valuable parameters that we could study, and this parameter cannot be estimated from any of the 76 hospital-based incidence studies that are documented in Shi et al. (as they lack any information on the incidence burdening the community in which the studies were set) [2]. Moreover, the hospital-based case-fatality ratios cannot reflect the overall RSV-associated deaths in LIC and LMIC settings. We calculated the overall RSV-associated death rates based on adjustment factors derived in Shi et al. from only three studies in LMIC, as no country-specific data is available. The adjustment factor of 2.2 (with uncertainty interval of 1.5–2.9) was used for all countries to country-specific hospital case-fatality rates in order to estimate country-specific RSV community deaths. In countries where relatively fewer RSV-associated ALRI cases seek health care, this factor is likely to be higher and vice versa. It is therefore highly likely that community RSV deaths were underestimated for LIC. In our EVPPI analysis, this adjustment factor is one of the top influential factors for this analysis. Hence, in addition to the importance of the burden information per se for policy-makers, studies on RSV-associated community deaths can also provide highly useful information for cost-effectiveness analysis.

To the best of our knowledge, this is the first multi-country, multi-intervention cost-effectiveness analysis for childhood prophylactic interventions against RSV in LIC and LMIC settings. Two modelling studies applied Kenyan data to understand the impact of RSV vaccination in low-income settings. Poletti et al. evaluated several prevention strategies, including maternal, paediatric (at 3 months), and school-age vaccination. They predicted a 30% reduction in RSV infant infection via maternal vaccination, which is very similar to our prediction [26]. Additionally, Kinyanjui et al. used a dynamic transmission model to investigate the health impact of vaccinating older children when natural maternal immunity has waned (5–10 months) on infants, which is a different research question [33].

This analysis has several other strengths: firstly, we re-estimated the age-specific disease incidence on a per-month basis using detailed data presented in a published systematic review and we used model-based estimates of the total country-specific burden in all 72 countries. Next, rather than estimating one region-level cost of illness [34], we estimated country-specific costs using a simple method that leveraged the existing literature on pneumonia costs in combination with the WHO-CHOICE costs to capture the country-to-country variation in costs. Secondly, we accounted for parameter uncertainty in a probabilistic way and performed extensive scenario analyses to assess the impact of uncertainty in parameters that have multiple and complex effects on the outcomes and costs of RSV. We also plotted the overall trends and impact of different assumptions in both current disease trends and potential implementation programmes. Thirdly, this analysis includes a value of perfect information analysis to inform data collection prioritisation both for countries and vaccine developers in order to improve the timeliness of the decision process once the products become available. Moreover, it also reflects the ‘full value of vaccines’ agenda from the WHO that encourages evaluation of vaccine value during the development phase so as to avoid vaccine manufacturers bring unmarketable vaccines into late stage development or onto the market [35]. Finally, the investment decisions of RSV interventions need to be made both at the country and multi-country level (i.e. through the WHO policy recommendations and Gavi financial support). Since the WHO has explicitly discouraged the use of GDP per capita as a benchmark of the incremental direct costs that a country should be willing to pay to gain a QALY, or avert a DALY [36], we present and interpret this complex analysis across different WTP levels for various stakeholders. Consequently, the decision of the optimal strategy can be made based on any WTP threshold within the wide range we explored.

Within each country, the coverage is likely to differ between different intervention strategies, whereas we assumed BCG infant coverage for all strategies in the interest of parsimony. Since this is a static model and marginal intervention costs are assumed to be directly proportional to doses administered, the coverage will only impact the overall disease burden averted and not the cost-effectiveness [37]. Although the maternal tetanus vaccination programme is available in a few countries under the tetanus elimination goal, the maternal vaccination programme is not yet well established in most of the 72 countries, representing an implementation challenge. The maternal vaccine could be administered during an ANC visit. The current WHO recommendation for maternal acellular pertussis vaccination is in the second or third trimester and at least 15 days before birth [38]. With the ANC visit coverage and frequency (ranging from 4.3% in Somalia to 96% in Armenia, at least four times throughout pregnancy) in LIC and LMIC countries [10], maternal immunisation coverage is unlikely to achieve the BCG coverage level, implying we may have overestimated the impact of this strategy on disease burden.

The maternal strategy captures only the health benefit to infants in our model; however, the potential benefits to the mothers and household contacts are not considered, as the burden of RSV in childbearing-age females and the RSV vaccine duration of protection in adults are both unknown. From the literature, the highest disease burden of RSV is in the very young age group; therefore, the health benefits to the mothers or contacts are unlikely to have a significant impact on the overall results. Additionally, there may be uncaptured psychosocial benefits to caregivers in improved child survival and health. Future research could consider factors in RSV epidemiology that were beyond the scope of the current model’s structure and the data available. The impact of transmission dynamics could capture the effects of prophylaxis on the contacts of the mother or the child, but the existent data on disease dynamics precludes the development of a dynamic model applicable to all settings in our analysis. Relatedly, our model assumes that cases averted on a certain month are averted altogether, rather than shifted to later ages, potentially favouring the case for RSV prophylaxis. However, RSV incidence peaks at a very young age in most countries and younger cases are more severe than older cases (in terms of the need for hospitalisation and the probability of death); hence, we believe that a slight increase in incidence in children over 6 months of age would not represent a displacement in time of hospitalised/fatal cases, since older cases are less likely to result in hospitalisation or death.

The seasonality of RSV is not considered in this evaluation as the analysis includes countries in several continents with differing seasonal patterns. It is challenging to identify the start and peak of RSV seasons in the absence of good surveillance in these countries. Cromer and colleagues explored a seasonal RSV vaccination programme in the UK, showing that targeting births in particular months of the year might be more efficient than a year-round programme [12]. The implementation challenges of such a seasonal programme in low- and middle-income countries are currently not well documented. For instance, young infants born before the RSV season would still be vulnerable for severe RSV disease, but they may be difficult to reach with a seasonal programme. One way to interpret our results in light of a seasonal programme is by assuming that this would require fewer doses for the same (or similar) effectiveness. For instance, if only half of pregnant women/newborns would need to be targeted to protect them through an RSV season occurring over part of the year, then all WTP changepoints (where the optimal strategy of choice changes) in our analyses would be approximately halved.

This analysis also has a few additional limitations, but all render our analysis more conservative (we bias against implementation and the potential misuse of existing resources). The indirect effects on disease transmission are ignored, which in the case of the strategies considered here would likely represent an underestimation of the benefits. The fixed implementation costs (i.e. campaign, establishing a new vaccination visit instead of using existing platform) were not considered, although the intervention cost (procurement and delivery costs) per dose was included as a variable in our model. If one strategy would require higher fixed costs than another, it may impact the ICERs and therefore the choice of the optimal strategy. Direct non-medical costs (e.g. transportation) and indirect costs (e.g. productivity losses) are not included in our analyses due to a lack of data. Uncertainty around the proportion of RSV cases not seeking health care was not explored due to insufficient information. Interventions targeting high-risk groups only (i.e. preterm, bronchopulmonary dysplasia, and chronic heart disease) are not considered as these groups are unlikely to be identified easily in these countries. This analysis focuses on the RSV-associated ALRI as defined in the systematic reviews [1, 2]. Other RSV-associated acute infections are not included (i.e. upper respiratory tract infection, otitis media). Longer-term chronic conditions, such as recurrent wheezing and asthma, are also not considered yet due to limited evidence that these are influenced directly by RSV infections; there is also a paucity of health and cost burden data on these conditions for the countries considered here. Furthermore, a recent study showed that current RSV prophylaxis has a limited impact on the reduction of these conditions [39]. Evidence about the causal association between RSV and these conditions is still emerging.

## Conclusion

RSV interventions could prevent substantial RSV-associated cases, hospital admission, and deaths, consequently averting sizable DALYs and treatment costs. However, if the extent and duration of effectiveness of maternal vaccine and monoclonal antibody RSV strategies turn out in line with our assumptions, these interventions may become optimal compared with current practice only at high willingness to pay values per DALY averted and/or competitive per-dose prices. In order to assess their relative cost-effectiveness, information on the nature, level, and duration of protection these intervention options confer would be required. The current analysis indicated that more empirical research on the proportion of all RSV cases that are hospitalised, RSV incidence, and case-fatality ratios in hospitals and the community would be highly valuable to reduce decision uncertainty when detailed clinical trial data eventually become available.

## Supplementary information

**Additional file 1.** Additional details on methods and results of sensitivity analyses.

## Data Availability

Almost all the data analysed and generated during this study are included in this published article and its supplementary information files. Additional data are available from the corresponding authors on reasonable request.

## Abbreviations

ALRI: Acute lower respiratory infection
ANC: Antenatal care
BCG vaccine: Bacillus Calmette-Guérin vaccine
CEAC: Cost-effectiveness acceptability curves
CEAF: Cost-effectiveness acceptability frontier
DALY: Disability-adjusted life year
EPI: Expanded Programme on Immunization
EVPPI: Expected Value of Partially Perfect Information
GDP: Gross domestic product
LIC: Low-income country
LMIC: Lower-middle income country
LoS: Length of stay
mAb: Monoclonal antibody
PY: Person-years
RSV: Respiratory syncytial virus
VIS: Vaccine investment strategy
WHO: World Health Organization
WTP: Willingness to pay

## Footnotes

1 Cuba was excluded from the Gavi-eligible countries due to a lack of country-specific data available to use.
