# Supplementary material for: Health and economic burden of respiratory syncytial virus (RSV) disease and the cost-effectiveness of potential interventions against RSV among children under 5 years in 72 Gavi-eligible countries
Source: BMC Med. 2020 Apr 6;18:82. doi: 10.1186/s12916-020-01537-6 (PMC7132892; doi:10.1186/s12916-020-01537-6)
Supplement: Supplementary file 1 — Additional file 1. Additional details on methods and results of sensitivity analyses. [file 12916_2020_1537_MOESM1_ESM.pdf]

Additional file 1:

Title: Health and economic burden of Respiratory Syncytial Virus (RSV) disease and the cost-effectiveness of potential interventions against RSV among children under 5 years in 72 Gavi-eligible countries

Authors: Xiao Li[1], Lander Willem[1], Marina Antillon[1-3], Joke Bilcke[1], Mark Jit[4], Philippe Beutels[1]

[1] Centre for Health Economics Research & Modelling Infectious Diseases, Vaccine & Infectious Disease Institute, Universiteitsplein 1, University of Antwerp, Belgium

[2] Swiss Tropical and Public Health Institute, Socinstrasse 57, Basel Switzerland

[3] University of Basel, Petersplatz 1, Basel Switzerland

[4] Department of Infectious Disease Epidemiology, London school of Hygiene and Tropical Medicine, Keppel St, Bloomsbury, London, United Kingdom

Table of Contents

1 METHODS 3

1.1 DISEASE BURDEN 3
1.2 META-ANALYSIS OF RSV TREATMENT COSTS AND THE ADJUSTMENT FACTORS 6

2 RESULTS 10

2.1 ESTIMATED RSV-ASSOCIATED DISEASE BURDEN AND THE IMPACT OF INTERVENTIONS AGAINST RSV 11
2.2 EXPECTED VALUE OF PARTIALLY PERFECT INFORMATION (EVPPI) 11
2.3 SCENARIO ANALYSIS: USING POOLED HOSPITAL PROBABILITY 18
2.4 SCENARIO ANALYSIS: WITHOUT DISCOUNTING 20
2.5 SCENARIO ANALYSIS: TRIAL-BASED VACCINE EFFICACY 22

3 REFERENCES 27

4 LONG TABLES 28

Tables and Figures

S. Table 1: Estimated mean costs and health outcomes averted by RSV interventions using pooled hospital probability data 18
S. Table 2: Estimated mean costs and health outcomes averted by RSV interventions using 0% discounting rate 20
S. Table 3:Reported vaccine efficacy in phase 3 PrepareTM trial 22
S. Table 4: Vaccine efficacy estimates for the trial-based scenarios 23
S. Table 5: Estimated mean costs and health outcomes (in thousand) in children under 1 using trial-based-efficacy values 24
S. Table 6: Estimated age-specific RSV cases in each country 28
S. Table 7: Average outpatient and inpatient costs of the 72 Gavi countries in USD at 2016 value 30

S. Table 8: Country-specific estimated mean of RSV-associated disease burden and the impact of interventions 33

S. Figure 1: Predictions from spline regressions of probability of death in among hospitalised patients..........5
S. Figure 2: Predictions from spline regressions of community-based incidence, probability of hospitalisation and probability of death 6
S. Figure 3: Meta-analysis, outpatient adjustment ratio (log mean and log standard deviation)..........7
S. Figure 4: Meta-analysis, inpatient adjustment ratio (log mean and log standard deviation)..........8
S. Figure 5: Meta-analysis, outpatient costs where multiple studies reported treatment costs (log mean and log standard deviation)..........9
S. Figure 6: Meta-analysis, hospitalisation costs where multiple studies reported treatment costs (log mean and log standard deviation).10
S. Figure 7: Expected Value of Partially Perfect Information (EVPPI) of all 72 countries..........17
S. Figure 8: Cost-effectiveness analysis when using pooled hospital probability data 19
S. Figure 9: Cost-effectiveness analysis when assuming a 0% discount rate. 21
S. Figure 10: Cost-effectiveness analysis for different scenarios related to the trial-based maternal vaccine efficacy..........26

1 Methods

1.1 Disease burden

Generalized additive mixed models (GAMM) were used to estimate the parameters of an interpolation spline for each of our outcomes of interest (cases, hospitalisations, and deaths among hospitalized individuals). Splines are flexible piece-wise defined functions used to describe and interpolate a response in scenarios when the process that gives rise to the data is ill-described by more traditional linear or log-linear functions. Smoothing splines circumvent the problem of having to select the number of pieces that an interpolant must have by penalizing for overly complicated functions [1].

The splines were built to decompose the variance in the data into two components: a global trend using a fixed-effect spline, and a study-specific trend using random-effect splines that accounts for the difference between the age-related trend in each study and the trends observed globally. Three splines were estimated: I) community-based incidence, II) hospital probability, III) probability of death among hospitalized patients. The flexibility of the GAMM framework allowed us to model the incidence spline (I) assuming a Poisson process, and the probability splines (II and III) assuming a binomial process.

The structure of functions are as follows:

For incidence (assuming a Poisson function):

$$log\ (cases/population) \sim \alpha_0 + \alpha_i + s(log(age)) + s(log(age),\ studyi)$$

Traditionally, the combination of a property of log functions means that the population element is put in the right-hand side as a predictor constrained to have a coefficient equal to one (called an “offset”):

$$log\ (cases) - log(/population) \sim \alpha_0 + \alpha_i + s(log(age)) + s(log(age),\ studyi)$$

$$log\ (cases) \sim \alpha_0 + \alpha_i + s(log(age)) + s(log(age),\ studyi) + offset[log(population)]$$

For probabilities (splines II and III) assuming a binomial process:

$$logit\ (p) \sim \alpha_0 + \alpha_i + s(log(age)) + s(log(age),\ studyi)$$

α_0 is the overall intercept (the probability of hospitalisation or death at age 0)

α_i study-specific random effect on the intercept.

s(log(age)) a global trend (fixed-effect) spline for the correlation between the outcome and age, constructed with a thin-plate spline.

s(log(age), $study_i$) a set of random-effects splines constructed with a tensor product basis that used a combination of a thin-plate spline and a random-effects basis to construct splines.

Age groups were mapped onto the number line by assigning the midpoint of the age group as the age that would be put into the model (i.e., observations of a group of 0-3 month-old children were considered to be the observations of children 1.5 month-old children). The age values were log-transformed because the data exists at a higher resolution in the younger ages than in the older ages, and this would allow us to estimate splines with knots that are approximately equally spaced. We discarded studies that had fewer than three studies, as no age-curve would be possible other than a straight line.

The shape of the curve is determined by two components: 1) the number of “knots”, or places when the curve changes direction or convexity, and 2) the “smoothing” parameter, which constrains the number of grooves and inflections that a curve may have. The smoothing parameter is fit as part of a penalty term on the objective function. The penalty term is given by the second derivative of the model, thus penalizing for more intricate curves. Thin-plate splines were used in order to avoid selecting a basis (b-spline, cubic spline). The penalty term in the smoothing splines circumvents the need to find the ideal number or location of knots in each regression model. The selection of the smoothing parameter of the penalty term is done through k-fold cross-validation (in which one or more data-points are left out to estimate parameters and the resulting model is scored for its capacity to predict the data points that are left out).

We used a “shrinkage” variant of the thin-plate spline, which “shrinks” the spline towards a straight line; in other words, the algorithm encourages shapes that are as close to a straight line as is sensible unless there is strong evidence that an alternative shape is warranted, thus avoiding overly complex

curves to describe such diverse settings as the GAVI 72 countries. This was especially important for the probability of death splines, which were sometimes prone to unusual shapes to capture the few deaths that were observed in the data.

A tensor product basis was used to construct the random-effect splines specific to each study. Such a spline was the interaction of thin-plate splines and random-effect parametric terms that were assumed to be normally distributed (a ridge penalty).

S.Figure 1 demonstrates the prediction splines for hospital case fatality rate (hCFR). The light pink bands show the splines derived from GAM models for LIC and LMIC. The blue bands present pooled splines from both LIC and LMIC. The solid lines represent the expected mean of the setting-specific splines. We used the splines of LMIC settings in the main analysis, because of the more explicit age-specific pattern among infants under 6 months, in line with the literature [2, 3].

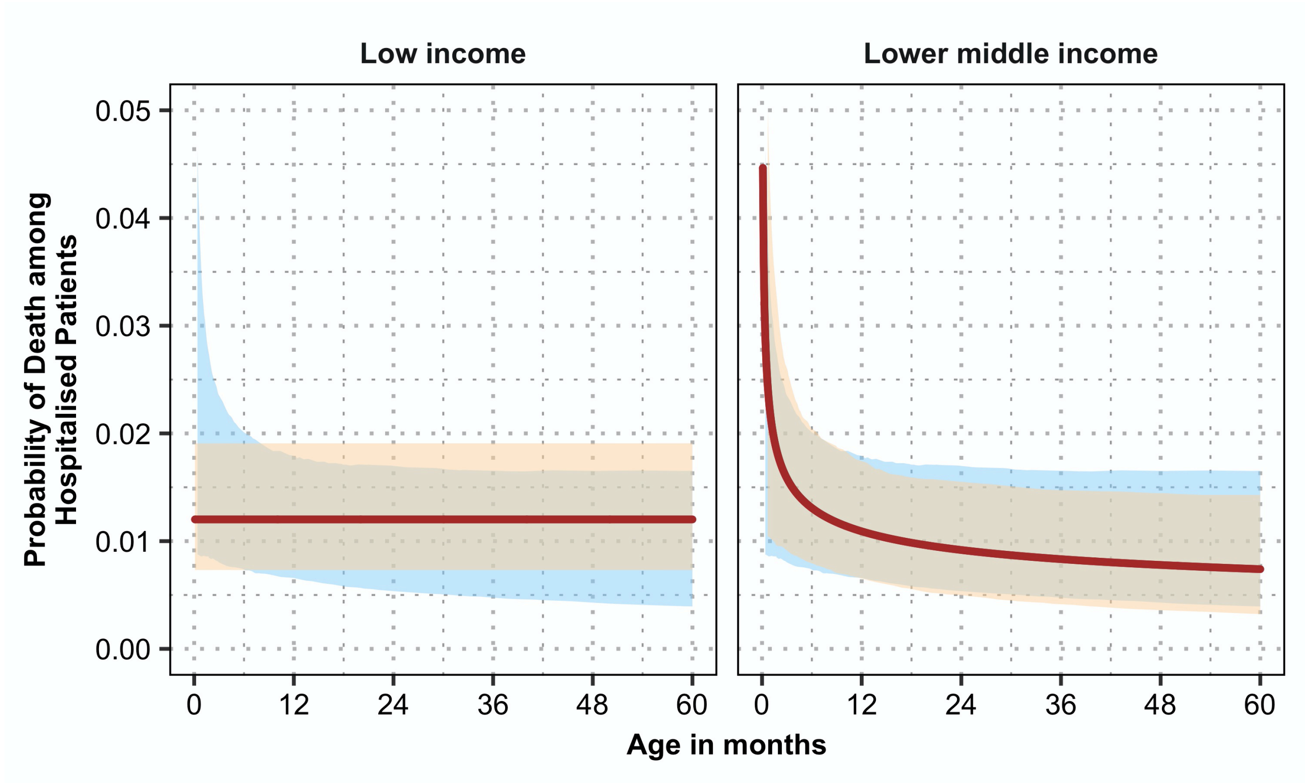

S. Figure 1: Predictions from spline regressions of probability of death in among hospitalised patients

The bands correspond to the 95% confidence intervals of hospital case fatality ratio at each age. Probability of death using data from LIC and LMIC (in yellow) and the pooled analysis were shown (in blue). The solid red line presents the expected mean. The pink band in the LMIC was used in the model for the main analysis.

S. Figure 2 shows the three prediction splines. The pink bands present the splines that derived from GAM models. The blue bands present "Nokes 2008 only" splines in the S. Figure 2 (b) [4]. The solid lines represent the expected mean at each age.

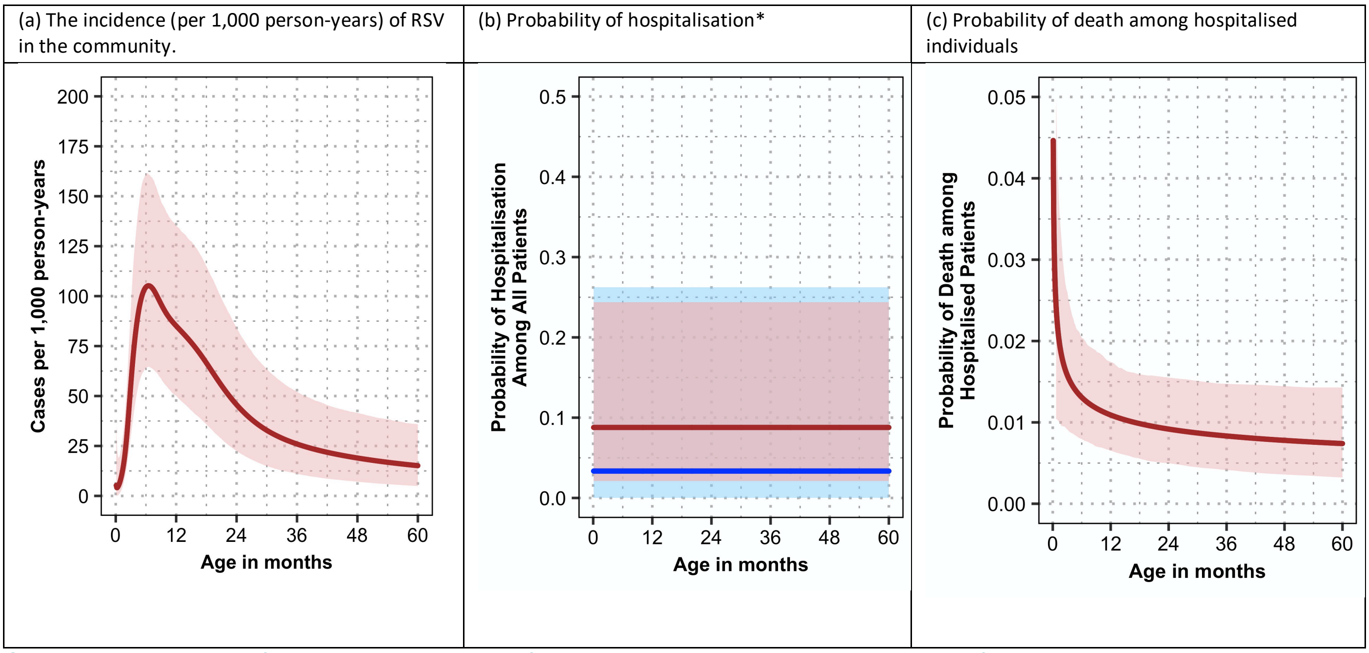

S. Figure 2: Predictions from spline regressions of community-based incidence, probability of hospitalisation and probability of death

The bands correspond to the 95% confidence intervals of each parameter at each age. * Probability of hospitalisation using data from one LMIC where hospitalisations (in pink) and from two LMICs:Bangladesh and Kenya [4, 5] (in blue) were actually observed. The pink band was from the model used for the main analysis and the blue band was from the model used for the supplemental analysis.

Long tables S. Table 6 listed the mean of estimated age-specific RSV cases in each country.

1.2 Meta-analysis of RSV treatment costs and the adjustment factors

Firstly, based on the WHO-CHOICE estimates, gamma distributions were assigned for both outpatient costs (H1 hospital) per visit and inpatient costs (H2 hospital) per day, where we assume $\alpha=\mu2/s2$; $\beta= s2/\mu$ following the method of moments (μ is the mean and s is the standard error, not the standard deviation, of the cost) [6, 7]. Then the country-specific length of stay (LoS) data were retrieved from the systematic review and multiplied with the inpatient cost per day [8]. If there is no country-specific LoS data available, the global mean LoS of 5.8 (95% CI 5.3-6.4) days was applied. Gamma distributions were fitted and 5,000 simulations were drawn.

Secondly, pneumonia treatment costs (used as a proxy for RSV associated ALRI) were extracted from the systematic review [8] and a gamma distribution was also applied following the same rules as above. Although the published studies were reported from different perspectives (i.e. health care payer, household, societal), only the reported direct treatment costs were used in the meta-analysis. The outpatients (OP) and inpatients (H) ratios were calculated as shown below:

OP ratio=systematic review outpatient costs / WHO-CHOICE outpatient estimates

*H ratio=systematic review inpatient costs / (WHO-CHOICE inpatient estimate per day * LOS)*

Thirdly, meta-analyses for both ratios were performed and the results were presented in S. Figure 3 and S. Figure 4. The overall OP adjustment ratio was 21.8 (95% CI 8.0-59.15) and H adjustment ratio was 3.1 (95% CI 2.1-4.7). Therefore, for countries where no country-specific data were identified in the systematic review, the WHO-CHOICE outpatient and inpatients costs were adjusted using these adjustment factors.

Finally, for the countries where multiple studies were identified in the review, meta-analyses were performed to calculate a single treatment cost estimate for the county (S. Figure 5 and S. Figure 6). Both inpatient and outpatient costs in the 72 countries were listed in S. Table 7.

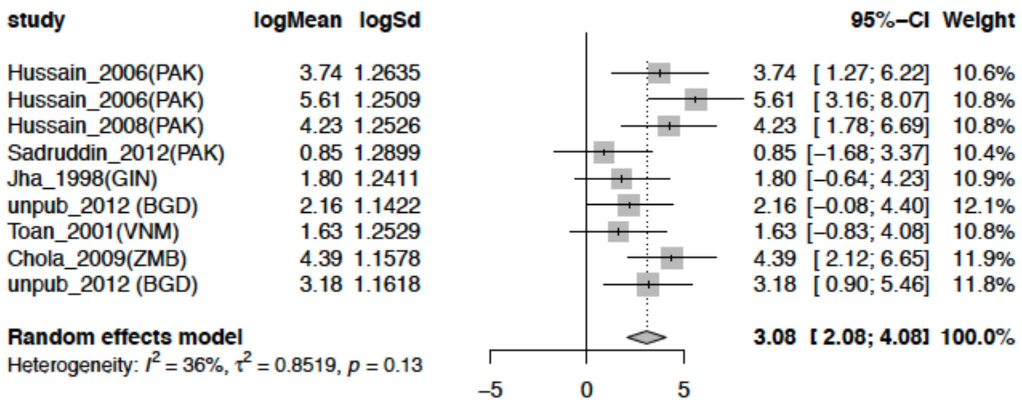

S. Figure 3: Meta-analysis, outpatient adjustment ratio (log mean and log standard deviation)

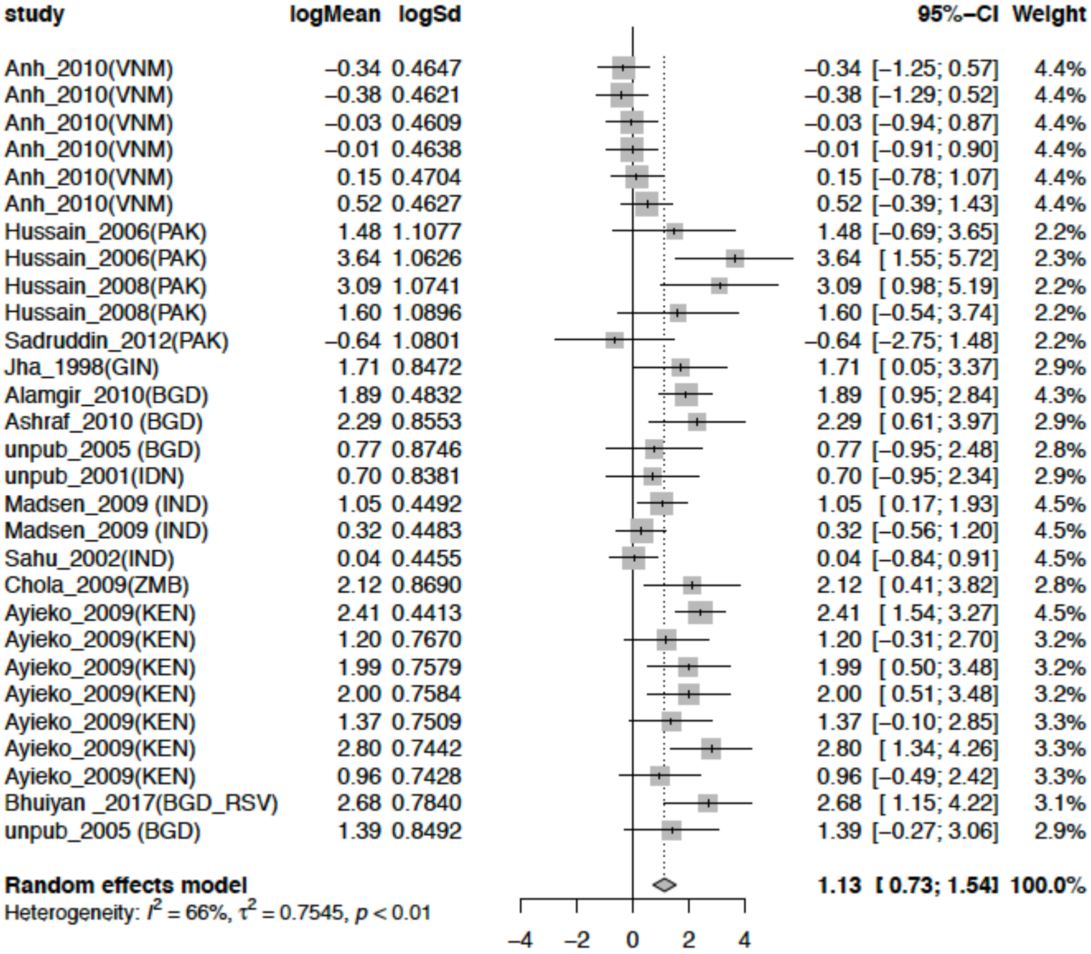

S. Figure 4: Meta-analysis, inpatient adjustment ratio (log mean and log standard deviation)

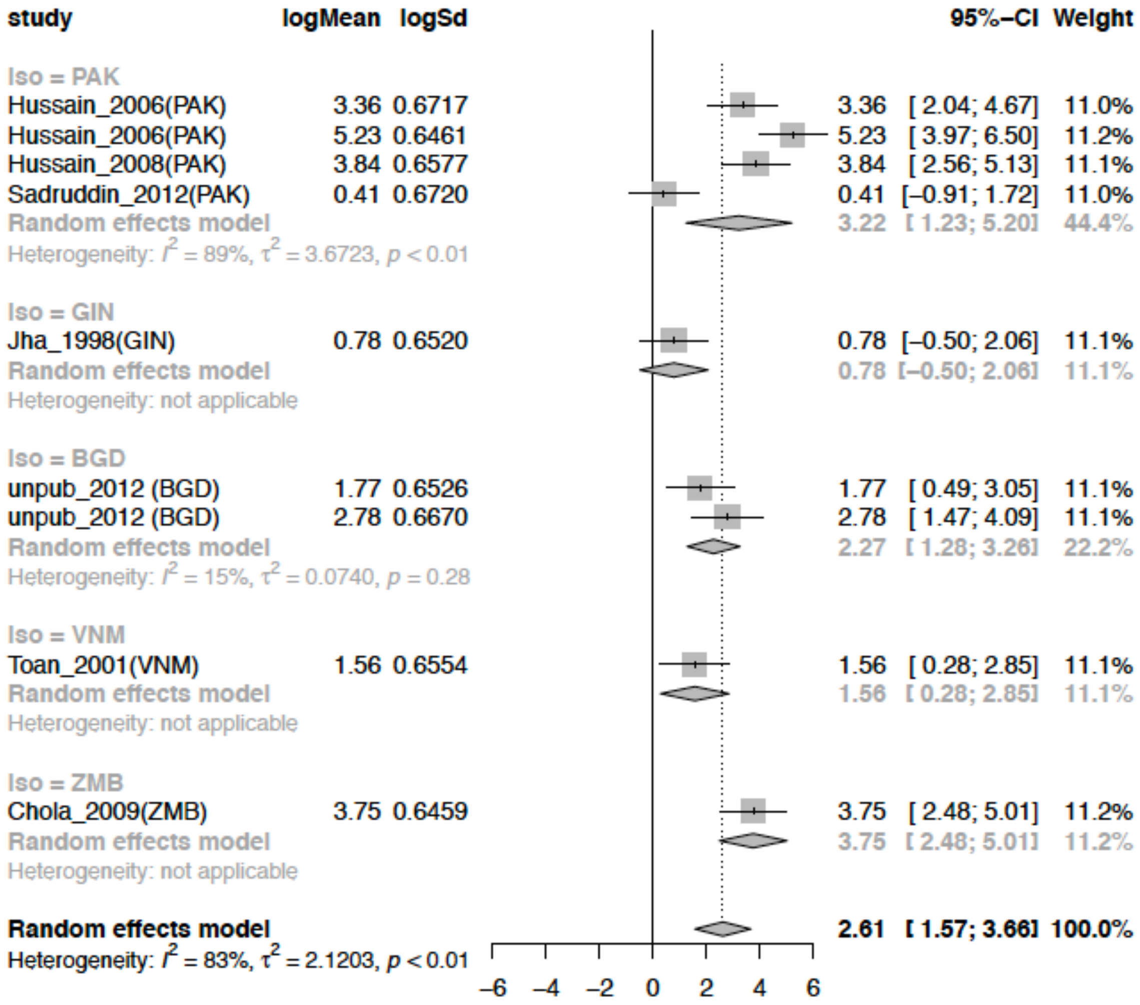

S. Figure 5: Meta-analysis, outpatient costs where multiple studies reported treatment costs (log mean and log standard deviation)

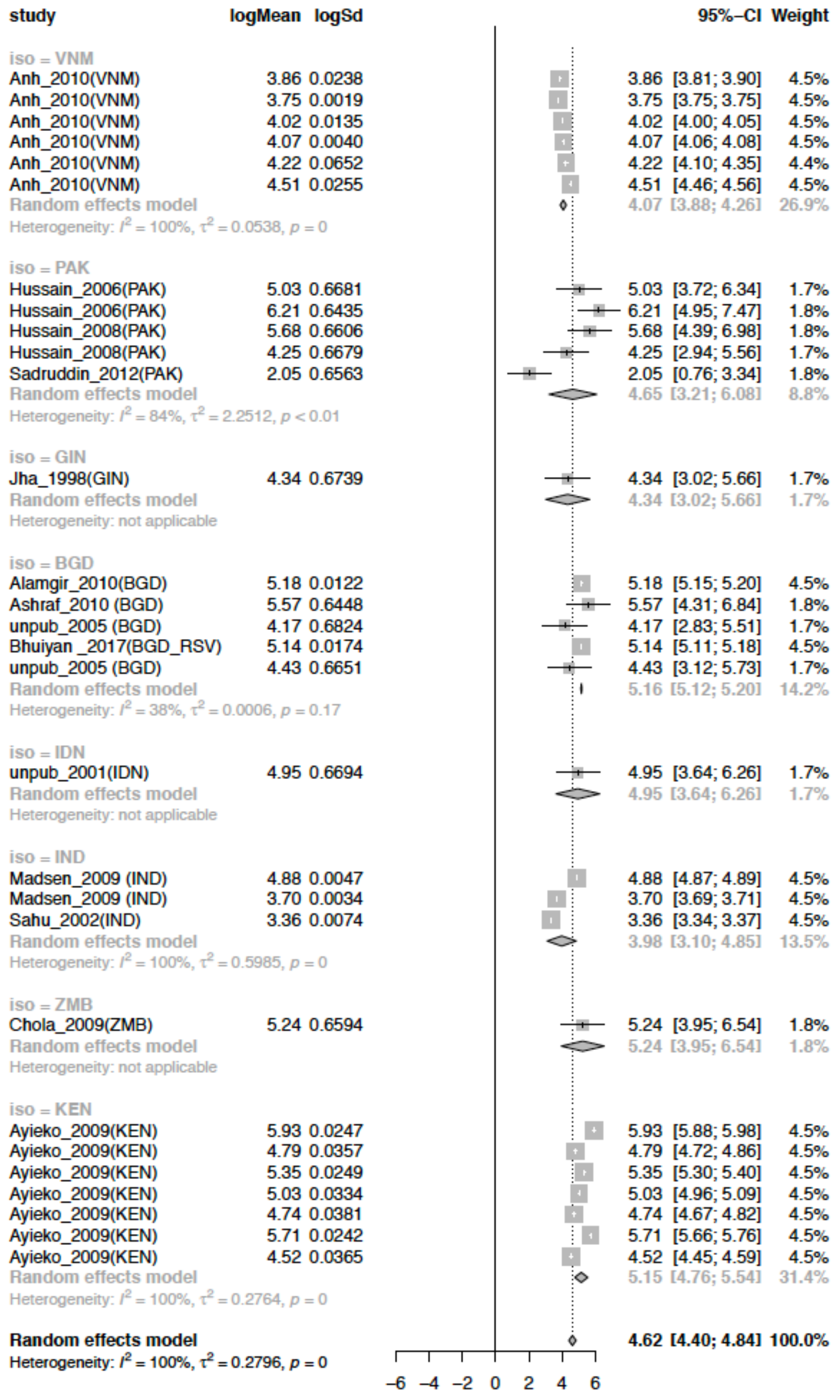

S. Figure 6: Meta-analysis, hospitalisation costs where multiple studies reported treatment costs (log mean and log standard deviation)

2 Results

Country-specific burden of disease estimates and scenario analyses results are reported in this section.

2.1 Estimated RSV-associated disease burden and the impact of interventions against RSV

Table 2 in the main text (section disease burden) reports the aggregated RSV-associated disease burden pre-and post RSV intervention over 72 Gavi countries. S. Table 8 lists the country-specific results and the average cost-effectiveness ratio, comparing the mAb and maternal strategies with no intervention.

2.2 Expected Value of Partially Perfect Information (EVPPI)

The EVPPI demonstrated how valuable obtaining perfect information about this input parameter would be. A selection of three countries are presented in the main text (Figure 4 and section EVPPI), whereas all 72 country specific EVPPI graphs are presented in S. Figure 7.

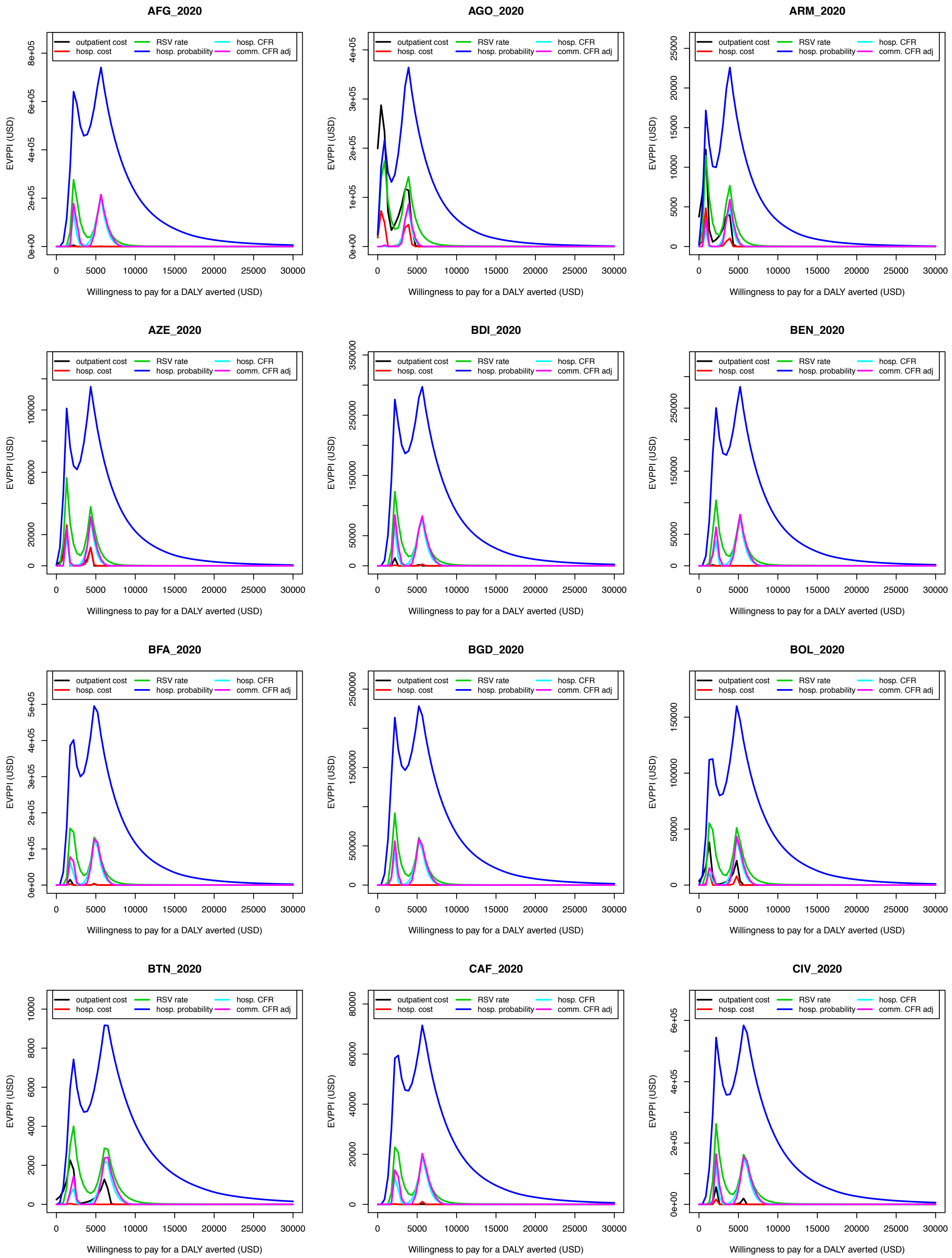
AFG_2020
AGO_2020
ARM_2020
AZE_2020
BDI_2020
BEN_2020
BFA_2020
BGD_2020
BOL_2020
BTN_2020
CAF_2020
CIV_2020
outpatient cost
hosp. cost
RSV rate
hosp. probability
hosp. CFR
comm. CFR adj
EVPPI (USD)
Willingness to pay for a DALY averted (USD)

125

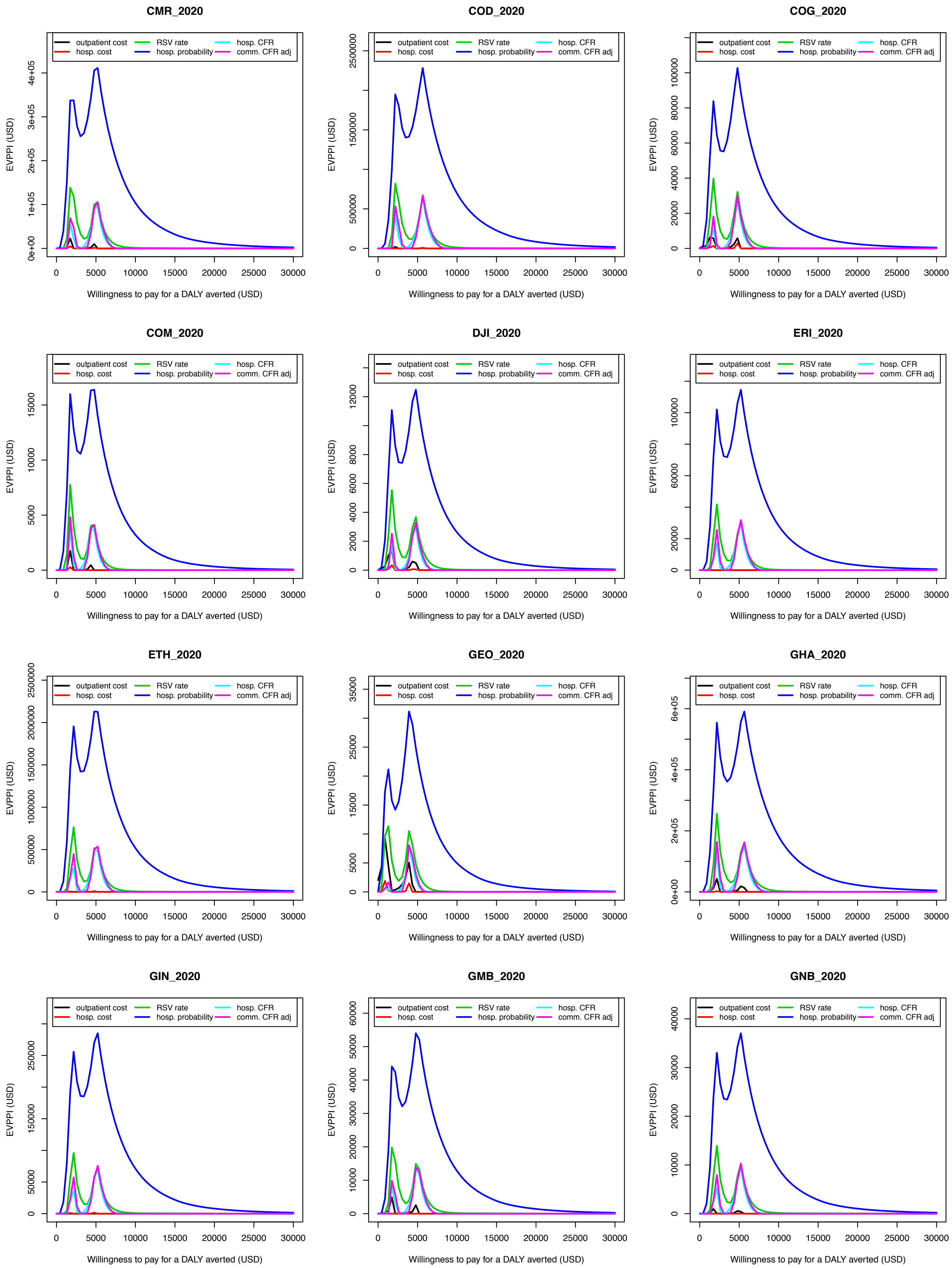

CMR_2020
COD_2020
COG_2020
COM_2020
DJI_2020
ERI_2020
ETH_2020
GEO_2020
GHA_2020
GIN_2020
GMB_2020
GNB_2020
outpatient cost
hosp. cost
RSV rate
hosp. probability
hosp. CFR
comm. CFR adj
EVPPI (USD)
Willingness to pay for a DALY averted (USD)

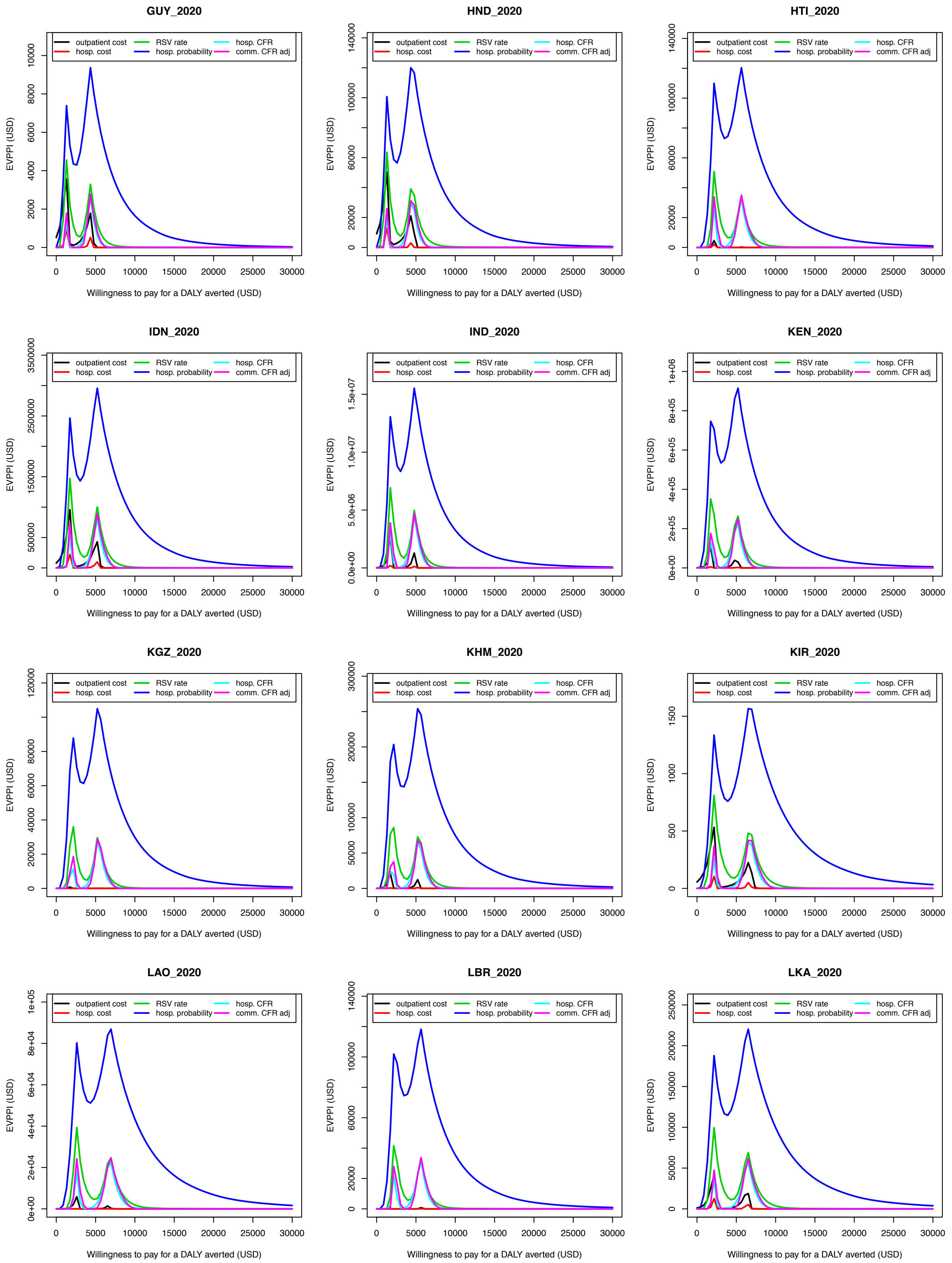
GUY_2020
HND_2020
HTI_2020
IDN_2020
IND_2020
KEN_2020
KGZ_2020
KHM_2020
KIR_2020
LAO_2020
LBR_2020
LKA_2020
outpatient cost
hosp. cost
RSV rate
hosp. probability
hosp. CFR
comm. CFR adj
EVPPI (USD)
Willingness to pay for a DALY averted (USD)
0
5000
10000
15000
20000
25000
30000

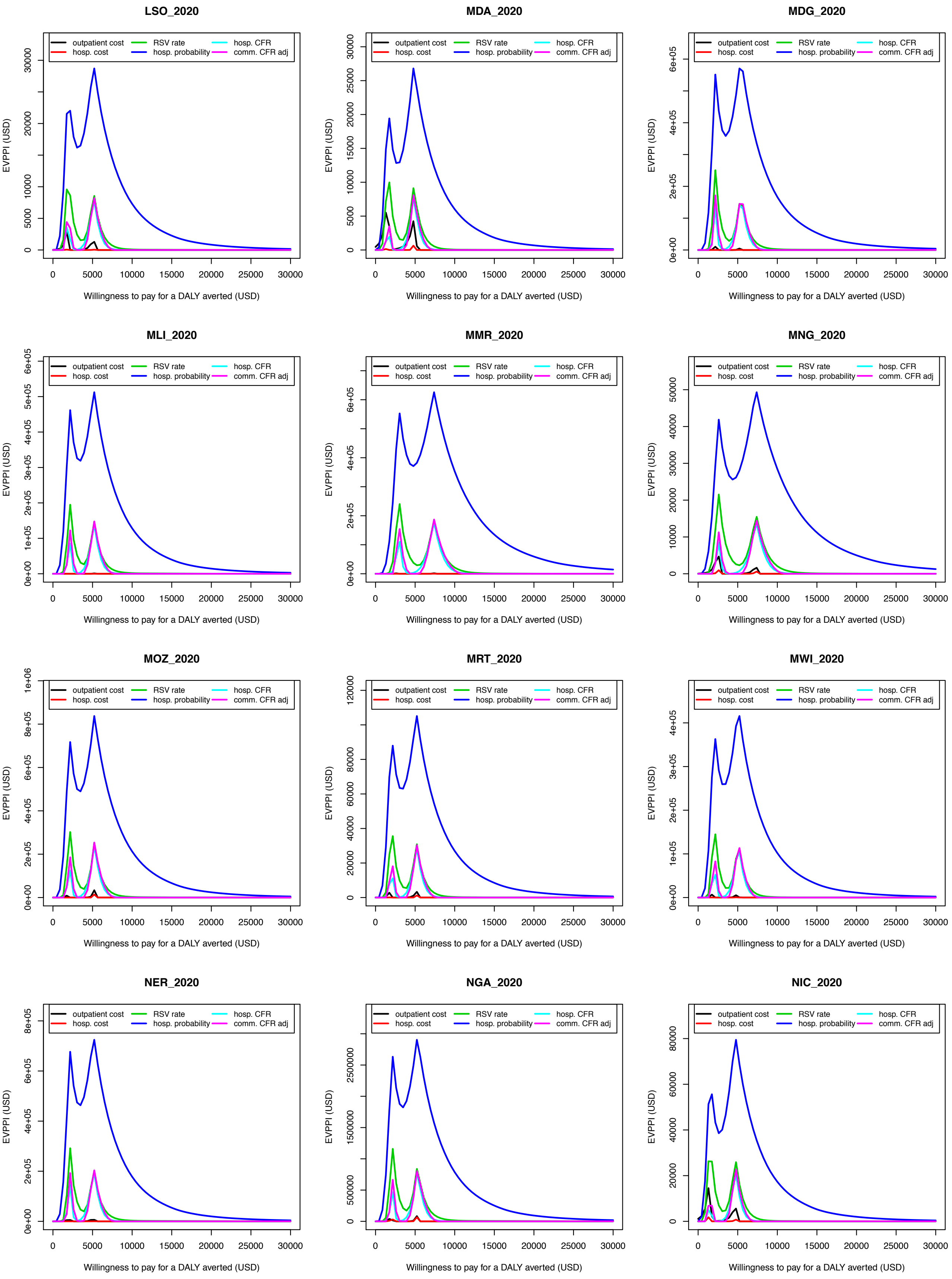
LSO_2020
MDA_2020
MDG_2020
MLI_2020
MMR_2020
MNG_2020
MOZ_2020
MRT_2020
MWI_2020
NER_2020
NGA_2020
NIC_2020
outpatient cost
hosp. cost
RSV rate
hosp. probability
hosp. CFR
comm. CFR adj
EVPPI (USD)
Willingness to pay for a DALY averted (USD)

127

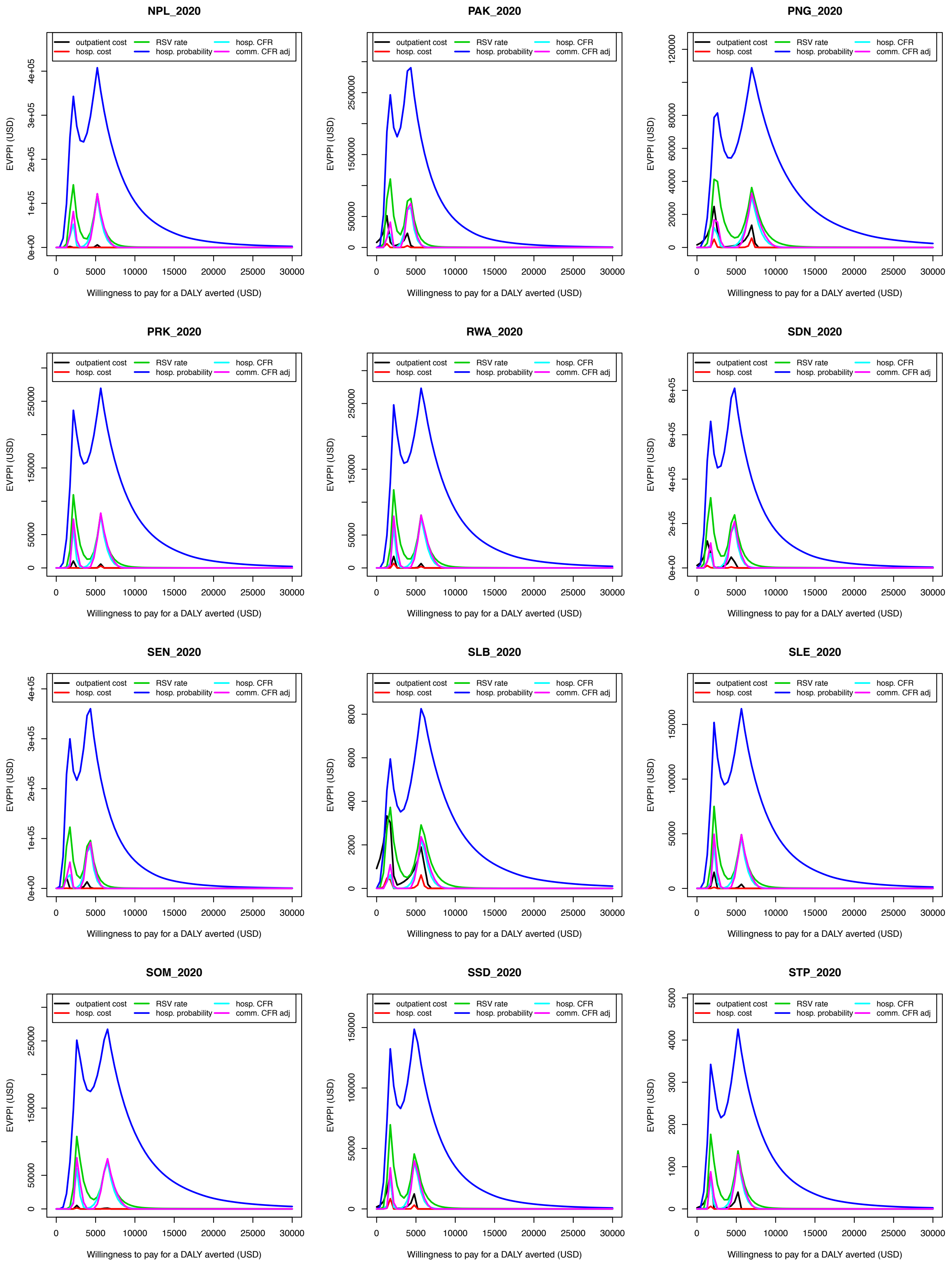
NPL_2020
PAK_2020
PNG_2020
PRK_2020
RWA_2020
SDN_2020
SEN_2020
SLB_2020
SLE_2020
SOM_2020
SSD_2020
STP_2020
outpatient cost
hosp. cost
RSV rate
hosp. probability
hosp. CFR
comm. CFR adj
EVPPI (USD)
Willingness to pay for a DALY averted (USD)

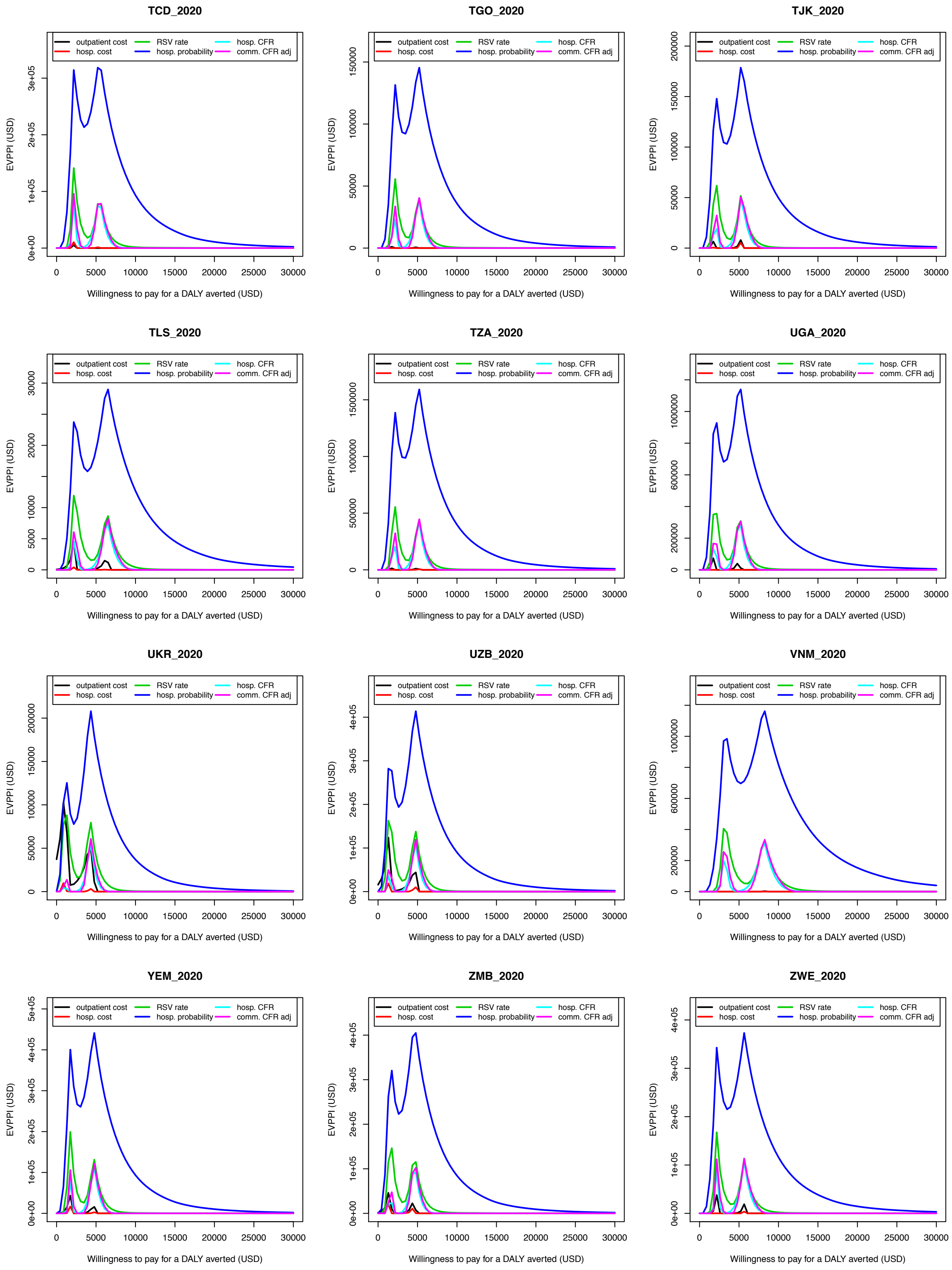

S. Figure 7: Expected Value of Partially Perfect Information (EVPPI) of all 72 countries

The Y-axis is adjusted per country

2.3 Scenario analysis: using pooled hospital probability

This scenario applied the pooled data (combining Nokes et al, 2008 and Homaira et al, 2012) on hospital probability to construct our hospital probability estimates instead of using only the hospital probability data from Nokes et al. S. Table 1 demonstrates that both maternal and the mAb strategies would prevent fewer RSV-associated hospital admissions and deaths than using the estimates of using only the data of Nokes et al, with increased uncertainty range. This is caused by the lower expected mean of the hospital probability and the wider uncertainty range when using the pooled analysis. Consequently, this analysis shows that the interventions would avert fewer discounted DALYs and hospital admission costs.

S. Table 1: Estimated mean costs and health outcomes averted by RSV interventions using pooled hospital probability data

RSV associated ('000)	No intervention		mAb (6m protection)	Maternal (5m protection)
	Under 1 year	1-4 years	Under 1 year	Under 1 year
RSV cases (Non-hospital +hospital cases)	7,649 [5,366 - 10,138]	13,192 [10,691 - 15,486]	5,928 [4,363 - 7,504]	6,464 [4,712 - 8,231]
Hospital admissions	280 [1 - 2,332]	478 [2 - 3,989]	216 [1 - 1,793]	236 [1 - 1,954]
Deaths	8 [0 - 60]	9 [0 - 72]	6 [0 - 45]	6 [0 - 49]
Discounted YLDs	14 [9 - 24]	22 [17 - 39]	11 [7 - 18]	12 [8 - 20]
Discounted YLLs	215 [1 - 1,713]	245 [1 - 1,957]	159 [1 - 1,272]	175 [1 - 1,402]
Discounted DALYs	228 [12 - 1,736]	268 [21 - 1,994]	170 [10 - 1,288]	186 [11 - 1,419]
Intervention costs (including delivery)	0	0 [0 - 0]	430,531 [430,531 - 430,531]	220,775 [220,775 - 220,775]
Discounted outpatient costs	165,759 [70,722 - 363,149]	285,859 [132,690 - 608,725]	128,944 [56,369 - 281,702]	140,402 [61,045 - 306,739]
Discounted hospital costs	31,408 [146 - 253,072]	50,765 [235 - 423,703]	24,571 [116 - 198,123]	26,691 [125 - 216,044]
Discounted total costs	197,166 [83,013 - 459,127]	321,091 [148,008 - 714,158]	584,046 [496,832 - 780,774]	387,868 [292,512 - 603,763]
Burden averted				
RSV cases averted	NA	NA	1,721 [979 - 2,660]	1,186 [640 - 1,924]
Hospital admission averted	NA	NA	63 [0 - 529]	44 [0 - 364]
Death averted	NA	NA	2 [0 - 16]	1 [0 - 11]
Discounted DALY averted	NA	NA	59 [3 - 450]	42 [2 - 325]
Net discounted costs	NA	NA	386,880 [324,186 - 414,244]	190,702 [145,263 - 210,059]

Estimated mean [95% credible interval] costs and health outcomes averted by RSV interventions summed over 72 Gavi countries in 2020 using pooled hospital probability data, and keeping all other parameters as in the base scenario in the main text using 70% efficacy for both strategies, 1 extra month of protection at 3USD more for mAb vs maternal. Costs are presented in USD2016. Outputs are discounted at a rate of 3% per year.

Compared with using only the Nokes et al hospital probability (base case), the maternal strategy has a markedly lower probability to become the optimal (most cost-effective) strategy, whereas the mAb strategy becomes optimal at a much higher WTP level. (S. Figure 8)

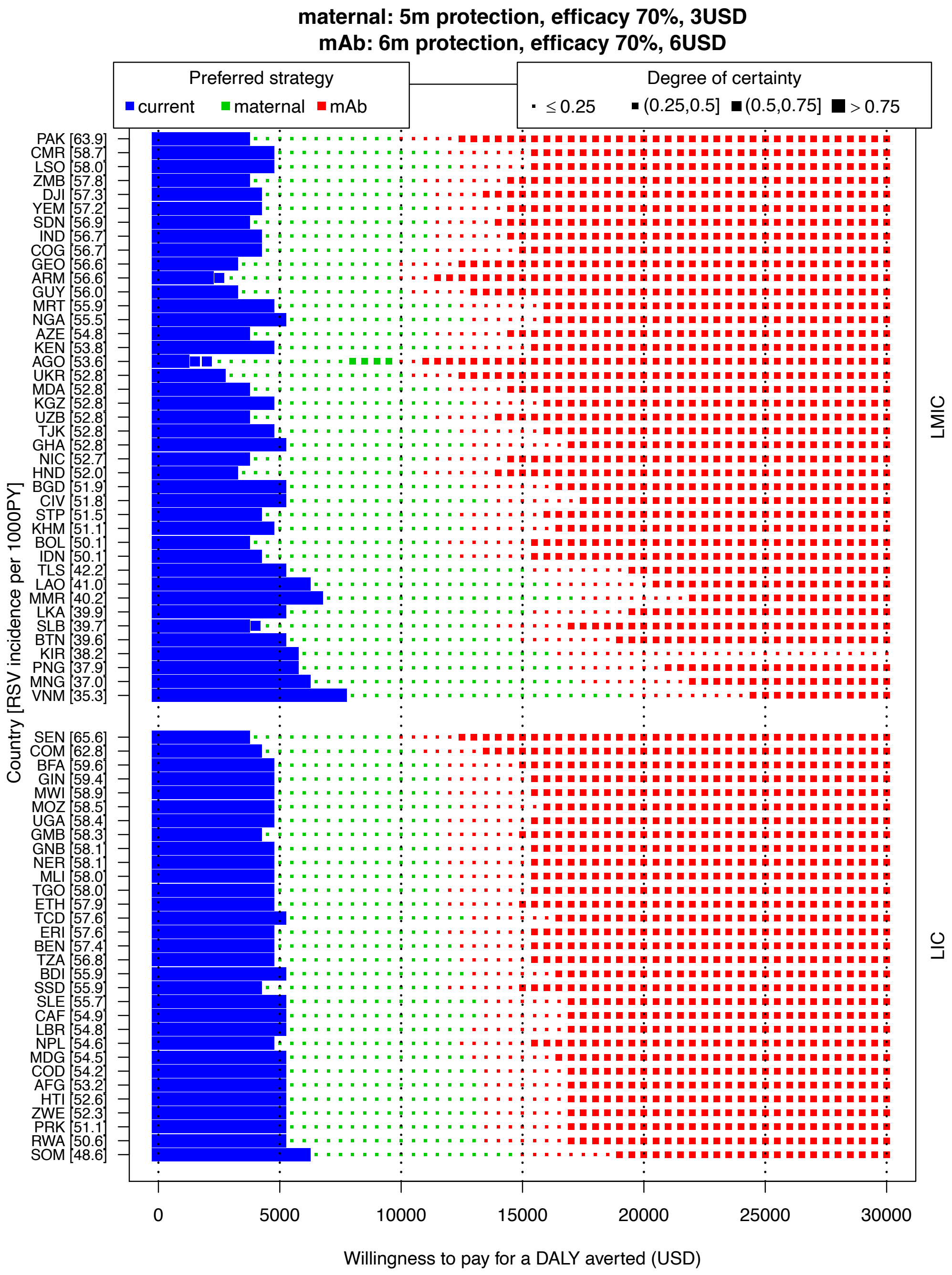

S. Figure 8: Cost-effectiveness analysis when using pooled hospital probability data

Cost-effectiveness analysis when using pooled hospital probability data to construct our estimates of hospital probability. The optimal strategy is shown by the colours (the current strategy is no intervention) and our certainty is indicated by the sizes of the markers. These results come from constructing a cost-effectiveness acceptability curve and a cost-effectiveness acceptability frontier for each country for a range of WTP values (0-30,000 USD per DALY averted). Countries are ranked by RSV incidence rate (high to low in left Y-axis [per 1000 person-year]) and stratified by income group (LIC or LMIC, on the right Y-axis).

2.4 Scenario analysis: without discounting

Costs and effects (DALYs) are discounted at 3% in our base case analysis. When they are not discounted, both the maternal and mAb strategies would avert more DALYs, however, they would not prevent more outpatient and hospital admission costs because these costs occur in the first year of life, and are not subject to discounting under our approach (S. Table 2). S. Figure 9 shows that the WTP values at which “switches” occur between no intervention and the maternal strategy, as well as from the maternal to the mAb strategy appear to be lower compared with the base case. The mAb strategy becomes the most cost-effective strategy at a WTP value of 4,000 USD. For the sake of space, we chose not to show scenarios using higher than 3% discount rates, although it should be clear that this would make the results less attractive for both the maternal and mAb strategies in comparison to the base scenario shown in the main text.

S. Table 2: Estimated mean costs and health outcomes averted by RSV interventions using 0% discounting rate

RSV associated (‘000)	No intervention		mAb (6m protection)	Maternal (5m protection)
	Under 1 year	1-4 years	Under 1 year	Under 1 year
RSV cases (Non-hospital +hospital cases)	7,649 [5,366 - 10,138]	13,192 [10,691 - 15,486]	5,928 [4,363 - 7,503]	6,464 [4,712 - 8,231]
Hospital admissions	668 [142 - 1,853]	1,150 [258 - 3,139]	517 [112 - 1,421]	564 [122 - 1,555]
Deaths	18 [3 - 55]	22 [4 - 68]	13 [2 - 40]	15 [3 - 44]
YLDs	16 [10 - 24]	27 [20 - 38]	12 [8 - 18]	13 [9 - 19]
YLLs	1,195 [206 - 3,611]	1,443 [250 - 4,454]	886 [157 - 2,634]	973 [172 - 2,906]
DALYs	1,210 [219 - 3,630]	1,470 [272 - 4,497]	898 [167 - 2,650]	986 [183 - 2,922]
Intervention costs (including delivery)	0	0 [0 - 0]	430,531 [430,531 - 430,531]	220,775 [220,775 - 220,775]
Outpatient costs	157,114 [69,436 - 341,707]	270,910 [128,069 - 577,098]	122,214 [55,209 - 264,714]	133,078 [59,878 - 288,301]
Hospital costs	75,254 [15,043 - 213,053]	129,391 [26,980 - 362,593]	58,975 [12,080 - 166,009]	64,033 [13,123 - 179,855]
Total costs	232,368 [112,538 - 443,860]	400,300 [205,404 - 736,753]	611,720 [521,121 - 770,475]	417,885 [319,308 - 592,027]
Burden averted				
RSV cases averted	NA	NA	1,721 [979 - 2,659]	1,186 [641 - 1,924]
Hospital admission averted	NA	NA	151 [29 - 443]	104 [19 - 309]
Death averted	NA	NA	5 [1 - 15]	3 [1 - 11]
DALY averted	NA	NA	312 [51 - 973]	224 [35 - 708]
Net costs	NA	NA	379,352 [322,875 - 408,824]	185,517 [144,452 - 206,446]

Estimated mean [95% credible interval] costs and health outcomes averted by RSV interventions summed over 72 Gavi countries using 0% discounting rate (in 2020). Constant parameters are: Nokes’ hospital probability data, efficacy (70% for both strategies) and duration of protection (maternal: 5 months vs. mAb: 6 months) and cost of interventions (maternal:3 USD vs. mAb: 6 USD per dose). Costs are presented in USD2016.

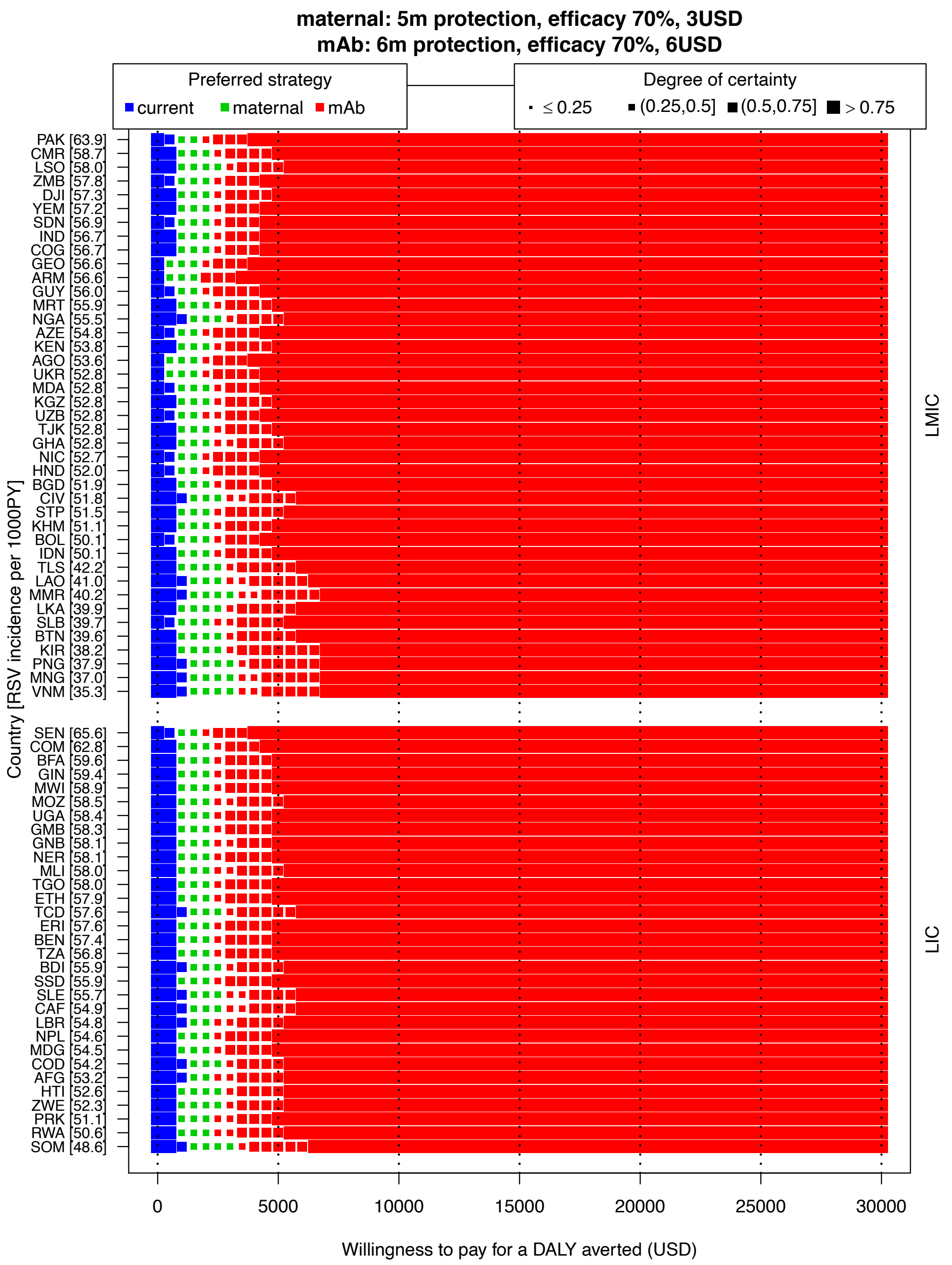

S. Figure 9: Cost-effectiveness analysis when assuming a 0% discount rate.

Cost-effectiveness analysis when assuming a 0% discount rate. The optimal strategy is shown by the colours (current strategy is no intervention) and our certainty is indicated by the sizes of the markers. These results come from constructing a cost-effectiveness acceptability curve and a cost-effectiveness acceptability frontier for each country for a range of WTP values (0-30,000 USD per DALY averted). Countries are ranked by RSV incidence rate (high to low in left Y-axis [per 1000 person-year]) and stratified by income group (LIC or LMIC, on the right Y-axis).

2.5 Scenario analysis: trial-based vaccine efficacy

Our analyses in the main text applied the same efficacy against symptomatic RSV-associated cases and hospitalisation. Recently, topline results of the first RSV maternal immunisation phase 3 trial (PrepareTM) have been made public; the trial vaccinated 4,636 pregnant women [9]. The efficacy of the maternal vaccine against primary and secondary endpoints are reported in S. Table 3.

S. Table 3:Reported vaccine efficacy in phase 3 PrepareTM trial

Endpoints	Per-protocol: RSV LRTI in infants through 90 days of life	Pre-specified exploratory analyses
Medically significant RSV LRTI (primary)	39% (97.5%CI, -1% to 64%)<br>Incidence in placebo arm: 35/1430<br>Incidence in product arm: 41/2765	41% (95%CI, 16% to 58%)<br>Incidence in placebo arm: 56/1430<br>Incidence in product arm: 64/2765
RSV LRTI hospitalisations (secondary)	44% (95%CI, 20% to 62%)<br>Incidence in placebo arm: 53/1430<br>Incidence in product arm: 57/2765	42% (95%CI, 17% to 59%)<br>Incidence in placebo arm: 55/1430<br>Incidence in product arm: 62/2765
RSV LRTI with severe hypoxemia (secondary)	48% (95%CI, -8% to 75%)<br>Incidence in placebo arm: 14/1430<br>Incidence in product arm: 14/2765	60% (95%CI, 32% to 76%)<br>Incidence in placebo arm: 32/1430<br>Incidence in product arm: 25/2765

We used vaccine efficacy estimates as a proxy for effectiveness. To account for the uncertainty around the average efficacy in our model, we used a lognormal distribution for relative risk, with its mean and standard error as expressed below:

$$mean\ [\ ln\ (Relative\ Risk)\ ] = ln(a) - ln(a + c) + ln(b + d) - ln(b)$$

$$standard\ error\ [\ ln(Relative\ Risk)\ ] = sqrt(a^{-1} - (a + c)^{-1} + b^{-1} + (b + d)^{-1}),$$

with ‘a’ and ‘a+c’ the number of cases and individuals in the placebo arm, respectively and ‘b’ and ‘b+d’ the number of cases and individuals in the product arm, respectively. Efficacy estimates were then obtained as one minus the relative risk. As such, we were able to capture the asymmetry of the reported confidence intervals. A very small proportion of samples resulted in negative efficacy estimates (about 0.2%), we replaced these by zero, which had no effect on the mean efficacy estimates and 95% confidence intervals up to two decimals. We sampled jointly from the uncertainty distributions of efficacy against the primary and secondary endpoint to account for their correlation (i.e. the efficacy estimates are based on the same dataset).

In absence of full analytical results of the trial, three scenarios were evaluated based on the reported maternal vaccine efficacy values, while maintaining the efficacy assumption of mAb at 70%. All details are provided in S. Table 4.

S. Table 4: Vaccine efficacy estimates for the trial-based scenarios

Scenario	Parameter	Vaccine efficacy against RSV-associated cases	Vaccine efficacy against RSV-associated hospitalisations and deaths
A. Overall Efficacy	Reported efficacy & source	41% (95%CI, 16% to 58%)<br>(Medically significant, exploratory analyses)	41% (95%CI, 16% to 58%)<br>(Medically significant, exploratory analyses)
	Model uncertainty distribution	lognormal<br>(mean=-0.5258, std.dev= 0.1839)	lognormal<br>(mean=-0.5258, std.dev= 0.1839)
	Model mean and 95% confidence interval	40% (95% CI 15% to 59%)	40% (95% CI 15% to 59%)
B. Most in favour of the maternal vaccine	Reported efficacy & source	41% (95%CI, 16% to 58%)<br>(Medically significant, exploratory analyses)	60% (95%CI, 32% to 76%)<br>(Severe hypoxemia, exploratory analysis)
	Model distribution	lognormal<br>(mean=-0.5258, std.dev= 0.1839)	lognormal<br>(mean=-0.9062, std.dev=0.2676)
	Model estimates	40% (95% CI 15% to 59%)	58% (95% CI, 31% to 76%)
C. Least in favour of the maternal vaccine	Reported efficacy & source	39% (97.5%CI, -1% to 64%)<br>(Medically significant, per-protocol)	42% (95%CI, 17% to 59%)<br>(Hospitalisation, exploratory analysis)
	Model distribution	lognormal<br>(mean=-0.5011, std.dev=0.2309)	lognormal<br>(mean=-0.5396, std.dev=0.1861)
	Model estimates	38% (95% CI 4% to 61%)<br>(97.5%CI 0% to 64%)	40% (95% CI 16% to 59%)

The results of the trial-based vaccine efficacy scenarios are summarized in S. Table 5. In general, the results of the 3 scenarios are quite similar. The maternal strategy averted more cases and hospitalisations when we used efficacy estimates most in favour of the vaccine.

Results per country are presented in S. Figure 10, in comparison with the base case (70% vaccine efficacy). Scenario A shows that when the maternal vaccine efficacy is at 40% (95%CI, 15% to 59%), the maternal strategy never becomes the most cost-effective strategy at any WTP level. In scenario B (most in favour of the vaccine), the maternal strategy becomes the most cost-effective strategy at WTP values of 1,800-3,800 USD per DALY averted, which is in line with the base case analysis (800-3,000 USD per DALY averted). For scenario C (least in favour of the vaccine), the marginal protection against RSV-associated hospitalisation (38% vs. 40%) is not enough to switch strategies.

In conclusion, when maternal vaccine offers sufficiently higher protection against hospitalisation (e.g. 58%), it would compensate its lower efficacy against the RSV-associated cases (e.g. 40%). However,

the mAb strategy becomes the optimal strategy at lower WTP values (2,000-6,000 vs. 4,000-8,000 USD per DALY averted) than in the base case.

S. Table 5: Estimated mean costs and health outcomes (in thousand) in children under 1 using trial-based-efficacy values

RSV associated (‘000)	No intervention	Senario A<br>Maternal (5m protection)<br>overall efficacy at 40%	Senario B<br>Maternal (5m protection)<br>Most in favour of the vaccine	Senario C<br>Maternal (5m protection)<br>Least in favour of the vaccine
	Under 1 year	Under 1 year	Under 1 year	Under 1 year
RSV cases (Non-hospital +hospital cases)	7,649 [5,366 - 10,138]	6,979 [4,998 - 9,114]	6,977 [4,994 - 9,114]	7,012 [5,018 - 9,220]
Hospital admissions	668 [142 - 1,853]	609 [130 - 1,685]	582 [125 - 1,612]	608 [130 - 1,682]
Deaths	18 [3 - 55]	16 [3 - 49]	15 [3 - 46]	16 [3 - 48]
Discounted YLDs	16 [10 - 24]	14 [9 - 21]	14 [9 - 21]	14 [9 - 21]
Discounted YLLs	517 [89 - 1,561]	462 [80 - 1,386]	437 [76 - 1,312]	461 [79 - 1,383]
Discounted DALYs	532 [102 - 1,581]	477 [91 - 1,404]	451 [87 - 1,330]	476 [91 - 1,400]
Intervention costs (including delivery)	NA	220,775 [220,775 - 220,775]	220,775 [220,775 - 220,775]	220,775 [220,775 - 220,775]
Discounted outpatient costs	157,114<br>[69,436 - 341,707]	143,509 [63,740 - 312,907]	144,073 [64,161 - 314,132]	144,285 [63,872 - 312,614]
Discounted hospital costs	75,254<br>[15,043 - 213,053]	68,905 [13,919 - 196,544]	65,972 [13,375 - 188,187]	68,766 [13,879 - 196,195]
Discounted total costs	232,368<br>[112,538 - 443,860]	433,189<br>[324,845 - 623,143]	430,776<br>[323,610 - 617,882]	433,781<br>[324,875 - 624,539]
Burden averted				
RSV cases averted	NA	671 [216 - 1,267]	671 [215 - 1,267]	635 [64 - 1,293]
Hospital admissions averted	NA	59 [8 - 180]	86 [14 - 257]	60 [9 - 183]
Deaths averted	NA	2 [0 - 7]	3 [0 - 9]	2 [0 - 7]
Discounted DALYs averted	NA	56 [7 - 191]	81 [12 - 269]	57 [7 - 194]
Net discounted costs	NA	200,821<br>[172,847 - 215,562]	198,459<br>[167,588 - 214,585]	201,464<br>[172,132 - 218,016]

Estimated mean [95% credible interval] costs and health outcomes (in thousand) pre- and post-RSV interventions in children under 1 year, summed over 72 Gavi countries (in 2020) using trial-based efficacy values. Constant parameters are: Nokes’ hospital probability data, duration of protection (maternal: 5 months and mAb: 6 months) and cost of interventions (maternal:3 USD and mAb. 6 USD per dose). Costs are presented in USD2016. Outputs are discounted at a rate of 3% per year

Maternal vaccine efficacy 70% (base case in the main text)

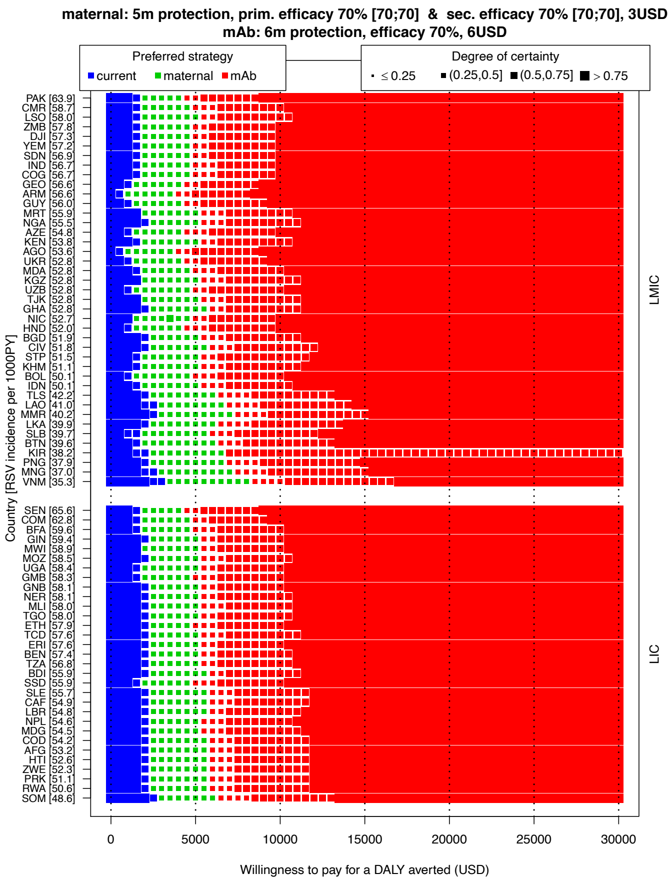

Senario A (maternal vaccine efficacy 40%, mAb efficacy 70%)

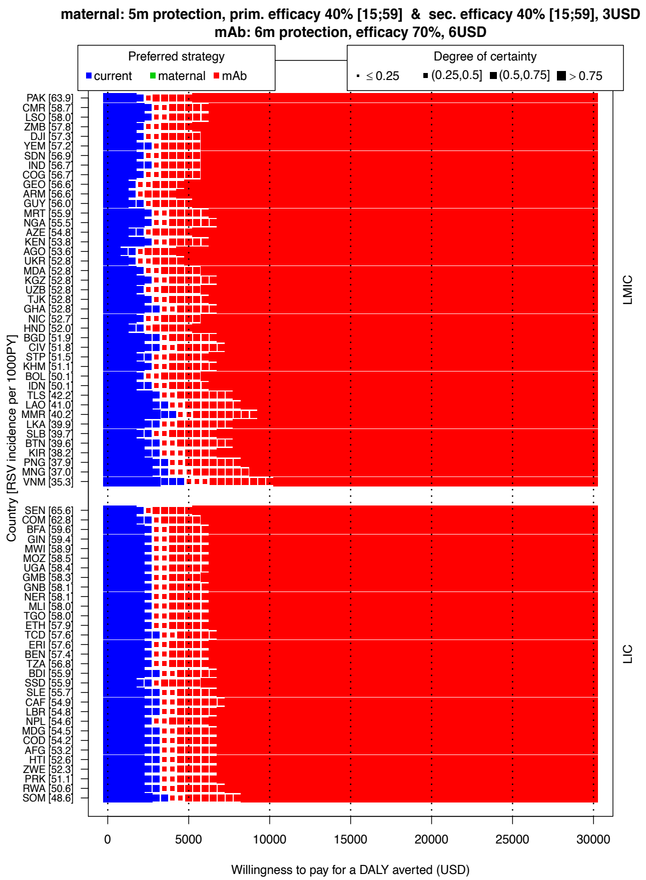

Scenario B: most in favour of the maternal vaccine: 40% and 58% efficacy (fixed 70% efficacy for mAb)

Scenario C: least in favour of the maternal vaccine 38% and 40% efficacy (fixed 70% efficacy for mAb)

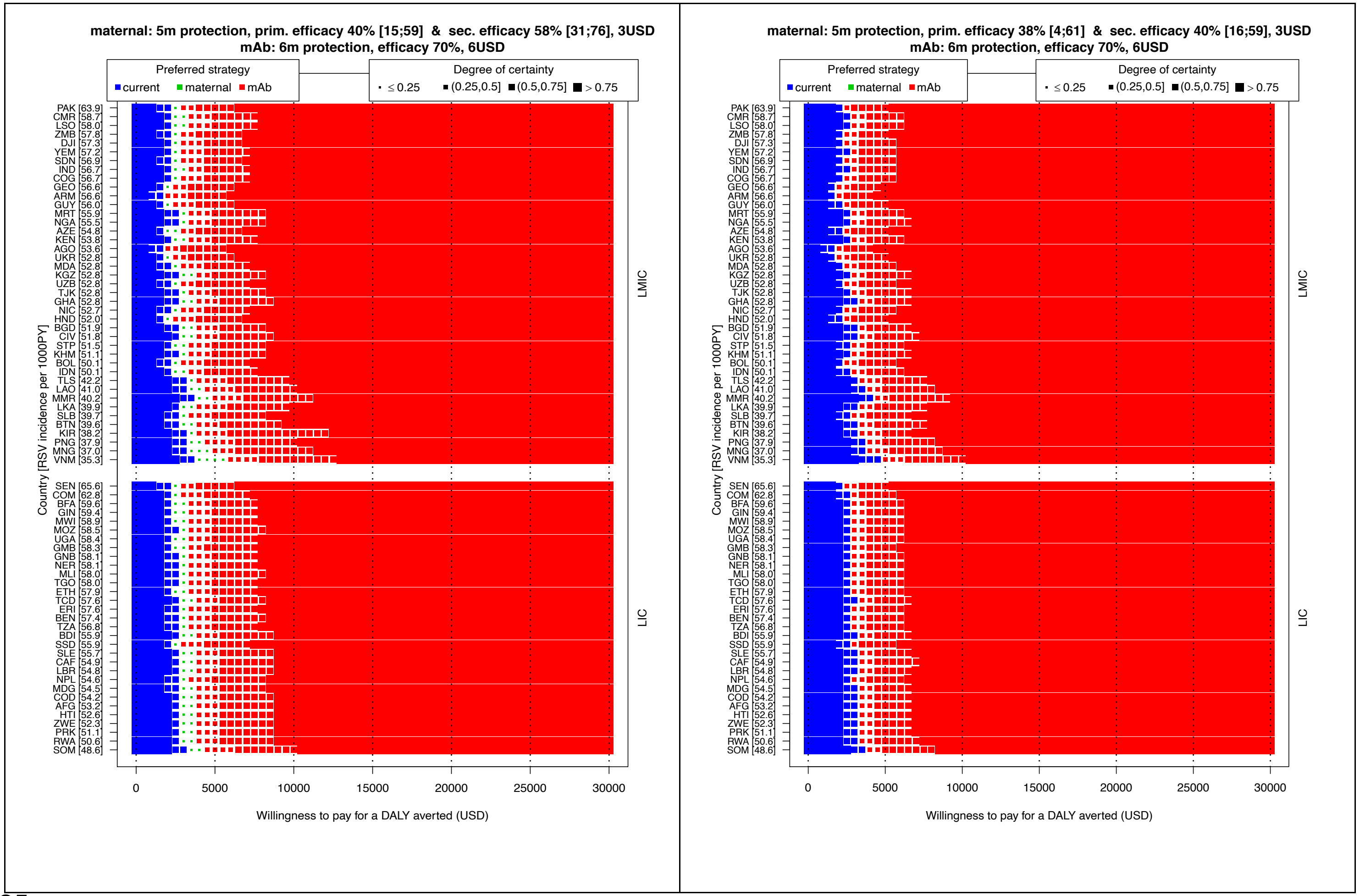

S. Figure 10: Cost-effectiveness analysis for different scenarios related to the trial-based maternal vaccine efficacy

Cost-effectiveness analysis for different scenarios related to the trial-based maternal vaccine efficacy. The optimal strategy for a WTP value is shown by the colours (the current strategy is no intervention) and our certainty is indicated by the sizes of the markers. These results come from constructing a cost-effectiveness acceptability curve and a cost-effectiveness acceptability frontier for each country for a range of WTP values (0-30,000 USD per DALY averted). Countries are ranked by RSV incidence rate (high to low in left Y-axis [per 1000 person-year]) and stratified by income group (LIC or LMIC, on the right Y-axis).

3 References

1. Trevor HT, R; Friedman, J: **The Elements of Statistical Learning:**: Springer; 2009.
2. Nair H, Nokes DJ, Gessner BD, Dherani M, Madhi SA, Singleton RJ, O'Brien KL, Roca A, Wright PF, Bruce N *et al*: **Global burden of acute lower respiratory infections due to respiratory syncytial virus in young children: a systematic review and meta-analysis**. *Lancet* 2010, **375**(9725):1545-1555.
3. Shi T, McAllister DA, O'Brien KL, Simoes EAF, Madhi SA, Gessner BD, Polack FP, Balsells E, Acacio S, Aguayo C *et al*: **Global, regional, and national disease burden estimates of acute lower respiratory infections due to respiratory syncytial virus in young children in 2015: a systematic review and modelling study**. *Lancet* 2017, **390**(10098):946-958.
4. Nokes DJ, Okiro EA, Ngama M, Ochola R, White LJ, Scott PD, English M, Cane PA, Medley GF: **Respiratory syncytial virus infection and disease in infants and young children observed from birth in Kilifi District, Kenya**. *Clin Infect Dis* 2008, **46**(1):50-57.
5. Homaira N, Luby SP, Petri WA, Vainionpaa R, Rahman M, Hossain K, Snider CB, Rahman M, Alamgir AS, Zesmin F *et al*: **Incidence of respiratory virus-associated pneumonia in urban poor young children of Dhaka, Bangladesh, 2009-2011**. *PLoS One* 2012, **7**(2):e32056.
6. Briggs AC, K. Sculpher, M: **Decision Modelling for Health Economic Evaluation**. Oxford: Oxford University Press; 2006.
7. Stenberg K, Lauer JA, Gkountouras G, Fitzpatrick C, Stanciole A: **Econometric estimation of WHO-CHOICE country-specific costs for inpatient and outpatient health service delivery**. *Cost Eff Resour Alloc* 2018, **16**:11.
8. Zhang S, Sammon PM, King I, Andrade AL, Toscano CM, Araujo SN, Sinha A, Madhi SA, Khandaker G, Yin JK *et al*: **Cost of management of severe pneumonia in young children: systematic analysis**. *J Glob Health* 2016, **6**(1):010408.
9. Novavax: **Novavax Announces Topline Results from Phase 3 PrepareTM Trial of ResVax™ for Prevention of RSV Disease in Infants via Maternal Immunization**. In. Novavax website; 2019.

4 Long tables

S. Table 6: Estimated age-specific RSV cases in each country

Country	ISO3	Under 3months			3-5 months			6to11 months			1to5 years			Population under 5
		RSV cases	Hospital cases	Deaths	RSV cases	Hospital cases	Deaths	RSV cases	Hospital cases	Deaths	RSV cases	Hospital cases	Deaths	
Afghanistan	AFG	6,844	60	23	29,821	2,607	77	62,526	5,454	135	170,629	14,872	283	5,067,270
Angola	AGO	7,294	640	24	31,725	2,773	82	66,301	5,784	143	179,602	15,654	298	5,311,239
Armenia	ARM	253	22	1	1,109	97	3	2,356	206	5	6,521	568	11	180,926
Azerbaijan	AZE	1,050	92	3	4,597	402	12	9,709	847	21	26,739	2,331	44	768,347
Burundi	BDI	2,750	241	9	11,943	1,044	31	24,895	2,172	54	67,090	5,848	111	1,907,468
Benin	BEN	2,557	224	8	11,115	972	29	23,211	2,025	50	62,739	5,468	104	1,737,026
BurkinaFaso	BFA	4,899	430	16	21,344	1,866	55	44,745	3,903	97	121,617	10,600	202	3,230,312
Bangladesh	BGD	18,290	1,605	61	80,094	7,001	206	169,398	14,777	366	466,760	40,684	775	14,159,441
Bolivia	BOL	1,459	128	5	6,379	558	16	13,456	1,174	29	36,844	3,211	61	1,159,411
Bhutan	BTN	67	6	0	293	26	1	619	54	1	1,708	149	3	67,874
Central African Republic	CAF	942	83	3	4,079	357	11	8,463	738	18	22,666	1,976	38	658,371
Côte d'Ivoire	CIV	5,102	448	17	22,211	1,942	57	46,502	4,057	101	126,304	11,009	210	3,860,241
Cameroon	CMR	5,586	490	19	24,306	2,125	63	50,839	4,435	110	137,878	12,018	229	3,722,449
Democratic Republicoft he Congo	COD	20,098	1,763	67	87,350	7,636	225	182,309	15,903	394	492,805	42,953	819	14,432,777
Congo	COG	1,164	102	4	5,081	444	13	10,695	933	23	29,280	2,552	49	815,693
Comoros	COM	182	16	1	792	69	2	1,658	145	4	4,517	394	7	113,907
Djibouti	DJI	136	12	0	594	52	2	1,244	108	3	3,380	295	6	93,485
Eritrea	ERI	1,054	93	3	4,613	403	12	9,742	850	21	26,793	2,335	44	733,293
Ethiopia	ETH	21,497	1,886	71	93,997	8,217	242	198,278	17,297	429	543,673	47,388	903	14,799,645
Georgia	GEO	342	30	1	1,502	131	4	3,192	278	7	8,842	771	15	245,232
Ghana	GHA	5,238	460	17	22,871	1,999	59	48,126	4,198	104	131,567	11,468	218	3,938,308
Guinea	GIN	2,993	263	10	13,055	1,141	34	27,423	2,392	59	74,714	6,512	124	1,989,709
Gambia	GMB	534	47	2	2,327	203	6	4,887	426	11	13,315	1,161	22	361,473
Guinea-Bissau	GNB	410	36	1	1,781	156	5	3,712	324	8	9,988	871	17	273,468

Guyana	GUY	100	9	0	439	38	1	926	81	2	2,544	222	4	71,595
Honduras	HND	1,210	106	4	5,300	463	14	11,211	978	24	30,859	2,690	51	934,603
Haiti	HTI	1,530	134	5	6,677	584	17	14,038	1,225	30	38,262	3,335	64	1,151,138
Indonesia	IDN	28,695	2,518	95	125,732	10,991	324	266,201	23,222	576	734,454	64,017	1,219	23,060,901
India	IND	163,157	14,315	540	713,225	62,347	1,837	1,503,865	131,189	3,252	4,129,534	359,939	6,855	114,873,924
Kenya	KEN	9,486	832	31	41,456	3,624	107	87,368	7,621	189	239,613	20,885	398	7,028,272
Kyrgyzstan	KGZ	905	79	3	3,968	347	10	8,414	734	18	23,255	2,027	39	692,207
Cambodia	KHM	2,194	193	7	9,620	841	25	20,383	1,778	44	56,284	4,906	93	1,732,417
Kiribati	KIR	14	1	-	61	5	0	128	11	0	351	31	1	14,506
LaoPeople's Democratic Republic	LAO	742	65	2	3,241	283	8	6,825	595	15	18,722	1,632	31	720,784
Liberia	LBR	997	87	3	4,351	380	11	9,151	798	20	25,034	2,182	42	720,928
SriLanka	LKA	1,482	130	5	6,510	569	17	13,846	1,208	30	38,384	3,346	64	1,509,446
Lesotho	LSO	400	35	1	1,743	152	4	3,661	319	8	10,008	872	17	272,445
Republicof Moldova	MDA	253	22	1	1,112	97	3	2,360	206	5	6,531	569	11	194,256
Madagascar	MDG	5,344	469	18	23,394	2,045	60	49,445	4,313	107	135,930	11,848	226	3,932,142
Mali	MLI	4,881	428	16	21,224	1,855	55	44,332	3,867	96	119,781	10,440	199	3,279,424
Myanmar	MMR	4,293	377	14	18,730	1,637	48	39,360	3,434	85	107,723	9,389	179	4,233,099
Mongolia	MNG	303	27	1	1,328	116	3	2,815	246	6	7,774	678	13	330,358
Mozambique	MOZ	7,389	648	24	32,117	2,807	83	67,046	5,849	145	181,427	15,813	301	4,919,848
Mauritania	MRT	914	80	3	3,971	347	10	8,282	723	18	22,437	1,956	37	637,248
Malawi	MWI	4,479	393	15	19,479	1,703	50	40,701	3,550	88	110,742	9,652	184	2,979,871
Niger	NER	6,518	572	22	28,380	2,481	73	59,423	5,184	129	160,639	14,002	267	4,387,626
Nigeria	NGA	43,388	3,807	144	188,680	16,493	486	394,194	34,387	853	1,064,501	92,783	1,768	30,454,506
Nicaragua	NIC	741	65	2	3,248	284	8	6,890	601	15	19,048	1,660	32	567,996
Nepal	NPL	3,641	319	12	15,944	1,394	41	33,717	2,941	73	92,940	8,101	154	2,679,582
Pakistan	PAK	37,366	3,278	124	162,381	14,194	418	338,872	29,561	733	920,510	80,233	1,528	22,848,453
PapuaNewGuinea	PNG	973	85	3	4,243	371	11	8,911	777	19	24,357	2,123	40	1,015,796
Democratic People'sRepublicofKorea	PRK	2,133	187	7	9,363	818	24	19,875	1,734	43	54,963	4,791	91	1,690,022

Rwanda	RWA	2,148	188	7	9,384	820	24	19,760	1,724	43	54,133	4,718	90	1,689,224
Sudan	SDN	8,446	741	28	36,845	3,221	95	77,400	6,752	167	211,083	18,398	351	5,870,091
Senegal	SEN	4,165	365	14	18,218	1,592	47	38,455	3,355	83	105,722	9,215	176	2,540,727
SolomonIslands	SLB	79	7	0	348	30	1	736	64	2	2,030	177	3	80,476
SierraLeone	SLE	1,546	136	5	6,704	586	17	13,935	1,216	30	37,505	3,269	62	1,071,324
Somalia	SOM	3,284	288	11	14,258	1,246	37	29,701	2,591	64	79,911	6,965	133	2,615,103
SouthSudan	SSD	2,717	238	9	11,810	1,032	30	24,652	2,150	53	66,587	5,804	111	1,891,616
SaoTomeandPrincipe	STP	40	3	0	173	15	0	363	32	1	992	86	2	30,442
Chad	TCD	3,832	336	13	16,603	1,451	43	34,464	3,006	75	92,197	8,036	153	2,552,992
Togo	TGO	1,672	147	6	7,286	637	19	15,276	1,333	33	41,541	3,621	69	1,134,834
Tajikistan	TJK	1,526	134	5	6,672	583	17	14,070	1,227	30	38,650	3,369	64	1,154,235
Timor-Leste	TLS	217	19	1	950	83	2	2,006	175	4	5,514	481	9	206,013
UnitedRepublicofTanzania	TZA	14,062	1,234	47	61,402	5,367	158	129,209	11,271	279	353,687	30,828	587	9,836,599
Uganda	UGA	11,657	1,023	39	50,755	4,437	131	106,268	9,270	230	288,681	25,162	479	7,826,974
Ukraine	UKR	2,926	257	10	12,853	1,124	33	27,332	2,384	59	75,759	6,603	126	2,251,488
Uzbekistan	UZB	4,005	351	13	17,528	1,532	45	37,029	3,230	80	101,982	8,889	169	3,041,357
VietNam	VNM	6,522	572	22	28,602	2,500	74	60,641	5,290	131	167,533	14,603	278	7,460,242
Yemen	YEM	5,626	494	19	24,550	2,146	63	51,610	4,502	112	141,186	12,306	234	3,899,965
Zambia	ZMB	4,251	373	14	18,542	1,621	48	38,947	3,398	84	106,320	9,267	177	2,905,622
Zimbabwe	ZWE	3,135	275	10	13,678	1,196	35	28,752	2,508	62	78,592	6,850	130	2,375,407

S. Table 7: Average outpatient and inpatient costs of the 72 Gavi countries in USD at 2016 value

Country	ISO3	Income Region	Outpatient mean [95% CI#]	Inpatient mean [95% CI#]
Afghanistan	AFG	LIC	10.46 [1.01 - 37.71]	24.25 [7.30 - 54.47]
Angola	AGO	LMIC	134.96 [9.35 - 491.89]	640.91 [189.90 - 1442.09]
Armenia	ARM	LMIC	107.91 [6.17 - 404.85]	511.37 [146.30 - 1142.78]
Azerbaijan	AZE	UMIC	92.73 [7.90 - 331.96]	540.45 [159.88 - 1218.00]
Bangladesh*	BGD	LMIC	10.93 [3.58 - 26.17]	174.60 [167.86 - 181.45]
Benin	BEN	LIC	29.48 [1.98 - 111.16]	92.28 [30.08 - 203.06]

Bhutan	BTN	LMIC	79.28 [5.68 - 287.22]	386.51 [107.01 - 884.37]
Bolivia	BOL	LMIC	94.02 [8.61 - 332.93]	424.18 [120.09 - 985.44]
Burkina Faso	BFA	LIC	24.69 [1.71 - 92.28]	72.99 [21.13 - 160.88]
Burundi	BDI	LIC	12.51 [0.96 - 44.78]	27.79 [7.92 - 64.23]
Côte d'Ivoire	CIV	LMIC	38.40 [3.28 - 140.51]	136.98 [37.17 - 308.85]
Cambodia	KHM	LMIC	41.20 [3.53 - 150.57]	152.53 [42.78 - 336.04]
Cameroon	CMR	LMIC	25.75 [2.18 - 90.54]	67.87 [19.71 - 151.32]
Central African Republic	CAF	LIC	26.06 [1.89 - 94.42]	78.58 [22.10 - 182.50]
Chad	TCD	LIC	34.28 [3.50 - 116.07]	95.47 [26.97 - 219.45]
Comoros	COM	LIC	52.60 [4.17 - 194.75]	233.90 [73.80 - 514.33]
Congo	COG	LMIC	42.90 [3.95 - 160.76]	143.85 [40.55 - 331.64]
Democratic People's Republic of Korea	PRK	LIC	353.45 [26.91 - 1303.18]	2043.01 [549.38 - 4602.86]
Democratic Republic of the Congo	COD	LIC	10.44 [1.04 - 36.23]	24.30 [7.23 - 52.11]
Djibouti	DJI	LMIC	12.73 [1.19 - 45.44]	25.70 [6.79 - 58.16]
Eritrea	ERI	LIC	66.64 [6.76 - 225.98]	240.40 [68.18 - 534.79]
Ethiopia	ETH	LIC	14.34 [4.19 - 36.84]	31.00 [9.09 - 70.78]
Gambia	GMB	LIC	23.10 [1.87 - 83.92]	62.55 [17.94 - 138.24]
Georgia	GEO	LMIC	28.73 [2.54 - 98.84]	101.28 [28.14 - 235.47]
Ghana	GHA	LMIC	94.08 [8.59 - 325.28]	428.56 [123.13 - 944.32]
Guinea*	GIN	LIC	30.92 [2.20 - 114.36]	103.74 [26.74 - 242.74]
Guinea-Bissau	GNB	LIC	2.72 [0.60 - 7.88]	95.77 [19.80 - 290.47]
Guyana	GUY	UMIC	25.99 [1.91 - 98.39]	76.17 [24.57 - 168.22]
Haiti	HTI	LIC	82.28 [5.44 - 288.37]	330.88 [97.32 - 741.20]
Honduras	HND	LMIC	24.72 [1.68 - 88.91]	70.48 [21.32 - 154.10]
India*	IND	LMIC	89.91 [7.31 - 329.05]	381.34 [109.02 - 870.73]
Indonesia*	IDN	LMIC	41.83 [3.50 - 148.69]	58.20 [21.76 - 123.97]
Kenya*	KEN	LMIC	62.42 [4.66 - 224.05]	176.77 [39.14 - 521.02]
Kiribati	KIR	LMIC	41.26 [3.36 - 143.99]	176.21 [117.77 - 251.27]
Kyrgyzstan	KGZ	LMIC	90.62 [7.48 - 330.93]	321.58 [97.87 - 735.09]
Lao PDR	LAO	LMIC	38.20 [2.51 - 147.94]	129.64 [35.10 - 296.29]
Lesotho	LSO	LMIC	44.94 [4.37 - 150.20]	169.49 [51.83 - 374.98]
Liberia	LBR	LIC	33.95 [2.97 - 122.27]	110.42 [33.29 - 243.20]
Madagascar	MDG	LIC	0.24 [0.01 - 1.04]	0.37 [0.10 - 0.88]
Malawi	MWI	LIC	14.64 [1.22 - 52.11]	38.02 [11.11 - 86.21]

Mali	MLI	LIC	12.66 [0.99 - 46.95]	32.98 [9.40 - 73.50]
Mauritania	MRT	LMIC	25.12 [2.09 - 90.71]	73.55 [21.68 - 162.18]
Mongolia	MNG	LMIC	33.69 [2.88 - 123.15]	118.92 [35.57 - 258.09]
Mozambique	MOZ	LIC	56.71 [5.10 - 200.37]	258.94 [76.94 - 576.35]
Myanmar	MMR	LMIC	16.27 [1.42 - 57.76]	42.83 [13.13 - 93.44]
Nepal	NPL	LIC	14.67 [1.20 - 55.47]	41.59 [11.36 - 92.26]
Nicaragua	NIC	LMIC	21.26 [1.93 - 77.57]	65.88 [16.38 - 155.18]
Niger	NER	LIC	65.24 [6.45 - 223.20]	288.48 [82.32 - 646.33]
Nigeria	NGA	LMIC	15.06 [1.03 - 55.68]	32.54 [9.50 - 73.75]
Pakistan*	PAK	LMIC	45.23 [3.39 - 167.33]	167.53 [50.32 - 376.52]
Papua New Guinea	PNG	LMIC	42.94 [3.75 - 196.12]	138.20 [24.26 - 455.46]
Republic of Moldova	MDA	LMIC	75.90 [5.62 - 285.71]	272.80 [71.71 - 630.77]
Rwanda	RWA	LIC	54.79 [4.17 - 190.71]	223.97 [69.10 - 490.82]
Sao Tome and Principe	STP	LMIC	26.11 [2.22 - 96.64]	70.86 [18.69 - 166.28]
Senegal	SEN	LIC	50.51 [4.23 - 189.49]	175.04 [51.12 - 387.79]
Sierra Leone	SLE	LIC	36.36 [3.38 - 133.30]	120.82 [37.21 - 267.81]
Solomon Islands	SLB	LMIC	22.27 [1.65 - 80.45]	61.53 [19.38 - 136.65]
Somalia	SOM	LIC	125.59 [11.12 - 453.43]	478.38 [135.09 - 1079.00]
South Sudan	SSD	LIC	14.28 [4.29 - 36.36]	30.89 [8.81 - 68.57]
Sri Lanka	LKA	LMIC	67.96 [3.76 - 254.66]	252.61 [73.33 - 566.73]
Sudan	SDN	LMIC	72.88 [5.57 - 258.76]	341.13 [102.88 - 746.80]
Tajikistan	TJK	LMIC	69.78 [3.68 - 265.64]	252.18 [68.48 - 579.54]
Timor-Leste	TLS	LMIC	27.40 [2.12 - 98.05]	94.80 [27.64 - 211.70]
Togo	TGO	LIC	54.87 [3.92 - 196.47]	167.47 [45.93 - 384.45]
Uganda	UGA	LIC	21.40 [1.88 - 79.03]	58.81 [19.05 - 125.51]
Ukraine	UKR	LMIC	22.55 [1.87 - 81.41]	68.14 [18.00 - 151.01]
Tanzania	TZA	LIC	76.74 [6.09 - 284.31]	387.79 [112.96 - 858.65]
Uzbekistan	UZB	LMIC	24.44 [1.98 - 88.68]	72.99 [19.62 - 166.73]
Viet Nam*	VNM	LMIC	73.12 [6.32 - 268.97]	282.71 [85.92 - 626.02]
Yemen	YEM	LMIC	5.92 [1.33 - 16.84]	58.82 [48.52 - 70.40]
Zambia*	ZMB	LMIC	64.19 [6.24 - 225.95]	244.25 [72.41 - 546.89]
Zimbabwe	ZWE	LIC	52.07 [12.06 - 149.68]	240.22 [53.62 - 718.79]

*# CI: credible interval; * indicates the country specific treatment costs estimated by meta-analysis of multiple costs data*

S. Table 8: Country-specific estimated mean of RSV-associated disease burden and the impact of interventions

Country	ISO3	Cases averted	Hospital admission averted	Deaths Averted	DALYs Averted	Averted Treatment Costs ('000)	Cost of intervention (inc delivery costs) ('000)	Incremental costs ('000)	Average cost-effectiveness ratio
mAb vs. no intervention									
Afghanistan	AFG	22328 [12706 - 34470]	1953 [378 - 5743]	61 [10 - 190]	1767 [301 - 5464]	176 [32 - 541]	5815 [5815 - 5815]	5640 [5274 - 5783]	5507 [1018 - 18979]
Angola	AGO	15842 [9028 - 24422]	1386 [269 - 4071]	43 [7 - 135]	1240 [212 - 3838]	2226 [419 - 6951]	4119 [4119 - 4119]	1893 [-2832 - 3700]	3152 [-1886 - 14228]
Armenia	ARM	944 [535 - 1461]	83 [16 - 243]	3 [0 - 8]	77 [13 - 240]	95 [18 - 278]	228 [228 - 228]	133 [-51 - 210]	3335 [-465 - 13578]
Azerbaijan	AZE	3874 [2199 - 5993]	339 [66 - 998]	11 [2 - 33]	315 [54 - 976]	291 [58 - 823]	972 [972 - 972]	681 [148 - 914]	4114 [197 - 15828]
Burundi	BDI	9565 [5459 - 14727]	837 [162 - 2457]	26 [4 - 81]	736 [126 - 2275]	83 [14 - 252]	2396 [2396 - 2396]	2313 [2143 - 2381]	5415 [1010 - 18586]
Benin	BEN	9283 [5293 - 14306]	812 [157 - 2386]	25 [4 - 79]	724 [124 - 2241]	153 [32 - 453]	2261 [2261 - 2261]	2109 [1808 - 2230]	5066 [886 - 17383]
Burkina Faso	BFA	18003 [10256 - 27762]	1575 [305 - 4628]	49 [8 - 153]	1404 [240 - 4343]	344 [61 - 1067]	4197 [4197 - 4197]	3853 [3130 - 4136]	4770 [823 - 16577]
Bangladesh	BGD	68181 [38711 - 105466]	5964 [1155 - 17564]	185 [29 - 581]	5582 [948 - 17282]	1281 [369 - 3344]	18043 [18043 - 18043]	16762 [14700 - 17675]	5289 [876 - 18455]
Bolivia	BOL	5267 [2996 - 8134]	461 [89 - 1355]	14 [2 - 45]	427 [73 - 1320]	424 [84 - 1196]	1449 [1449 - 1449]	1025 [253 - 1365]	4449 [273 - 16800]
Bhutan	BTN	249 [141 - 385]	22 [4 - 64]	1 [0 - 2]	20 [3 - 63]	22 [4 - 64]	86 [86 - 86]	65 [22 - 82]	5811 [503 - 20727]
Central African Republic	CAF	2179 [1245 - 3351]	191 [37 - 559]	6 [1 - 19]	164 [28 - 506]	24 [5 - 66]	559 [559 - 559]	535 [493 - 554]	5646 [1013 - 19473]
Côte d'Ivoire	CIV	18163 [10348 - 28008]	1589 [308 - 4668]	49 [8 - 155]	1364 [233 - 4217]	499 [107 - 1459]	4877 [4877 - 4877]	4378 [3417 - 4770]	5611 [893 - 19519]
Cameroon	CMR	14647 [8347 - 22582]	1281 [248 - 3764]	40 [6 - 125]	1133 [194 - 3506]	361 [72 - 1028]	3475 [3475 - 3475]	3115 [2447 - 3403]	4836 [764 - 16794]
Democratic Republic of the Congo	COD	69197 [39466 - 106603]	6053 [1174 - 17781]	188 [30 - 589]	5392 [921 - 16675]	572 [107 - 1703]	17835 [17835 - 17835]	17263 [16132 - 17728]	5519 [1023 - 18811]
Congo	COG	3584 [2038 - 5538]	314 [61 - 923]	10 [2 - 31]	285 [49 - 882]	163 [33 - 461]	874 [874 - 874]	711 [413 - 841]	4469 [595 - 16169]
Comoros	COM	641 [365 - 989]	56 [11 - 165]	2 [0 - 5]	51 [9 - 156]	13 [3 - 40]	142 [142 - 142]	129 [102 - 139]	4459 [761 - 15487]
Djibouti	DJI	460 [262 - 709]	40 [8 - 118]	1 [0 - 4]	36 [6 - 111]	22 [4 - 63]	112 [112 - 112]	89 [49 - 107]	4452 [555 - 15924]

Eritrea	ERI	3848 [2186 - 5948]	337 [65 - 991]	10 [2 - 33]	306 [52 - 948]	33 [10 - 80]	920 [920 - 920]	887 [839 - 910]	5002 [918 - 17163]
Ethiopia	ETH	74378 [42292 - 114899]	6506 [1260 - 19145]	202 [32 - 633]	5936 [1011 - 18361]	829 [171 - 2398]	17699 [17699 - 17699]	16870 [15301 - 17528]	4934 [893 - 16984]
Georgia	GEO	1265 [717 - 1959]	111 [21 - 326]	3 [1 - 11]	103 [18 - 320]	109 [22 - 308]	305 [305 - 305]	196 [-3 - 283]	3592 [-20 - 13855]
Ghana	GHA	18496 [10520 - 28565]	1618 [313 - 4759]	50 [8 - 158]	1453 [248 - 4491]	382 [70 - 1136]	4847 [4847 - 4847]	4465 [3710 - 4776]	5368 [922 - 18882]
Guinea	GIN	9661 [5503 - 14901]	845 [164 - 2484]	26 [4 - 82]	756 [129 - 2339]	90 [14 - 349]	2256 [2256 - 2256]	2165 [1906 - 2242]	4987 [878 - 17264]
Gambia	GMB	1963 [1117 - 3029]	172 [33 - 505]	5 [1 - 17]	153 [26 - 472]	52 [11 - 152]	467 [467 - 467]	415 [315 - 456]	4752 [793 - 16790]
Guinea-Bissau	GNB	1273 [727 - 1959]	111 [22 - 327]	3 [1 - 11]	98 [17 - 303]	24 [4 - 74]	307 [307 - 307]	283 [233 - 303]	5014 [868 - 17159]
Guyana	GUY	358 [204 - 554]	31 [6 - 92]	1 [0 - 3]	29 [5 - 88]	28 [5 - 81]	88 [88 - 88]	60 [7 - 83]	3902 [129 - 14256]
Honduras	HND	4511 [2562 - 6977]	395 [76 - 1162]	12 [2 - 38]	370 [63 - 1145]	386 [74 - 1148]	1192 [1192 - 1192]	806 [44 - 1118]	4043 [68 - 15311]
Haiti	HTI	3619 [2060 - 5586]	317 [61 - 931]	10 [2 - 31]	285 [49 - 882]	54 [10 - 156]	954 [954 - 954]	900 [798 - 944]	5482 [987 - 18892]
Indonesia	IDN	97289 [55212 - 150555]	8510 [1648 - 25070]	264 [42 - 829]	7825 [1331 - 24222]	5719 [888 - 18719]	26630 [26630 - 26630]	20911 [7911 - 25742]	4767 [506 - 17291]
India	IND	545986 [310222 - 844007]	47760 [9250 - 140587]	1481 [233 - 4651]	43988 [7483 - 136118]	16838 [2632 - 56286]	132743 [132743 - 132743]	115905 [76457 - 130111]	4597 [766 - 16054]
Kenya	KEN	30668 [17428 - 47400]	2683 [520 - 7896]	83 [13 - 261]	2452 [417 - 7587]	1122 [258 - 3187]	7865 [7865 - 7865]	6743 [4678 - 7607]	4868 [733 - 17389]
Kyrgyzstan	KGZ	3309 [1877 - 5122]	289 [56 - 853]	9 [1 - 28]	268 [45 - 828]	75 [15 - 220]	858 [858 - 858]	782 [638 - 843]	5124 [869 - 17866]
Cambodia	KHM	8187 [4647 - 12669]	716 [139 - 2110]	22 [3 - 70]	660 [112 - 2044]	283 [51 - 807]	2196 [2196 - 2196]	1913 [1389 - 2145]	5098 [805 - 17976]
Kiribati	KIR	41 [24 - 64]	4 [1 - 11]	0 [0 - 0]	3 [1 - 10]	4 [1 - 11]	15 [15 - 15]	11 [4 - 14]	Inf [574 - 14431]
Lao People's Democratic Republic	LAO	2175 [1236 - 3361]	190 [37 - 560]	6 [1 - 19]	174 [30 - 539]	81 [17 - 231]	732 [732 - 732]	652 [501 - 716]	6572 [1085 - 23087]
Liberia	LBR	3631 [2066 - 5606]	318 [62 - 934]	10 [2 - 31]	287 [49 - 886]	1 [0 - 2]	916 [916 - 916]	915 [914 - 916]	5488 [1032 - 18684]
Sri Lanka	LKA	5538 [3140 - 8579]	484 [94 - 1428]	15 [2 - 47]	456 [77 - 1412]	381 [78 - 1087]	1893 [1893 - 1893]	1512 [806 - 1815]	5978 [712 - 21810]
Lesotho	LSO	1020 [580 - 1575]	89 [17 - 262]	3 [0 - 9]	76 [13 - 235]	31 [6 - 89]	243 [243 - 243]	213 [154 - 237]	4894 [788 - 16999]
Republic of Moldova	MDA	927 [526 - 1435]	81 [16 - 239]	3 [0 - 8]	75 [13 - 232]	55 [11 - 163]	240 [240 - 240]	185 [76 - 229]	4427 [486 - 16176]

Madagascar	MDG	18508 [10516 - 28608]	1619 [314 - 4766]	50 [8 - 158]	1477 [251 - 4570]	165 [31 - 487]	4674 [4674 - 4674]	4509 [4187 - 4643]	5279 [976 - 18302]
Mali	MLI	16812 [9592 - 25891]	1471 [285 - 4320]	46 [7 - 143]	1301 [222 - 4021]	228 [49 - 645]	4050 [4050 - 4050]	3822 [3405 - 4001]	5099 [915 - 17417]
Myanmar	MMR	14182 [8062 - 21915]	1241 [240 - 3651]	38 [6 - 121]	1129 [192 - 3494]	183 [33 - 582]	4880 [4880 - 4880]	4697 [4298 - 4847]	7189 [1336 - 24773]
Mongolia	MNG	1130 [641 - 1749]	99 [19 - 291]	3 [0 - 10]	91 [15 - 281]	67 [13 - 189]	418 [418 - 418]	352 [229 - 405]	6857 [1027 - 24421]
Mozambique	MOZ	27378 [15608 - 42194]	2395 [464 - 7035]	74 [12 - 233]	2118 [362 - 6550]	307 [57 - 912]	6532 [6532 - 6532]	6225 [5620 - 6475]	5083 [936 - 17458]
Mauritania	MRT	3385 [1928 - 5220]	296 [57 - 870]	9 [1 - 29]	266 [45 - 824]	80 [17 - 230]	846 [846 - 846]	766 [616 - 829]	5037 [829 - 17518]
Malawi	MWI	14423 [8210 - 22260]	1262 [245 - 3709]	39 [6 - 123]	1139 [194 - 3523]	156 [27 - 493]	3410 [3410 - 3410]	3254 [2917 - 3383]	4937 [918 - 17026]
Niger	NER	24184 [13795 - 37252]	2115 [410 - 6215]	66 [10 - 206]	1887 [322 - 5836]	244 [40 - 785]	5805 [5805 - 5805]	5561 [5021 - 5765]	5087 [932 - 17449]
Nigeria	NGA	94220 [53758 - 145104]	8242 [1598 - 24209]	256 [40 - 802]	7079 [1212 - 21876]	2721 [550 - 7876]	23721 [23721 - 23721]	21000 [15845 - 23171]	5218 [842 - 18026]
Nicaragua	NIC	2764 [1568 - 4279]	242 [47 - 712]	8 [1 - 24]	228 [39 - 706]	175 [37 - 485]	718 [718 - 718]	543 [233 - 681]	4309 [468 - 15831]
Nepal	NPL	12750 [7238 - 19724]	1115 [216 - 3285]	35 [5 - 109]	1035 [176 - 3203]	195 [37 - 562]	3207 [3207 - 3207]	3011 [2645 - 3169]	5057 [910 - 17417]
Pakistan	PAK	124442 [70859 - 191996]	10885 [2110 - 31992]	338 [53 - 1060]	9944 [1694 - 30756]	4658 [690 - 17192]	27158 [27158 - 27158]	22500 [9966 - 26469]	3997 [495 - 14220]
Papua New Guinea	PNG	2629 [1495 - 4061]	230 [45 - 677]	7 [1 - 22]	209 [36 - 645]	200 [36 - 644]	961 [961 - 961]	760 [317 - 925]	6495 [744 - 23732]
Democratic People's Republic of Korea	PRK	7806 [4428 - 12084]	683 [132 - 2012]	21 [3 - 66]	633 [108 - 1961]	76 [14 - 241]	2089 [2089 - 2089]	2013 [1848 - 2075]	5492 [1012 - 18803]
Rwanda	RWA	7992 [4543 - 12350]	699 [135 - 2058]	22 [3 - 68]	640 [109 - 1981]	145 [28 - 441]	2182 [2182 - 2182]	2037 [1740 - 2154]	5528 [985 - 19061]
Sudan	SDN	30436 [17318 - 46987]	2662 [516 - 7829]	83 [13 - 259]	2409 [411 - 7449]	1593 [283 - 4575]	7418 [7418 - 7418]	5825 [2843 - 7135]	4344 [514 - 15520]
Senegal	SEN	15198 [8634 - 23495]	1329 [257 - 3913]	41 [6 - 129]	1217 [207 - 3766]	428 [87 - 1285]	3190 [3190 - 3190]	2762 [1905 - 3103]	4004 [606 - 14285]
Solomon Islands	SLB	239 [136 - 370]	21 [4 - 62]	1 [0 - 2]	19 [3 - 60]	29 [6 - 86]	83 [83 - 83]	53 [-3 - 77]	5129 [-124 - 19445]
Sierra Leone	SLE	5313 [3034 - 8176]	465 [90 - 1364]	14 [2 - 45]	394 [68 - 1216]	106 [19 - 330]	1339 [1339 - 1339]	1233 [1009 - 1320]	5414 [958 - 18630]
Somalia	SOM	7245 [4136 - 11151]	634 [123 - 1860]	20 [3 - 62]	555 [95 - 1714]	32 [9 - 85]	2090 [2090 - 2090]	2058 [2005 - 2081]	6388 [1184 - 21785]

South Sudan	SSD	5288 [3017 - 8144]	463 [90 - 1359]	14 [2 - 45]	406 [69 - 1256]	273 [50 - 801]	1322 [1322 - 1322]	1049 [521 - 1273]	4621 [557 - 16812]
Sao Tome and Principe	STP	137 [78 - 211]	12 [2 - 35]	0 [0 - 1]	11 [2 - 34]	7 [1 - 20]	37 [37 - 37]	30 [17 - 35]	4896 [682 - 17159]
Chad	TCD	10729 [6133 - 16495]	938 [182 - 2752]	29 [5 - 91]	802 [137 - 2475]	141 [28 - 420]	2625 [2625 - 2625]	2484 [2204 - 2596]	5372 [961 - 18482]
Togo	TGO	4954 [2820 - 7643]	433 [84 - 1274]	13 [2 - 42]	384 [66 - 1187]	73 [14 - 209]	1188 [1188 - 1188]	1115 [979 - 1174]	5032 [915 - 17309]
Tajikistan	TJK	5624 [3195 - 8695]	492 [95 - 1448]	15 [2 - 48]	457 [78 - 1414]	136 [26 - 396]	1468 [1468 - 1468]	1332 [1072 - 1442]	5096 [870 - 17617]
Timor-Leste	TLS	777 [441 - 1200]	68 [13 - 200]	2 [0 - 7]	63 [11 - 194]	39 [7 - 119]	253 [253 - 253]	215 [135 - 247]	6036 [923 - 21157]
United Republic of Tanzania	TZA	52297 [29733 - 80797]	4575 [886 - 13461]	142 [22 - 445]	4177 [711 - 12923]	695 [130 - 2000]	12729 [12729 - 12729]	12034 [10729 - 12599]	5009 [900 - 17219]
Uganda	UGA	41067 [23393 - 63339]	3592 [696 - 10557]	111 [18 - 350]	3187 [544 - 9855]	908 [165 - 2843]	9778 [9778 - 9778]	8870 [6935 - 9614]	4842 [841 - 16771]
Ukraine	UKR	8284 [4696 - 12831]	725 [140 - 2136]	22 [4 - 71]	672 [114 - 2080]	820 [148 - 2514]	2140 [2140 - 2140]	1320 [-374 - 1992]	3708 [-500 - 14997]
Uzbekistan	UZB	14922 [8471 - 23085]	1305 [253 - 3844]	40 [6 - 127]	1211 [206 - 3747]	1045 [202 - 3081]	3884 [3884 - 3884]	2839 [803 - 3683]	4277 [329 - 15791]
Viet Nam	VNM	23357 [13251 - 36156]	2043 [396 - 6020]	63 [10 - 199]	1930 [328 - 5978]	222 [70 - 520]	9059 [9059 - 9059]	8837 [8539 - 8990]	7927 [1453 - 27285]
Yemen	YEM	15419 [8767 - 23822]	1349 [261 - 3969]	42 [7 - 131]	1221 [208 - 3777]	636 [137 - 1788]	3730 [3730 - 3730]	3093 [1942 - 3593]	4540 [630 - 16620]
Zambia	ZMB	15795 [8988 - 24384]	1382 [268 - 4063]	43 [7 - 135]	1237 [211 - 3825]	846 [220 - 2312]	3783 [3783 - 3783]	2937 [1471 - 3562]	4278 [510 - 15314]
Zimbabwe	ZWE	11180 [6358 - 17270]	978 [189 - 2877]	30 [5 - 95]	869 [148 - 2688]	225 [44 - 673]	2960 [2960 - 2960]	2735 [2287 - 2916]	5470 [955 - 19086]
Maternal vs. no intervention									
Afghanistan	AFG	15386 [8319 - 24952]	1347 [252 - 4009]	44 [7 - 139]	1271 [209 - 3981]	121 [22 - 371]	2987 [2987 - 2987]	2867 [2617 - 2966]	3989 [702 - 13972]
Angola	AGO	10921 [5913 - 17696]	956 [179 - 2844]	31 [5 - 98]	892 [147 - 2795]	1535 [282 - 4870]	2117 [2117 - 2117]	582 [-2753 - 1835]	1708 [-3054 - 9512]
Armenia	ARM	649 [350 - 1056]	57 [11 - 169]	2 [0 - 6]	56 [9 - 175]	65 [12 - 194]	115 [115 - 115]	50 [-79 - 104]	1992 [-1307 - 9434]
Azerbaijan	AZE	2667 [1439 - 4334]	233 [44 - 695]	8 [1 - 24]	226 [37 - 710]	200 [38 - 578]	494 [494 - 494]	294 [-84 - 456]	2692 [-173 - 11244]
Burundi	BDI	6596 [3576 - 10679]	577 [108 - 1716]	19 [3 - 59]	530 [87 - 1658]	57 [10 - 180]	1231 [1231 - 1231]	1174 [1051 - 1221]	3915 [687 - 13594]
Benin	BEN	6400 [3467 - 10368]	560 [105 - 1666]	18 [3 - 58]	521 [86 - 1632]	105 [22 - 317]	1166 [1166 - 1166]	1061 [849 - 1144]	3646 [581 - 12717]

Burkina Faso	BFA	12405 [6714 - 20105]	1086 [203 - 3232]	35 [5 - 112]	1010 [166 - 3163]	237 [41 - 748]	2144 [2144 - 2144]	1907 [1396 - 2103]	3380 [536 - 12032]
Bangladesh	BGD	46923 [25316 - 76271]	4107 [767 - 12226]	133 [20 - 423]	4011 [655 - 12579]	882 [244 - 2382]	9257 [9257 - 9257]	8374 [6875 - 9012]	3798 [559 - 13609]
Bolivia	BOL	3626 [1960 - 5884]	317 [59 - 945]	10 [2 - 33]	307 [50 - 962]	292 [56 - 829]	734 [734 - 734]	442 [-95 - 678]	2868 [-179 - 11834]
Bhutan	BTN	171 [92 - 279]	15 [3 - 45]	0 [0 - 2]	15 [2 - 46]	15 [3 - 46]	44 [44 - 44]	29 [-2 - 41]	3865 [-51 - 14507]
Central African Republic	CAF	1504 [816 - 2432]	132 [25 - 391]	4 [1 - 14]	118 [19 - 369]	17 [3 - 47]	290 [290 - 290]	273 [242 - 286]	4112 [689 - 14375]
Côte d'Ivoire	CIV	12518 [6776 - 20286]	1096 [205 - 3261]	36 [5 - 113]	981 [162 - 3071]	344 [71 - 1015]	2505 [2505 - 2505]	2161 [1490 - 2434]	3979 [561 - 14163]
Cameroon	CMR	10096 [5467 - 16360]	884 [165 - 2630]	29 [4 - 91]	815 [134 - 2553]	249 [48 - 713]	1772 [1772 - 1772]	1524 [1060 - 1724]	3405 [468 - 12218]
Democratic Republic of the Congo	COD	47712 [25853 - 77274]	4176 [782 - 12419]	135 [21 - 430]	3879 [637 - 12147]	394 [71 - 1187]	9168 [9168 - 9168]	8774 [7981 - 9097]	3997 [692 - 13962]
Congo	COG	2469 [1334 - 4007]	216 [40 - 643]	7 [1 - 22]	205 [34 - 642]	112 [22 - 320]	444 [444 - 444]	332 [124 - 422]	3037 [263 - 11552]
Comoros	COM	442 [239 - 716]	39 [7 - 115]	1 [0 - 4]	36 [6 - 114]	9 [2 - 28]	73 [73 - 73]	64 [46 - 71]	3180 [474 - 11495]
Djibouti	DJI	317 [171 - 514]	28 [5 - 83]	1 [0 - 3]	26 [4 - 81]	15 [3 - 44]	58 [58 - 58]	42 [13 - 55]	3086 [223 - 11781]
Eritrea	ERI	2649 [1430 - 4302]	232 [43 - 690]	8 [1 - 24]	220 [36 - 690]	23 [7 - 56]	470 [470 - 470]	448 [415 - 464]	3604 [623 - 12546]
Ethiopia	ETH	51208 [27666 - 83117]	4482 [838 - 13342]	145 [22 - 461]	4266 [699 - 13371]	571 [114 - 1666]	9121 [9121 - 9121]	8549 [7455 - 9006]	3574 [597 - 12446]
Georgia	GEO	870 [469 - 1416]	76 [14 - 227]	2 [0 - 8]	74 [12 - 233]	75 [15 - 213]	154 [154 - 154]	79 [-59 - 139]	2226 [-621 - 9633]
Ghana	GHA	12738 [6883 - 20668]	1115 [208 - 3319]	36 [5 - 115]	1044 [171 - 3272]	263 [48 - 797]	2480 [2480 - 2480]	2216 [1683 - 2432]	3821 [600 - 13721]
Guinea	GIN	6655 [3602 - 10788]	583 [109 - 1734]	19 [3 - 60]	544 [89 - 1703]	62 [9 - 243]	1152 [1152 - 1152]	1090 [909 - 1143]	3591 [574 - 12682]
Gambia	GMB	1352 [731 - 2193]	118 [22 - 352]	4 [1 - 12]	110 [18 - 344]	36 [7 - 106]	239 [239 - 239]	203 [133 - 232]	3344 [500 - 12001]
Guinea-Bissau	GNB	878 [476 - 1421]	77 [14 - 228]	2 [0 - 8]	71 [12 - 221]	17 [3 - 51]	159 [159 - 159]	143 [108 - 156]	3616 [573 - 12693]
Guyana	GUY	247 [133 - 401]	22 [4 - 64]	1 [0 - 2]	20 [3 - 64]	19 [4 - 57]	45 [45 - 45]	26 [-12 - 41]	2488 [-443 - 10211]
Honduras	HND	3105 [1675 - 5046]	272 [51 - 809]	9 [1 - 28]	266 [43 - 834]	266 [50 - 800]	604 [604 - 604]	338 [-196 - 553]	2549 [-567 - 10639]

Haiti	HTI	2493 [1348 - 4043]	218 [41 - 650]	7 [1 - 22]	205 [34 - 643]	37 [7 - 109]	489 [489 - 489]	452 [380 - 482]	3938 [654 - 13848]
Indonesia	IDN	66946 [36100 - 108869]	5860 [1094 - 17443]	190 [29 - 603]	5622 [920 - 17628]	3936 [594 - 13015]	13493 [13493 - 13493]	9557 [478 - 12899]	3176 [53 - 12263]
India	IND	375922 [202966 - 610538]	32904 [6149 - 97946]	1066 [162 - 3388]	31613 [5175 - 99111]	11598 [1760 - 38593]	67934 [67934 - 67934]	56336 [29341 - 66174]	3208 [445 - 11573]
Kenya	KEN	21117 [11403 - 34291]	1848 [345 - 5502]	60 [9 - 190]	1762 [289 - 5525]	773 [171 - 2231]	4023 [4023 - 4023]	3251 [1792 - 3852]	3391 [425 - 12478]
Kyrgyzstan	KGZ	2276 [1227 - 3703]	199 [37 - 593]	6 [1 - 21]	192 [31 - 603]	52 [10 - 153]	433 [433 - 433]	381 [280 - 423]	3588 [534 - 12826]
Cambodia	KHM	5633 [3038 - 9160]	493 [92 - 1468]	16 [2 - 51]	474 [78 - 1488]	195 [35 - 566]	1111 [1111 - 1111]	917 [545 - 1077]	3522 [463 - 12836]
Kiribati	KIR	29 [15 - 46]	2 [0 - 7]	0 [0 - 0]	2 [0 - 7]	3 [0 - 8]	8 [8 - 8]	5 [0 - 7]	Inf [-55 - Inf]
Lao People's Democratic Republic	LAO	1498 [809 - 2431]	131 [25 - 390]	4 [1 - 13]	125 [21 - 392]	55 [11 - 162]	375 [375 - 375]	320 [213 - 364]	4633 [677 - 16788]
Liberia	LBR	2501 [1352 - 4057]	219 [41 - 652]	7 [1 - 23]	206 [34 - 646]	0 [0 - 1]	468 [468 - 468]	467 [466 - 468]	3988 [724 - 13757]
Sri Lanka	LKA	3809 [2051 - 6201]	333 [62 - 992]	11 [2 - 34]	327 [53 - 1027]	262 [51 - 757]	951 [951 - 951]	689 [195 - 900]	3990 [253 - 15376]
Lesotho	LSO	703 [380 - 1140]	62 [12 - 183]	2 [0 - 6]	55 [9 - 171]	21 [4 - 62]	124 [124 - 124]	103 [62 - 120]	3414 [459 - 12516]
Republic of Moldova	MDA	637 [343 - 1037]	56 [10 - 166]	2 [0 - 6]	54 [9 - 169]	38 [7 - 114]	121 [121 - 121]	83 [7 - 114]	2919 [68 - 11422]
Madagascar	MDG	12739 [6877 - 20691]	1115 [208 - 3319]	36 [5 - 115]	1061 [174 - 3327]	114 [21 - 343]	2380 [2380 - 2380]	2266 [2037 - 2360]	3788 [652 - 13332]
Mali	MLI	11591 [6282 - 18767]	1014 [190 - 3016]	33 [5 - 105]	935 [154 - 2929]	157 [32 - 449]	2093 [2093 - 2093]	1936 [1644 - 2061]	3691 [617 - 13037]
Myanmar	MMR	9769 [5278 - 15857]	855 [160 - 2545]	28 [4 - 88]	812 [133 - 2545]	126 [21 - 397]	2490 [2490 - 2490]	2364 [2092 - 2468]	5160 [895 - 18130]
Mongolia	MNG	778 [419 - 1265]	68 [13 - 203]	2 [0 - 7]	65 [11 - 205]	46 [9 - 132]	211 [211 - 211]	165 [79 - 202]	4651 [531 - 17283]
Mozambique	MOZ	18877 [10225 - 30580]	1652 [309 - 4915]	54 [8 - 170]	1523 [250 - 4770]	212 [39 - 642]	3330 [3330 - 3330]	3117 [2687 - 3291]	3634 [622 - 12776]
Mauritania	MRT	2334 [1264 - 3783]	204 [38 - 608]	7 [1 - 21]	192 [31 - 600]	55 [11 - 159]	435 [435 - 435]	380 [276 - 424]	3581 [522 - 12919]
Malawi	MWI	9943 [5378 - 16122]	870 [163 - 2591]	28 [4 - 90]	820 [135 - 2568]	107 [18 - 343]	1743 [1743 - 1743]	1635 [1400 - 1725]	3539 [606 - 12298]
Niger	NER	16667 [9031 - 26993]	1459 [273 - 4338]	47 [7 - 150]	1357 [223 - 4250]	168 [27 - 540]	3013 [3013 - 3013]	2845 [2473 - 2986]	3711 [640 - 13010]
Nigeria	NGA	64955 [35207 - 105181]	5685 [1065 - 16903]	184 [28 - 586]	5091 [839 - 15935]	1877 [366 - 5545]	12392 [12392 - 12392]	10516 [6847 - 12026]	3760 [525 - 13382]

Nicaragua	NIC	1902 [1025 - 3094]	166 [31 - 495]	5 [1 - 17]	164 [27 - 514]	120 [25 - 335]	362 [362 - 362]	241 [26 - 337]	2816 [89 - 11096]
Nepal	NPL	8775 [4734 - 14264]	768 [143 - 2286]	25 [4 - 79]	743 [122 - 2331]	134 [25 - 393]	1633 [1633 - 1633]	1499 [1240 - 1608]	3601 [602 - 12797]
Pakistan	PAK	85805 [46430 - 139098]	7510 [1405 - 22357]	243 [37 - 773]	7153 [1173 - 22415]	3213 [451 - 11772]	14191 [14191 - 14191]	10978 [2419 - 13740]	2815 [193 - 10835]
Papua New Guinea	PNG	1811 [979 - 2939]	159 [30 - 472]	5 [1 - 16]	150 [25 - 470]	138 [24 - 444]	488 [488 - 488]	350 [44 - 464]	4352 [187 - 16926]
Democratic People's Republic of Korea	PRK	5369 [2894 - 8736]	470 [88 - 1399]	15 [2 - 48]	455 [74 - 1427]	52 [9 - 166]	1059 [1059 - 1059]	1007 [893 - 1050]	3919 [678 - 13755]
Rwanda	RWA	5504 [2973 - 8935]	482 [90 - 1434]	16 [2 - 50]	460 [75 - 1443]	100 [19 - 310]	1110 [1110 - 1110]	1010 [800 - 1091]	3922 [645 - 13804]
Sudan	SDN	20966 [11335 - 34009]	1835 [343 - 5463]	59 [9 - 189]	1732 [284 - 5428]	1098 [190 - 3210]	3802 [3802 - 3802]	2704 [592 - 3612]	2945 [152 - 11259]
Senegal	SEN	10462 [5648 - 16994]	916 [171 - 2726]	30 [5 - 94]	875 [143 - 2742]	294 [58 - 896]	1635 [1635 - 1635]	1341 [739 - 1577]	2802 [358 - 10338]
Solomon Islands	SLB	165 [89 - 268]	14 [3 - 43]	0 [0 - 1]	14 [2 - 44]	20 [4 - 60]	42 [42 - 42]	22 [-18 - 38]	3206 [-1328 - 14425]
Sierra Leone	SLE	3665 [1989 - 5932]	321 [60 - 953]	10 [2 - 33]	283 [47 - 886]	73 [13 - 231]	686 [686 - 686]	613 [455 - 674]	3849 [624 - 13691]
Somalia	SOM	4997 [2710 - 8087]	437 [82 - 1299]	14 [2 - 45]	399 [66 - 1249]	22 [6 - 60]	1083 [1083 - 1083]	1061 [1024 - 1077]	4691 [824 - 16300]
South Sudan	SSD	3646 [1976 - 5904]	319 [60 - 949]	10 [2 - 33]	292 [48 - 915]	188 [33 - 566]	682 [682 - 682]	493 [116 - 648]	3165 [211 - 12053]
Sao Tome and Principe	STP	94 [51 - 153]	8 [2 - 25]	0 [0 - 1]	8 [1 - 25]	5 [1 - 14]	19 [19 - 19]	14 [5 - 18]	Inf [308 - 15869]
Chad	TCD	7403 [4020 - 11971]	648 [122 - 1923]	21 [3 - 67]	577 [95 - 1805]	97 [19 - 293]	1367 [1367 - 1367]	1270 [1074 - 1348]	3922 [643 - 13832]
Togo	TGO	3413 [1847 - 5534]	299 [56 - 889]	10 [1 - 31]	276 [45 - 865]	50 [9 - 147]	615 [615 - 615]	565 [468 - 605]	3642 [613 - 12845]
Tajikistan	TJK	3872 [2090 - 6289]	339 [63 - 1009]	11 [2 - 35]	328 [54 - 1029]	93 [17 - 278]	744 [744 - 744]	651 [466 - 727]	3573 [548 - 12768]
Timor-Leste	TLS	535 [289 - 868]	47 [9 - 139]	2 [0 - 5]	45 [7 - 141]	27 [4 - 83]	129 [129 - 129]	102 [46 - 125]	4156 [486 - 14997]
United Republic of Tanzania	TZA	36017 [19457 - 58458]	3153 [589 - 9384]	102 [16 - 325]	3003 [492 - 9412]	479 [88 - 1390]	6511 [6511 - 6511]	6032 [5120 - 6423]	3591 [592 - 12641]
Uganda	UGA	28304 [15319 - 45876]	2477 [464 - 7374]	80 [12 - 255]	2292 [377 - 7178]	626 [111 - 2043]	4994 [4994 - 4994]	4368 [2951 - 4883]	3415 [529 - 12133]
Ukraine	UKR	5697 [3068 - 9275]	499 [93 - 1484]	16 [2 - 51]	482 [79 - 1513]	564 [98 - 1748]	1080 [1080 - 1080]	515 [-669 - 981]	2231 [-1393 - 10453]

Uzbekistan	UZB	10271 [5541 - 16696]	899 [168 - 2676]	29 [4 - 93]	870 [142 - 2728]	719 [136 - 2171]	1966 [1966 - 1966]	1246 [-206 - 1830]	2779 [-162 - 11006]
Viet Nam	VNM	16069 [8662 - 26141]	1407 [263 - 4187]	46 [7 - 145]	1387 [226 - 4350]	153 [47 - 366]	4576 [4576 - 4576]	4423 [4210 - 4529]	5663 [984 - 19953]
Yemen	YEM	10621 [5738 - 17236]	930 [174 - 2767]	30 [5 - 96]	878 [144 - 2751]	439 [91 - 1259]	1920 [1920 - 1920]	1482 [661 - 1829]	3162 [329 - 11974]
Zambia	ZMB	10881 [5883 - 17648]	952 [178 - 2835]	31 [5 - 98]	889 [146 - 2787]	583 [146 - 1622]	1932 [1932 - 1932]	1349 [310 - 1786]	2877 [163 - 10872]
Zimbabwe	ZWE	7701 [4162 - 12496]	674 [126 - 2006]	22 [3 - 69]	625 [103 - 1958]	155 [29 - 470]	1511 [1511 - 1511]	1356 [1040 - 1481]	3884 [619 - 13908]
mAb vs. Maternal									
Afghanistan	AFG	6943 [4474 - 9810]	606 [124 - 1698]	17 [3 - 51]	497 [93 - 1476]	55 [11 - 167]	2828 [2828 - 2828]	2773 [2661 - 2817]	9248 [1859 - 30267]
Angola	AGO	4921 [3176 - 6943]	430 [88 - 1202]	12 [2 - 36]	348 [65 - 1035]	691 [134 - 2101]	2002 [2002 - 2002]	1311 [-100 - 1868]	6704 [-176 - 24849]
Armenia	ARM	295 [189 - 418]	26 [5 - 72]	1 [0 - 2]	22 [4 - 65]	30 [6 - 85]	112 [112 - 112]	83 [28 - 106]	6656 [640 - 24639]
Azerbaijan	AZE	1207 [776 - 1710]	105 [22 - 295]	3 [0 - 9]	89 [16 - 264]	90 [19 - 252]	478 [478 - 478]	387 [225 - 459]	7604 [1006 - 26767]
Burundi	BDI	2969 [1919 - 4183]	259 [53 - 725]	7 [1 - 22]	207 [39 - 614]	26 [5 - 77]	1165 [1165 - 1165]	1139 [1088 - 1160]	9120 [1843 - 29538]
Benin	BEN	2883 [1862 - 4066]	252 [51 - 704]	7 [1 - 21]	203 [38 - 604]	47 [10 - 135]	1095 [1095 - 1095]	1048 [960 - 1085]	8572 [1676 - 28264]
Burkina Faso	BFA	5597 [3610 - 7901]	489 [100 - 1368]	14 [2 - 41]	395 [74 - 1173]	107 [20 - 321]	2053 [2053 - 2053]	1946 [1732 - 2033]	8196 [1630 - 26939]
Bangladesh	BGD	21257 [13667 - 30107]	1857 [379 - 5202]	52 [9 - 157]	1572 [292 - 4680]	399 [120 - 987]	8786 [8786 - 8786]	8387 [7800 - 8667]	8953 [1690 - 29706]
Bolivia	BOL	1641 [1056 - 2320]	143 [29 - 401]	4 [1 - 12]	120 [22 - 357]	132 [27 - 367]	715 [715 - 715]	583 [348 - 688]	8331 [1200 - 28271]
Bhutan	BTN	78 [50 - 110]	7 [1 - 19]	0 [0 - 1]	6 [1 - 17]	7 [1 - 19]	42 [42 - 42]	36 [23 - 41]	Inf [1709 - 40046]
Central African Republic	CAF	675 [437 - 951]	59 [12 - 165]	2 [0 - 5]	46 [9 - 136]	7 [2 - 20]	270 [270 - 270]	262 [250 - 268]	9441 [1862 - 29823]
Côte d'Ivoire	CIV	5645 [3642 - 7967]	493 [101 - 1379]	14 [2 - 42]	383 [72 - 1138]	155 [35 - 437]	2371 [2371 - 2371]	2217 [1934 - 2336]	9633 [1826 - 31767]
Cameroon	CMR	4551 [2937 - 6421]	398 [81 - 1111]	11 [2 - 34]	318 [60 - 945]	112 [23 - 313]	1703 [1703 - 1703]	1591 [1390 - 1680]	8363 [1558 - 27710]
Democratic Republic of the Congo	COD	21485 [13881 - 30291]	1877 [384 - 5244]	52 [9 - 158]	1514 [283 - 4499]	178 [35 - 518]	8667 [8667 - 8667]	8490 [8149 - 8632]	9278 [1878 - 30224]
Congo	COG	1116 [719 - 1579]	97 [20 - 273]	3 [0 - 8]	80 [15 - 239]	51 [11 - 144]	430 [430 - 430]	380 [286 - 420]	7989 [1433 - 26875]

Comoros	COM	199 [128 - 281]	17 [4 - 49]	0 [0 - 2]	14 [3 - 42]	4 [1 - 12]	69 [69 - 69]	65 [57 - 68]	7697 [1470 - 22663]
Djibouti	DJI	143 [92 - 202]	12 [3 - 35]	0 [0 - 1]	10 [2 - 30]	7 [1 - 19]	54 [54 - 54]	47 [35 - 52]	Inf [1364 - 25719]
Eritrea	ERI	1199 [772 - 1697]	105 [21 - 294]	3 [0 - 9]	86 [16 - 256]	10 [3 - 24]	449 [449 - 449]	439 [425 - 446]	8444 [1693 - 27829]
Ethiopia	ETH	23171 [14917 - 32774]	2024 [413 - 5669]	57 [10 - 171]	1670 [312 - 4964]	258 [56 - 728]	8579 [8579 - 8579]	8321 [7851 - 8523]	8281 [1636 - 27108]
Georgia	GEO	395 [254 - 560]	34 [7 - 97]	1 [0 - 3]	29 [5 - 86]	34 [7 - 92]	151 [151 - 151]	117 [59 - 144]	6947 [916 - 23984]
Ghana	GHA	5757 [3707 - 8141]	503 [103 - 1409]	14 [2 - 42]	408 [76 - 1214]	119 [23 - 346]	2367 [2367 - 2367]	2248 [2021 - 2344]	9173 [1784 - 30212]
Guinea	GIN	3006 [1939 - 4244]	263 [54 - 734]	7 [1 - 22]	213 [40 - 632]	28 [4 - 107]	1104 [1104 - 1104]	1075 [997 - 1099]	8426 [1639 - 27512]
Gambia	GMB	611 [393 - 863]	53 [11 - 149]	1 [0 - 5]	43 [8 - 127]	16 [4 - 46]	228 [228 - 228]	212 [182 - 224]	8225 [1595 - 27567]
Guinea-Bissau	GNB	395 [256 - 556]	35 [7 - 96]	1 [0 - 3]	28 [5 - 82]	8 [1 - 23]	148 [148 - 148]	140 [125 - 146]	8496 [1664 - 28702]
Guyana	GUY	112 [72 - 158]	10 [2 - 27]	0 [0 - 1]	8 [1 - 24]	9 [2 - 25]	43 [43 - 43]	35 [18 - 42]	Inf [1057 - 30980]
Honduras	HND	1407 [904 - 1992]	123 [25 - 344]	3 [1 - 10]	104 [19 - 310]	120 [25 - 341]	588 [588 - 588]	468 [247 - 564]	7709 [1071 - 27121]
Haiti	HTI	1126 [726 - 1591]	98 [20 - 275]	3 [0 - 8]	80 [15 - 238]	17 [3 - 47]	465 [465 - 465]	448 [418 - 462]	9282 [1848 - 30418]
Indonesia	IDN	30342 [19498 - 42993]	2651 [541 - 7425]	74 [13 - 224]	2204 [410 - 6563]	1783 [293 - 5590]	13137 [13137 - 13137]	11354 [7546 - 12844]	8672 [1446 - 29332]
India	IND	170064 [109420 - 240709]	14856 [3030 - 41609]	415 [70 - 1254]	12375 [2304 - 36811]	5241 [865 - 17119]	64809 [64809 - 64809]	59568 [47690 - 63944]	8012 [1574 - 26597]
Kenya	KEN	9551 [6146 - 13516]	834 [170 - 2336]	23 [4 - 70]	690 [128 - 2051]	349 [85 - 942]	3842 [3842 - 3842]	3493 [2900 - 3758]	8500 [1565 - 28616]
Kyrgyzstan	KGZ	1032 [663 - 1463]	90 [18 - 253]	3 [0 - 8]	75 [14 - 224]	24 [5 - 67]	424 [424 - 424]	401 [358 - 420]	8897 [1729 - 29555]
Cambodia	KHM	2554 [1641 - 3619]	223 [46 - 625]	6 [1 - 19]	186 [35 - 554]	88 [17 - 248]	1085 [1085 - 1085]	997 [837 - 1068]	8967 [1706 - 29648]
Kiribati	KIR	13 [8 - 18]	1 [0 - 3]	0 [0 - 0]	1 [0 - 3]	1 [0 - 3]	7 [7 - 7]	6 [4 - 7]	NaN [1795 - Inf]
Lao People's Democratic Republic	LAO	677 [435 - 958]	59 [12 - 166]	2 [0 - 5]	49 [9 - 146]	25 [5 - 70]	357 [357 - 357]	332 [287 - 352]	11358 [2146 - 38175]
Liberia	LBR	1130 [728 - 1597]	99 [20 - 276]	3 [0 - 8]	81 [15 - 240]	0 [0 - 1]	448 [448 - 448]	448 [447 - 448]	9183 [1866 - 29848]
Sri Lanka	LKA	1730 [1110 - 2453]	151 [31 - 423]	4 [1 - 13]	129 [24 - 383]	119 [26 - 342]	942 [942 - 942]	823 [600 - 916]	10845 [1804 - 36357]

Lesotho	LSO	317 [205 - 448]	28 [6 - 78]	1 [0 - 2]	21 [4 - 64]	10 [2 - 27]	119 [119 - 119]	110 [92 - 117]	8565 [1622 - 28575]
Republic of Moldova	MDA	289 [186 - 410]	25 [5 - 71]	1 [0 - 2]	21 [4 - 63]	17 [3 - 49]	119 [119 - 119]	102 [70 - 116]	8147 [1399 - 27702]
Madagascar	MDG	5769 [3712 - 8167]	504 [103 - 1411]	14 [2 - 42]	416 [78 - 1237]	52 [10 - 146]	2294 [2294 - 2294]	2242 [2147 - 2283]	8944 [1784 - 29203]
Mali	MLI	5221 [3375 - 7359]	456 [93 - 1274]	13 [2 - 39]	365 [68 - 1086]	71 [16 - 195]	1957 [1957 - 1957]	1886 [1762 - 1941]	8575 [1696 - 28124]
Myanmar	MMR	4413 [2841 - 6243]	385 [79 - 1079]	11 [2 - 33]	317 [59 - 943]	57 [11 - 180]	2390 [2390 - 2390]	2333 [2210 - 2380]	12185 [2463 - 39539]
Mongolia	MNG	353 [226 - 500]	31 [6 - 86]	1 [0 - 3]	26 [5 - 76]	21 [4 - 58]	208 [208 - 208]	187 [150 - 203]	12312 [2271 - 40125]
Mozambique	MOZ	8501 [5489 - 11990]	743 [152 - 2076]	21 [4 - 63]	595 [111 - 1767]	95 [19 - 282]	3202 [3202 - 3202]	3107 [2921 - 3184]	8659 [1754 - 28261]
Mauritania	MRT	1051 [678 - 1483]	92 [19 - 257]	3 [0 - 8]	75 [14 - 222]	25 [5 - 70]	411 [411 - 411]	386 [341 - 406]	8633 [1666 - 28787]
Malawi	MWI	4480 [2888 - 6327]	391 [80 - 1095]	11 [2 - 33]	320 [60 - 950]	48 [9 - 150]	1667 [1667 - 1667]	1619 [1517 - 1658]	8387 [1691 - 27573]
Niger	NER	7517 [4857 - 10597]	657 [134 - 1835]	18 [3 - 56]	530 [99 - 1576]	76 [13 - 238]	2792 [2792 - 2792]	2716 [2554 - 2779]	8482 [1703 - 27770]
Nigeria	NGA	29264 [18918 - 41248]	2556 [523 - 7141]	71 [12 - 216]	1988 [373 - 5906]	844 [178 - 2360]	11329 [11329 - 11329]	10485 [8969 - 11151]	8818 [1660 - 29080]
Nicaragua	NIC	863 [554 - 1223]	75 [15 - 211]	2 [0 - 6]	64 [12 - 191]	55 [12 - 149]	356 [356 - 356]	302 [207 - 344]	7975 [1319 - 27058]
Nepal	NPL	3975 [2555 - 5630]	347 [71 - 973]	10 [2 - 29]	291 [54 - 868]	61 [12 - 174]	1573 [1573 - 1573]	1512 [1399 - 1561]	8634 [1701 - 28312]
Pakistan	PAK	38637 [24917 - 54545]	3375 [690 - 9440]	94 [16 - 285]	2791 [520 - 8297]	1445 [224 - 5285]	12967 [12967 - 12967]	11523 [7682 - 12744]	6917 [1179 - 22604]
Papua New Guinea	PNG	818 [527 - 1157]	71 [15 - 200]	2 [0 - 6]	59 [11 - 175]	62 [12 - 193]	473 [473 - 473]	410 [280 - 461]	11772 [2074 - 39852]
Democratic People's Republic of Korea	PRK	2436 [1565 - 3454]	213 [44 - 597]	6 [1 - 18]	178 [33 - 531]	24 [5 - 73]	1030 [1030 - 1030]	1007 [957 - 1026]	9351 [1889 - 30820]
Rwanda	RWA	2488 [1602 - 3521]	217 [44 - 609]	6 [1 - 18]	180 [33 - 536]	45 [9 - 130]	1072 [1072 - 1072]	1027 [942 - 1063]	9480 [1876 - 31129]
Sudan	SDN	9469 [6101 - 13382]	827 [169 - 2315]	23 [4 - 70]	677 [126 - 2012]	495 [90 - 1424]	3616 [3616 - 3616]	3121 [2192 - 3526]	7787 [1318 - 25824]
Senegal	SEN	4735 [3047 - 6703]	414 [84 - 1159]	12 [2 - 35]	342 [64 - 1019]	133 [29 - 394]	1555 [1555 - 1555]	1422 [1161 - 1527]	6957 [1269 - 23333]
Solomon Islands	SLB	75 [48 - 106]	7 [1 - 18]	0 [0 - 1]	5 [1 - 16]	9 [2 - 26]	41 [41 - 41]	31 [14 - 39]	Inf [1189 - 37242]

Sierra Leone	SLE	1648 [1066 - 2320]	144 [29 - 402]	4 [1 - 12]	111 [21 - 328]	33 [6 - 99]	653 [653 - 653]	620 [553 - 647]	9278 [1833 - 30326]
Somalia	SOM	2248 [1454 - 3166]	196 [40 - 548]	5 [1 - 17]	156 [29 - 462]	10 [3 - 26]	1006 [1006 - 1006]	996 [981 - 1003]	10581 [2129 - 34505]
South Sudan	SSD	1642 [1061 - 2314]	143 [29 - 401]	4 [1 - 12]	114 [21 - 339]	85 [16 - 240]	641 [641 - 641]	556 [401 - 624]	8210 [1411 - 27153]
Sao Tome and Principe	STP	43 [27 - 60]	4 [1 - 10]	0 [0 - 1]	3 [0 - 9]	2 [0 - 6]	18 [18 - 18]	16 [12 - 18]	Inf [1629 - Inf]
Chad	TCD	3326 [2155 - 4679]	290 [59 - 810]	8 [1 - 24]	225 [42 - 668]	44 [9 - 125]	1258 [1258 - 1258]	1214 [1132 - 1249]	8956 [1754 - 29452]
Togo	TGO	1540 [993 - 2175]	135 [27 - 376]	4 [1 - 12]	108 [20 - 321]	23 [5 - 64]	573 [573 - 573]	550 [509 - 568]	8460 [1674 - 27814]
Tajikistan	TJK	1752 [1127 - 2480]	153 [31 - 429]	4 [1 - 13]	128 [24 - 382]	42 [8 - 123]	723 [723 - 723]	681 [601 - 715]	8838 [1721 - 29013]
Timor-Leste	TLS	242 [156 - 342]	21 [4 - 59]	1 [0 - 2]	18 [3 - 52]	12 [2 - 36]	124 [124 - 124]	112 [88 - 122]	10728 [1983 - 37854]
United Republic of Tanzania	TZA	16279 [10480 - 23026]	1422 [290 - 3982]	40 [7 - 120]	1175 [219 - 3492]	216 [43 - 624]	6219 [6219 - 6219]	6003 [5595 - 6176]	8501 [1674 - 27856]
Uganda	UGA	12764 [8233 - 18017]	1115 [227 - 3119]	31 [5 - 94]	895 [167 - 2660]	283 [53 - 864]	4784 [4784 - 4784]	4502 [3921 - 4732]	8357 [1657 - 27613]
Ukraine	UKR	2587 [1660 - 3669]	226 [46 - 633]	6 [1 - 19]	189 [35 - 565]	256 [48 - 768]	1061 [1061 - 1061]	805 [293 - 1012]	7323 [823 - 25745]
Uzbekistan	UZB	4651 [2990 - 6588]	406 [83 - 1138]	11 [2 - 34]	341 [64 - 1014]	325 [65 - 937]	1918 [1918 - 1918]	1593 [981 - 1853]	7955 [1229 - 26787]
Viet Nam	VNM	7288 [4682 - 10330]	637 [130 - 1784]	18 [3 - 54]	544 [101 - 1621]	69 [23 - 157]	4483 [4483 - 4483]	4414 [4327 - 4460]	13485 [2691 - 43993]
Yemen	YEM	4798 [3089 - 6786]	419 [86 - 1174]	12 [2 - 36]	343 [64 - 1020]	198 [45 - 540]	1809 [1809 - 1809]	1611 [1269 - 1764]	7933 [1391 - 26692]
Zambia	ZMB	4914 [3166 - 6944]	429 [88 - 1201]	12 [2 - 36]	348 [65 - 1034]	263 [72 - 702]	1851 [1851 - 1851]	1588 [1149 - 1779]	7725 [1302 - 26228]
Zimbabwe	ZWE	3479 [2241 - 4920]	304 [62 - 851]	8 [1 - 26]	244 [46 - 727]	70 [14 - 202]	1449 [1449 - 1449]	1379 [1246 - 1435]	9375 [1836 - 30763]

Country-specific estimated mean [95% credible interval] of undiscounted number of RSV cases, hospital admissions, deaths and discounted costs (in thousand) and DALY averted, average cost-effectiveness ration in 2020. Costs are presented in USD 2016 values. Discount rate is 3% per year
